# Flexibility‐Aided Orientational Self‐Sorting and Transformations of Bioactive Homochiral Cuboctahedron Pd_12_L_16_

**DOI:** 10.1002/anie.202513902

**Published:** 2025-08-10

**Authors:** Subhasis Chattopadhyay, Robin Durník, Anniina Kiesilä, Elina Kalenius, Juha M. Linnanto, Pavel Babica, Jan Kuta, Radek Marek, Ondřej Jurček

**Affiliations:** ^1^ Department of Chemistry Faculty of Science Masaryk University Kamenice 5 Brno CZ‐62500 Czechia; ^2^ Department of Natural Drugs Faculty of Pharmacy Masaryk University Palackého 1946/1 Brno CZ‐61200 Czechia; ^3^ CEITEC–Central European Institute of Technology Masaryk University Kamenice 5 Brno CZ‐62500 Czechia; ^4^ Department of Biochemistry Faculty of Science Masaryk University Kamenice 5 Brno CZ‐62500 Czechia; ^5^ RECETOX Faculty of Science Masaryk University Kotlarska 2 Brno CZ‐61137 Czechia; ^6^ Department of Chemistry University of Jyvaskyla P. O. Box 35 Jyväskylä FI‐40014 Finland; ^7^ Institute of Physics University of Tartu W. Ostwald Street 1 Tartu 50411 Estonia; ^8^ National Center for Biomolecular Research Faculty of Science Masaryk University Kamenice 5 Brno CZ‐62500 Czechia

**Keywords:** Biological activity, Chirality, Self‐assembly, Structural transformation, Supramolecular chemistry

## Abstract

The rational design and selective self‐assembly of flexible and unsymmetric ligands into large coordination complexes is an eminent challenge in supramolecular coordination chemistry. Here, we present the coordination‐driven self‐assembly of natural ursodeoxycholic‐bile‐acid‐derived unsymmetric *tris*‐pyridyl ligand (**L**) resulting in the selective and switchable formation of chiral stellated Pd_6_ **L**_8_ and Pd_12_ **L**_16_ cages. The selectivity of the cage originates in the adaptivity and flexibility of the arms of the ligand bearing pyridyl moieties. The interspecific transformations can be controlled by changes in the reaction conditions. The orientational self‐sorting of **L** into a single constitutional isomer of each cage, i.e., homochiral quadruple and octuple right‐handed helical species, was confirmed by a combination of molecular modelling and circular dichroism. The cages, derived from natural amphiphilic transport molecules, mediate the higher cellular uptake and increase the anticancer activity of bioactive palladium cations as determined in studies using in vitro 3D spheroids of the human hepatic cells HepG2.

## Graphical abstract

Mimicking concave morphologies found in nature, we have introduced flexible, unsymmetric, tridentate ligand (**L**) which undergoes coordination self‐assembly into highly organized chiral Pd_6_ **L**_8_ or Pd_12_ **L**_16_ cages that can undergo interspecific transformations. The cages, being based on natural amphiphilic transport molecules, mediate high cellular uptake and increase the anticancer activity of bioactive palladium cations.

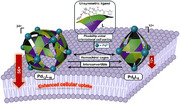

## Introduction

Natural chiral hydrophobic cavities are important for many biological functions, e.g., for recognition as parts of transport proteins or for substrate‐specific transformations as parts of enzymes. To understand and mimic these natural systems and their (supra)molecular mechanisms of action, the development of their artificial counterparts (e.g., cages, macrocycles) from chiral molecules is desirable. Easing such efforts, nature readily offers convenient chiral building blocks (terpenoids, amino acids, or carbohydrates) which can be utilized. Intermolecular‐interaction‐mediated self‐assembly together with metal coordination are essential natural processes to construct such higher‐order structures and can easily be adapted in the development of artificial systems.

The discovery of supramolecular coordination cages (SCCs) via coordination‐driven self‐assembly by Saalfrank et al.^[^ ^1^ ^]^ led to the rapid development of a large group of metallo‐cycles and metallo‐cages mainly using rigid and symmetric *bis*/*tris*‐pyridyl coordinating ligands and tetravalent square‐planar Pd^2+^.^[^ ^2^ ^]^ The edge‐directed self‐assembly of different *bis*‐pyridyl ligands resulted in the formation of SCCs and macrocycles with the general formula Pd_n_L_2n_, e.g., Pd_2_L_4_, Pd_3_L_6_, Pd_4_L_8_, Pd_6_L_12_, Pd_12_L_24_, Pd_24_L_48_, Pd_30_L_60_, or Pd_48_L_96_– the largest SCC described so far.^[^ ^3^ ^]^ Whereas *tris*‐pyridyl ligands led to Pd_3n_L_4n_ SCCs, with only four types so far, i.e., Pd_3_L_4_,^[^ ^4^, ^5^, ^6^ ^]^ Pd_9_L_12_ (only one),^[^ ^7^ ^]^ Pd_18_L_24_^[^ ^8^ ^]^ (one) via edge‐directed self‐assembly, and the most common Pd_6_L_8_^[^ ^9^ ^]^ via face‐directed self‐assembly.

More than three decades after the first discovery, most of these metallo‐supramolecular complexes are achiral and symmetric. Several approaches have been employed to construct low‐symmetry, unsymmetric, or chiral coordination complexes using unsymmetric bidentate ligands,^[^ ^10^ ^]^ a combination of multiple symmetric ligands (heteroleptic complexes),^[^ ^11^, ^12^ ^]^ or even single‐type symmetric ligands.^[^ ^13^, ^14^, ^15^, ^16^ ^]^ However, unlike natural systems, the presence of stereogenic carbons in the structure of ligands is rare, mostly limited to the peripheral areas of ligands and their resulting complexes,^[^ ^17^ ^]^ e.g., using peptides,^[^ ^18^, ^19^ ^]^ pentasaccharide,^[^ ^20^ ^]^ or short alkyl chains.^[^ ^21^ ^]^

To mimic the natural systems more closely and to develop the “next generation” SCCs, where the chiral centres will line their inner cavities requires careful design of ligands from inherently chiral natural compounds. Following this concept, Jurček et al. introduced the metallo‐supramolecular macrocycle Pd_3_L_6_ containing 60  stereogenic carbons using the natural bile acid (BA) ursodeoxycholic acid (UDCA) as a core for *bis*‐pyridyl ligands.^[^ ^22^, ^23^ ^]^ A comparable study reports Pd_2_L_4_ SCCs using cholic‐, deoxycholic‐, or lithocholic‐acid‐based *bis*‐pyridyl ligands.^[^ ^24^ ^]^ Recently, it was shown that ligands derived from chenodeoxycholic acid, an epimer of UDCA, can form even larger complexes, namely, Pd_2_L_4_, Pd_3_L_6_, Pd_4_L_8_, Pd_5_L_10_, and Pd_6_L_12,_ having 120 stereogenic carbons.^[^ ^25^ ^]^ Other than this, intriguing coordination complexes have been reported using peptide‐based *bis*‐pyridyl ligands in the process of folding and assembly,^[^ ^26^ ^]^ but leaving tritopic natural‐molecule‐based ligands unstudied.

Moreover, most ligands used to build coordination complexes are rather small and rigid. The directionality of the binding sites together with the rigidity of the ligand predefine their bend angles. These characteristics are crucial to control the self‐assembly processes of symmetric and rigid ligands. Small differences in the bend angle of the ligand lead to significant changes in the final self‐assembly products, e.g., a difference of 3° in the bend angle leads to the Pd_24_L_48_→Pd_48_L_96_ or Pd_12_L_24_→Pd_24_L_48_ transformation.^[^ ^27^, ^28^, ^29^ ^]^ However, the use of small, and to some extent flexible, ligands typically results in a mixture of kinetically trapped coordination complexes.^[^ ^30^ ^]^ In contrast, the large, flexible, and unsymmetric UDCA‐based *bis*‐pyridyl ligands, with a deviation of their bend angle up to 30° (90°–120°), showed the selective formation of a single constitutional isomer of Pd_3_L_6_.^[^ ^22^, ^23^, ^25^ ^]^ In comparison, the epimeric flexible chenodeoxycholic‐acid‐based ligand spans a bend angle range of 70°–90° (Δ 20°) resulting in a mixture of macrocyclic complexes (Pd_2_L_4_, Pd_3_L_6_, Pd_4_L_8_, Pd_5_L_10_, and Pd_6_L_12_). It has been proposed that the greater flexibility of the unsymmetric ligand together with certain steric restrictions increase the probability of forming a single species via orientational self‐sorting.^[^ ^25^ ^]^ Still, a better understanding of the effect of the structural behaviour of the ligand on the final self‐assembly is required.

Even after a rapid increase in the number of architectonically appealing SCCs, studies of their biomedical aspects and applications are limited.^[^ ^31^ ^]^ However, recent contributions demonstrate their drug‐loading ability and promising anticancer therapeutical potential.^[^ ^32^, ^33^ ^]^ From this perspective, the utilization of natural molecules in the construction of biocompatible SCCs is appealing. Out of the vast library of natural compounds, BAs meet well the requirements for the design of chiral, unsymmetric, flexible, and biocompatible ligands. The BAs are biosynthesized in the human body, where they play a key role in the digestion and transport of lipids and lipid‐soluble nutrients within the enterohepatic circulation using various passive and active transport processes.^[^ ^34^ ^]^ BAs are commercially easily available, enantiomerically pure (containing 9–11 chiral centres), and possess a conformationally defined rigid steroid skeleton decorated with hydroxyl groups and a flexible alkyl side chain bearing a carboxylic acid group, that can be easily synthetically transformed into pyridyl coordination sites.^[^ ^22^, ^23^, ^24^, ^25^ ^]^ However, preparation of the *tris*‐pyridyl ligand from a BA, its coordination‐driven self‐assembly with square‐planar tetravalent Pd^2+^, demonstration of the potential of the ligand for orientational self‐sorting and flexibility‐permitted selective self‐assembly, together with evaluation of its biomedical potential have been missing until now.

## Results and Discussion

**Figure 1:**
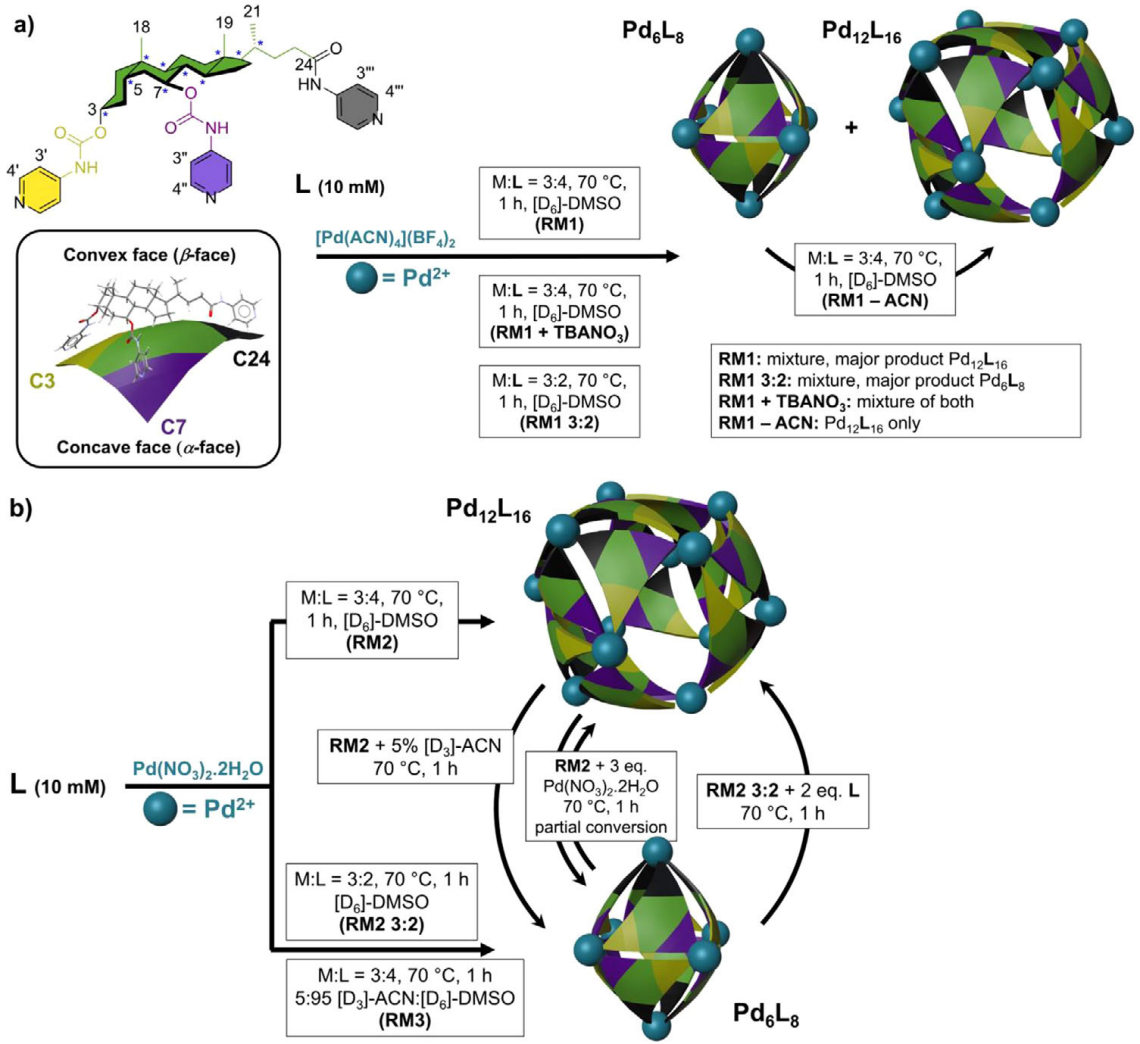
Coordination‐driven self‐assembly of **L** into stellated helical octahedral Pd_6_ **L**_8_ and cuboctahedral Pd_12_ **L**_16_ SCCs and their transformation reactions: a) using [Pd(ACN)_4_](BF_4_)_2,_ b) using Pd(NO_3_)_2_. The blue asterisk denotes chiral centres of the steroid skeleton.

Natural UDCA was decorated in four synthetic steps with three 4‐aminopyridine groups, attached either through carbamate bonds to C3 and C7 hydroxyls or by an amide bond to the C24 carboxylic acid, resulting in a tridentate ligand (**L**) (Figure 1, Supporting Information section 2.1). A molecular model of **L** can be visualized as an elongated triangular panel (towards C24) having two faces, concave (*α*) and convex (*β*), containing either pyridyls (Py) or methyls (C18, C19, and C21), respectively (Figure 1).

**Figure 2:**
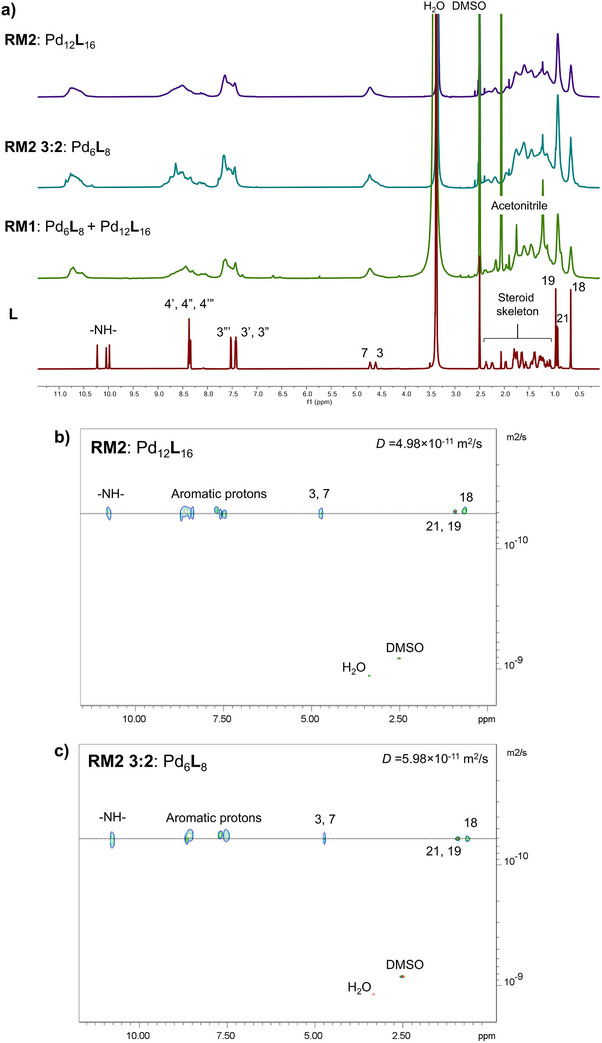
NMR characterisation of Pd_6_ **L**_8_ and Pd_12_L_16_. a) ^1^H NMR spectra of **L**, mixture of Pd_6_ **L**_8_ and Pd_12_ **L**_16_ (**RM1**), Pd_6_ **L**_8_ (**RM2 3:2**), and Pd_12_ **L**_16_ (**RM2**) in [D_6_]‐DMSO at 298.2 K and 700 MHz. ^1^H DOSY NMR spectra of b) Pd_12_ **L**_16_ (**RM2**) and c) Pd_6_ **L**_8_ (**RM2 3:2**) ([D_6_]‐DMSO, 303.2 K and 700 MHz).

The initial complexation of **L** (10 mM) with [Pd(ACN)_4_](BF_4_)_2_ (metal to ligand ratio M:**L** 3:4) in [D_6_]‐DMSO (70 °C, 1 h), marked as reaction mixture 1 (RM1) (Figure 1a), led to a quantitative (i.e., the spectrum lacks signals corresponding to the non‐coordinated free ligand) formation of coordination species as confirmed by ^1^H NMR spectroscopy (Figure 2a, green, Figure S13). The ^1^H NMR signals showed a high‐frequency coordination shift. The significant broadening of the ^1^H signals and the presence of multiple aromatic signals give rise to a few possibilities considering the unsymmetry and flexibility of **L**, the formation of: 1) a mixture of coordination complexes with varying molecular formula (size), 2) multiple architectures of a complex having the same molecular formula, 3) multiple constitutional isomers of a single architecture (varying C3‐C7‐C24‐pyridyl‐Pd^2+^ connectivity–rotation of triangular panel), 4) multiple conformational isomers of a coordination complex, or 5) a combination of the abovementioned possibilities.

In the next step, the reaction mixture was analysed by electrospray ionization (ESI‐MS) and ion mobility mass spectrometry (IM‐MS) (Figure S14) revealing charge state distributions for ions [Pd_6_ **L**_8_(BF_4_)_n_]^(12‐n)+^ (*n* = 1–8) and [Pd_12_ **L**_16_(BF_4_)_n_]^(24‐n)+^ (*n* = 6–16) (Table S1, being in the size range of small proteins, ca 7 and 14 kDa, respectively). The drift tube collision cross sections in nitrogen (^DT^CCS_N2_) of [Pd_12_ **L**_16_(BF_4_)_14_]^10+^ and [Pd_6_ **L**_8_(BF_4_)_2_]^10+^ were 1976 and 1420 Å^2^, corresponding roughly to diameters of 5.0 and 4.3 nm, respectively. According to the IM‐MS, the formation of the larger cage Pd_12_ **L**_16_ was more abundant under these conditions (∼75% of Pd_12_ **L**_16_ compared to Pd_6_ **L**_8_). Furthermore, the number of species and their diffusion coefficients (*D*) were determined by ^1^H DOSY NMR (Figure S15).

The presence of two species was confirmed, and hydrodynamic diameters were calculated from the diffusion coefficients using the Stokes–Einstein equation, 4.8 nm (Pd_6_ **L**_8_) and 5.8 nm (Pd_12_ **L**_16_). This provided a moderate fit with the sizes observed using the IM‐MS data (Table S1) and confirmed the formation of Pd_6_ **L**_8_ and Pd_12_ **L**_16_ SCCs. Additionally, MS analysis confirmed that Pd_12_ **L**_16_ is a monomer rather than a dimer of Pd_6_ **L**_8_ based on the specific intensity abundance, concentration independence, collision cross section, and the presence of odd charge states of the ions (Supporting Information Section 2.2.1).

The selective formation of the Pd_12_ **L**_16_ cage was achieved by dissolving **L** in [D_6_]‐DMSO (10 mM) and using a different metal salt Pd(NO_3_)_2_ at M:**L** 3:4 (70 °C, 1 h) (RM2; Figures 1, 2,b, Supporting Information Section 2.2.3). The selective formation of the Pd_6_ **L**_8_ cage was achieved under two different reaction conditions, where **L** (10 mM) reacts with: 1) Pd(NO_3_)_2_ at M:**L** 3:2 ratio in [D_6_]‐DMSO (70 °C, 1 h) (RM2 3:2; Figures 1, 2,c, Supporting Information Section 2.2.3); and 2) Pd(NO_3_)_2_ at M:**L** 3:4 in the solvent mixture [D_3_]‐ACN:[D_6_]‐DMSO 5:95 (70 °C, 1 h) (RM3; Supporting Information Section 2.2.5). The solvent system was adjusted according to the low solubility of **L** in neat [D_3_]‐ACN. The ^1^H NMR spectra of both cages in [D_6_]‐DMSO were distinguished by a sharp singlet signal at about 8.7 ppm indicating the presence of Pd_6_ **L**_8_ (Figure 2a). The ^1^H DOSY NMR experiments then reliably confirmed the selective formation of Pd_12_ **L**_16_ or Pd_6_ **L**_8_ in both solvent systems (Table S3). The values of the diffusion coefficient (*D*) for products in RM2, RM2 3:2, and RM3 were 4.98^−11^ m^2^ s^−1^ (4.9 nm, Figure 2b), 5.98 × 10^−11^ m^2^ s^−1^ (4 nm, Figure 2c), and 6.24 × 10^−11^ m^2^ s^−1^ (the presence of [D_3_]‐ACN slightly increases *D*, Figure S33), respectively.

In the next step, we investigated a possible interspecific transformation of the coordination species followed by ^1^H NMR and ^1^H DOSY NMR techniques. Post‐synthetic addition of 5% (*v/v*) [D_3_]‐ACN to the [D_6_]‐DMSO solution of Pd_12_ **L**_16_ (RM2) (heated at 70 °C, 1 h) led to complete conversion to Pd_6_ **L**_8_ (Supporting Information Section 2.2.6.1). In another experiment, adding two equivalents of **L** to the [D_6_]‐DMSO solution of Pd_6_ **L**_8_ (RM2 3:2), changing the M:**L** ratio from 3:2 to 3:4 (heated at 70 °C, 1 h), led to complete conversion to Pd_12_ **L**_16_ (Supporting Information Section 2.2.6.2). Conversely, three equivalents of Pd(NO_3_)_2_ were added to the Pd_12_ **L**_16_ solution (RM2), changing the ratio M:**L** from 3:4 to 3:2 (heated at 70 °C, 1 h). Spectroscopic analyses showed partial conversion of Pd_12_ **L**_16_ to Pd_6_ **L**_8_ (Supporting Information Section 2.2.6.3), even after prolonged heating. A comparison of the diffusion coefficients for all reaction products is shown in Table S3.

Considering these observations and the related literature,^[^ ^35^, ^36^ ^]^ we suggest a hypothesis for the reaction mechanism of self‐assembly processes. The coordination effect of the coordinating species participating in the reaction mixture decreases in the sequence: pyridyl >> DMSO > NO_3_^‐^ > acetonitrile >> BF_4_^−^.^[^ ^37^ ^]^ At the same time, their coordination ability should also be considered through their concentration factors, thus the effect of [D_6_]‐DMSO, present in all experiments in very high excess, can be considered as comparable. Therefore, we hypothesize that the **L**‐pyridyls↔NO_3_^−^↔acetonitrile exchange affects the self‐assembly processes the most significantly (ignoring the effect of the weak coordination of BF_4_^−^). A reaction mixture may contain a Pd_6_ **L**_8_ self‐assembly as a minor product in a mixture with Pd_12_ **L**_16_ if [Pd(ACN)_4_](BF_4_)_2_ (3:4 M:**L**, Pd:ACN ratio 1:4) was used or as neat single product when using Pd(NO_3_)_2_ with 5% (*v/v*) [D_3_]‐ACN in [D_6_]‐DMSO as the solvent system (Pd:ACN ratio 1:128). In the case of [Pd(ACN)_4_](BF_4_)_2_, Pd_6_ **L**_8_ is present as a minor product in a mixture with Pd_12_ **L**_16_ (as determined by comparison of the heights of the drift peaks by IM‐MS, Table S1), whereas in a large excess of ACN, Pd_6_ **L**_8_ is the only species. Also, prolonged heating of the [Pd(ACN)_4_](BF_4_)_2_ reaction leads to an increase in the concentration of Pd_6_ **L**_8_. In addition, the reaction of **L** (10 mM) with [Pd(ACN)_4_](BF_4_)_2_ at M:**L** 3:2 in [D_6_]‐DMSO (70 °C, 1 h) was carried out, where the Pd_6_ **L**_8_ species was observed as the major product in a mixture with Pd_12_ **L**_16_ (Table S2, Figure 1a). In general, the higher the concentration of ACN the higher the ratio of Pd_6_ **L**_8_ to Pd_12_ **L**_16_. Finally, we also performed an experiment where the product was prepared via RM1 and the reaction mixture was subsequently evaporated to dryness under vacuum to remove/reduce the ACN content. The solid residue was re‐dissolved in [D_6_]‐DMSO at 70 °C (RM1–ACN). ^1^H NMR spectroscopy showed only a trace amount of ACN remaining (the ratio between Pd:ACN was reduced from 1:4 to 1:0.2, Figure S16). The ^1^H‐DOSY NMR spectrum showed complete conversion of the Pd_12_ **L**_16_ and Pd_6_ **L**_8_ mixture into Pd_12_ **L**_16_ only (Figure S17), which further confirms that it is the elevated amount of ACN that directs the reaction towards Pd_6_ **L**_8_. In this experiment, we could also compare the effect of a palladium counter‐anion, having the reaction mixture containing either BF_4_^−^ or NO_3_^−^ anion in [D_6_]‐DMSO in [M]:[L] = 3:4 ratio. Both reaction mixtures provided the Pd_12_ **L**_16_ complex, suggesting that the anion effect on the final self‐assembly is similar or negligible, unlike the eminent effect of ACN.

A similar situation can also be observed in the case of varying Pd(NO_3_)_2_ concentration, i.e., a higher concentration of Pd(NO_3_)_2_ in RM2 3:2 leads to selective formation of Pd_6_ **L**_8_ in comparison to RM2, or the addition of an excessive amount of Pd(NO_3_)_2_ to Pd_12_ **L**_16_ (prepared under conditions of RM2), shifting the molar ratio M:**L** from 3:4 to 3:2 (resulting in three equivalents of Pd(NO_3_)_2_ present as a free salt in the reaction mixture), leads to a partial transformation to Pd_6_ **L**_8_. In contrast, the addition of **L** to RM2 3:2, shifting the M:**L** from 3:2 to 3:4 (decreasing the concentration of free Pd(NO_3_)_2_), promotes the formation of Pd_12_ **L**_16_.

To further distinguish the effect of nitrate or palladium, we have carried out additions of TBANO_3_ to RM1 (Supporting Information Section 2.2.2.2). 1.5 eq. (M:L:NO_3_^‐^ 3:4:6) and 3 eq. (M:L:NO_3_^‐^ 3:4:12) of TBANO_3_ with respect to **L** were subsequently added and the reaction mixture was heated at 70 °C for 1 h upon every addition (Figure S18a,b). ^1^H DOSY NMR analysis showed that the solutions contained mixtures of SCCs (even after continued heating for 24 h) (Figure S18c). These experiments indicate that the addition of nitrate does not influence strongly the equilibrium between the SCCs and it is rather the palladium concentration or the ACN content that control the interspatial transformation and direct the equilibrium towards the Pd_6_ **L**_8_ product.

The presence of two different species in the reaction mixture suggests the formation of thermodynamic and kinetically trapped species. A previous study by Hiraoka et al. showed that the use of ACN as solvent accelerates the ligand exchange rate (solvent‐assisted exchange self‐assembly process) which prevents the formation of primitive intermediates leading to kinetic products, thus it is promoting the formation of a thermodynamic product.^[^ ^36^ ^]^ The participation of ACN in the self‐assembly process results in preference for the Pd_6_ **L**_8_ complex which can thus be seen as a thermodynamic product, whereas its absence in DMSO shifts the equilibrium towards the kinetic product Pd_12_ **L**_16_.

If we consider that ACN allows greater coordination bond reversibility and increases the dynamic character of the Pd(II)‐ligand coordination bond, a mechanism of reaction selectivity could be deduced. In the process of self‐assembly, ligands form variously sized oligomers with metals before the final transformation into a cage (cyclization).^[^ ^38^ ^]^ In the presence of an increased concentration of ACN, the more dynamic ligand exchange can be responsible for the formation of a greater number of smaller oligomers, ultimately leading to a smaller Pd_6_ **L**_8_ complex. This can also be true with an elevated concentration of Pd(NO_3_)_2_ where the increased concentration of palladium cations plays a key role in the formation of the oligomer (the higher the Pd^2+^ concentration, the smaller the oligomer and the smaller the final complex). In contrast, in the absence of ACN, the oligomers grow larger as the ligand exchange is slower, ultimately leading to large kinetically trapped oligomers which eventually transform into Pd_12_ **L**_16_.

**Figure 3:**
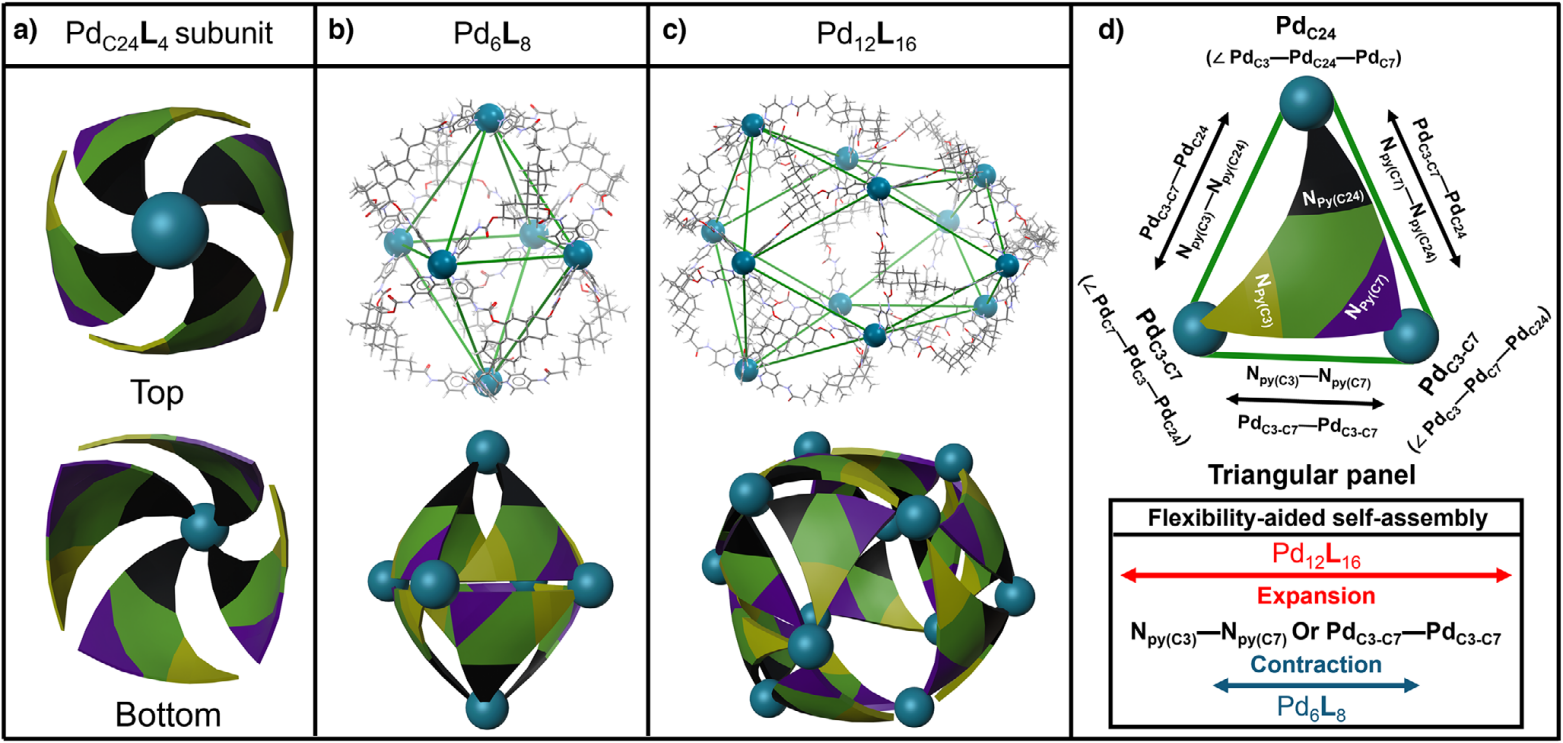
Computational models and cartoon representations. a) Pd_C24_ **L**_4_ building subunit, b) Pd_6_ **L**_8_, c) Pd_12_ **L**_16_, and d) nomenclatures used for the triangular panel.

Nuclear shielding in diamagnetic systems is highly local in nature, therefore the measured ^1^H NMR shifts for ligands in Pd_6_ **L**_8_ and Pd_12_ **L**_16_ are very similar, also being composed of similarly built subunits (Figure 3a). However, the larger Pd_12_ **L**_16_ cage has a slightly longer correlation time (shorter relaxation time) and therefore somewhat broader NMR lines (Figure 2a). Additionally, we assume that the line width and apparent presence of multiple sets for the proton NMR signals of pyridyls and the ‐NH‐ groups indicate the representation of multiple stable conformational isomers of **L** in the complexes. In this regard, a variable temperature ^1^H NMR study of RM2 was conducted over the temperature range 298.2–398.2 K in increments of 10 K (Figure S27, Supporting Information Section 2.2.4). The study shows sharpening of the proton signals with increasing temperature, but also the eventual appearance of a second set of signals to the detriment of the original set corresponding to Pd_12_ **L**_16_. This can represent either formation of conformational or constitutional isomers, or the formation of a new coordination product (these could not be confirmed by MS). At the end of the experiment, the sample was cooled back down to 298.2 K and the ^1^H‐ and ^1^H DOSY NMR spectra that were recorded showed the presence of a single coordination species Pd_12_ **L**_16_ but represented by somehow sharper and better distinguished ^1^H NMR signals (Figures S27 and S28). For comparison, the reaction RM2 was carried out (M:L 3:4, 10 mM **L**, Pd(NO_3_)_2_, [d_6_]‐DMSO) at 100 °C, heated for 1 h, and ^1^H‐ and ^1^H DOSY NMR spectra were recorded showing the formation of Pd_12_ **L**_16_ similar to the previous case of incremental heating to 125 °C (Figure S29).

To our understanding,^[^ ^39^ ^]^ the appearance of a complex second set of multiplets with a significant chemical shift refers to the formation of a new coordination self‐assembly, most likely a new constitutional isomer (caused by rotation of the ligand(s)). This is more probable than the formation of new conformational isomers, as we assume that the spectral changes would be more subtle in such a case. Moreover, the structural changes are represented in a smaller fraction of the species and are temporary as they are connected to the elevated temperature and the original form is restored upon cooling. In contrast, the signal sharpening which remains even upon cooling could be related to conformational changes, i.e., in direct synthesis at 70 °C for 1 h, some ligands are trapped in local minima, whereas heating to higher temperatures helps them reach global minima conformations corresponding better to complex spatial requirements (where they also remain upon cooling).

As we previously foretold, comparing the self‐assemblies of epimeric *bis*‐pyridyl ligands derived from UDCA and chenodeoxycholic acid^[^ ^25^ ^]^ the structural selectivity for the self‐assembly lies in the ligand's geometry and the flexibility provided by the bending of the ligand molecule but also by a conformational change through rotation on the C3‐O‐CO‐NH‐pyridine axis.

Concerning the present ligand **L**, we believe that even larger conformational changes in the near vicinity of pyridyls, i.e., on the C3‐O‐CO‐NH‐pyridine, C7‐O‐CO‐NH‐pyridine, and C24‐CO‐NH‐pyridine axes, are manifested in solution. The conformational flexibility was further demonstrated by experiments using the *mono*‐pyridyl ligands **L_M3_** and **L_M24_**, where the binding moiety is at either position C3 or C24, respectively. For more details, please see Supporting Information Section 4.

Finally, having both neat species in hand, many attempts were made to grow a single crystal, but none of the crystals obtained showed X‐ray diffraction. Therefore, a combination of circular dichroism (CD) and molecular modelling was employed as an alternative for the structural analysis at the molecular level. Computational models of Pd_6_ **L**_8_ and Pd_12_ **L**_16_ were prepared and their energy was optimized using the DFT approach (Figure 3). An important steric restraint, resulting from the presence of two faces of **L**, is that the convex face should always point outside of the complex. Also, the elongated shape of **L** towards C24 further restricts its orientation in the final self‐assembly. This implies that the structure of **L** prohibits the formation of many theoretically possible constitutional isomers of SCCs. Following this, the structural subunit Pd_C24_ **L**_4_ was constructed, where four ligands are connected to Pd^2+^ by their C24‐pyridyls (marked as Pd_C24_) with the *β‐*faces pointing outwards (Figure 3a, the model subunit was designed in correlation with a CD spectroscopic study which is described in detail later). Two of these subunits were interconnected through four Pd^2+^ ions with the remaining pyridyls at C3 and C7 (marked as Pd_C3‐C7_), leading to Pd_6_ **L**_8_. The resulting optimized structure of Pd_6_ **L**_8_ represents an axially elongated, symmetric, and stellated octahedron (Figure 3b). To further support such structural organization, we performed semiempirical calculations on the Pd_6_ **L**_8_ complex using the PM6 method (palladium charge balanced by two nitrates) to study the effect of the rotation of **L** on the structure (constitutional isomerism). Initially, we found that the rotation of **L** is geometrically preferred in the counterclockwise direction, whereas rotation in the clockwise direction leads to large distances between the pyridyls and the *tris*‐pyridyl‐coordinated Pd cation that significantly change the overall shape of the complex and increase its total energy by a few tens of kcal per mole. In contrast, the complex with the ligand counterclockwise‐rotated retains its original shape overall but leads to an energy increase of about 7 kcal mol^−1^ in comparison to the original symmetric complex suggested. If all the ligands in the complex were similarly rotated, the total energy would increase by about 40 kcal mol^−1^ (approximately 5 kcal mol^−1^ per ligand rotation). Finally, we compared the total energies of the ligands taken out of both optimized Pd_6_ **L**_8_ complexes (original and rotated). The differences in total energy between these two ligand conformations result similarly in about 4–6 kcal mol^−1^, suggesting that the energy difference between the complexes originates mostly in the conformational change of the ligand, while the energy of the coordination interactions between Pd(II) and the pyridyls of the ligand seem to be quite similar for both complexes.

The structure of Pd_12_ **L**_16_ was similarly constructed using four Pd_C24_ **L**_4_ subunits. Two Pd_C24_ **L**_4_ subunits were placed sideways to each other and connected by two Pd_C3‐C7_. Two of these dimeric units were then joined together with the remaining Pd_C3‐C7_ (resembling the structure of a tennis ball) and fulfilling the square planar coordination sphere by pyridyls forming the cuboctahedral Pd_12_ **L**_16_ species. The geometry‐optimized structure of Pd_12_ **L**_16_ illustrates a low‐symmetry stellated cuboctahedron, where each triangular face contains one **L**, two opposite square faces have two **Ls** each, and each remaining square face has one **L** in an alternating up and down (zig–zag) fashion (Figure 3c). This supramolecular cuboctahedron, cultivating the face‐directive self‐assembly of a tritopic ligand, is unprecedented.

The structural behaviour of **L** in Pd_6_ **L**_8_ and Pd_12_ **L**_16_ can be easily described and followed through the Pd–Pd distances. The average Pd_C24_—Pd_C3‐C7_ and Pd_C3‐C7_—Pd_C3‐C7_ distances for Pd_6_ **L**_8_ are 18.4 and 15.6 Å, and those of Pd_12_ **L**_16_ are 19.5 and 19.9 Å, respectively. The average angles of the triangular faces for Pd_6_ **L**_8_ are 50° (for ∠Pd_C3_—Pd_C24_—Pd_C7_) and 65° (for ∠Pd_C7_—Pd_C3_—Pd_C24_ and ∠Pd_C3_—Pd_C7_—Pd_C24_), whereas those of Pd_12_ **L**_16_ contain 60° in both cases (Figure 3d, Tables S4–S7). Thus, the octahedron is made of isosceles triangles, while the cuboctahedron is composed of nearly equilateral triangular panels. The Pd_3‐7_—Pd_3‐7_ distances correlate to the N_Py(C3)_—N_Py(C7)_ distance corresponding to a change of ∠N_Py(C3)_—C5—N_Py(C7)_ bend angle of **L**. The ∠N_Py(C3)_—C5—N_Py(C7)_ bend angle range is 90°–93° for Pd_6_ **L**_8_ and 105°–127° for Pd_12_ **L**_16_ (Figure S40). At the same time, a small increase in the Pd_C3‐C7_—Pd_C24_ distance for Pd_12_ **L**_16_ demonstrates the decrease in the convexity of **L**. In summary, although the ligand is an unsymmetric building block in its nature, inside the complexes it can effectively adapt because of its flexibility and it is contained as a regular symmetric panel according to the steric and geometric requirements of the given self‐assembly (resembling the multicomponent self‐assembly process of viral capsids). In particular, **L** adapts to the structure of a stellated octahedron or cuboctahedron in directions N_Py(C3)_—N_Py(C7)_, N_Py(C3)_—N_Py(C24)_, N_Py(C7)_—N_Py(C24)_ differing by 3.1, 1.0, and 0.8 Å, with respect to each other, with higher standard deviations observed for Pd_12_ **L**_16_ (Figure 3d, Table S8). In comparison, the DFT‐optimized model of **L** (ground state in polar solvent, B3LYP/6–31G*) has a medial N_Py(C3)_—N_Py(C7)_ distance to both species, an N_Py(C7)_—N_Py(C24)_ distance similar to that of Pd_6_ **L**_8_, and is 0.8 Å longer than in Pd_12_ **L**_16_, but it is significantly stretched in the direction N_Py(C3)_—N_Py(C24)_ by 5.0 and 4.0 Å as compared to Pd_6_ **L**_8_ and Pd_12_ **L**_16_, respectively (Table S8).

The selective formation of different complexes using a single usually small and relatively rigid ligand were previously achieved by: 1) a templating effect of the solvent^[^ ^40^ ^]^ or counter anion,^[^ ^41^, ^42^ ^]^ 2) using different metal nodes (i.e., Pt^2+^ and Pd^2+^),^[^ ^43^ ^]^ 3) using different binding sites of the same ligand,^[^ ^44^ ^]^ or 4) using photoactive ligands via changing their conformation.^[^ ^45^ ^]^ Fine balancing and prediction of the structural flexibility in the design of a ligand is a challenging task (often resulting in a mixture of kinetically trapped species with limited flexibility)^[^ ^30^, ^43^ ^]^ and the construction of larger species with flexible ligands rather relies on the method of trial‐and‐error. Therefore, the selective formation of coordination complexes utilizing the inherent flexibility of the ligand represents a novel approach, which we refer to as *flexibility‐aided self‐assembly*, moreover, considering the unsymmetry of the ligand, we propose to term this phenomenon *flexibility‐aided orientational self‐sorting*.

**Figure 4:**
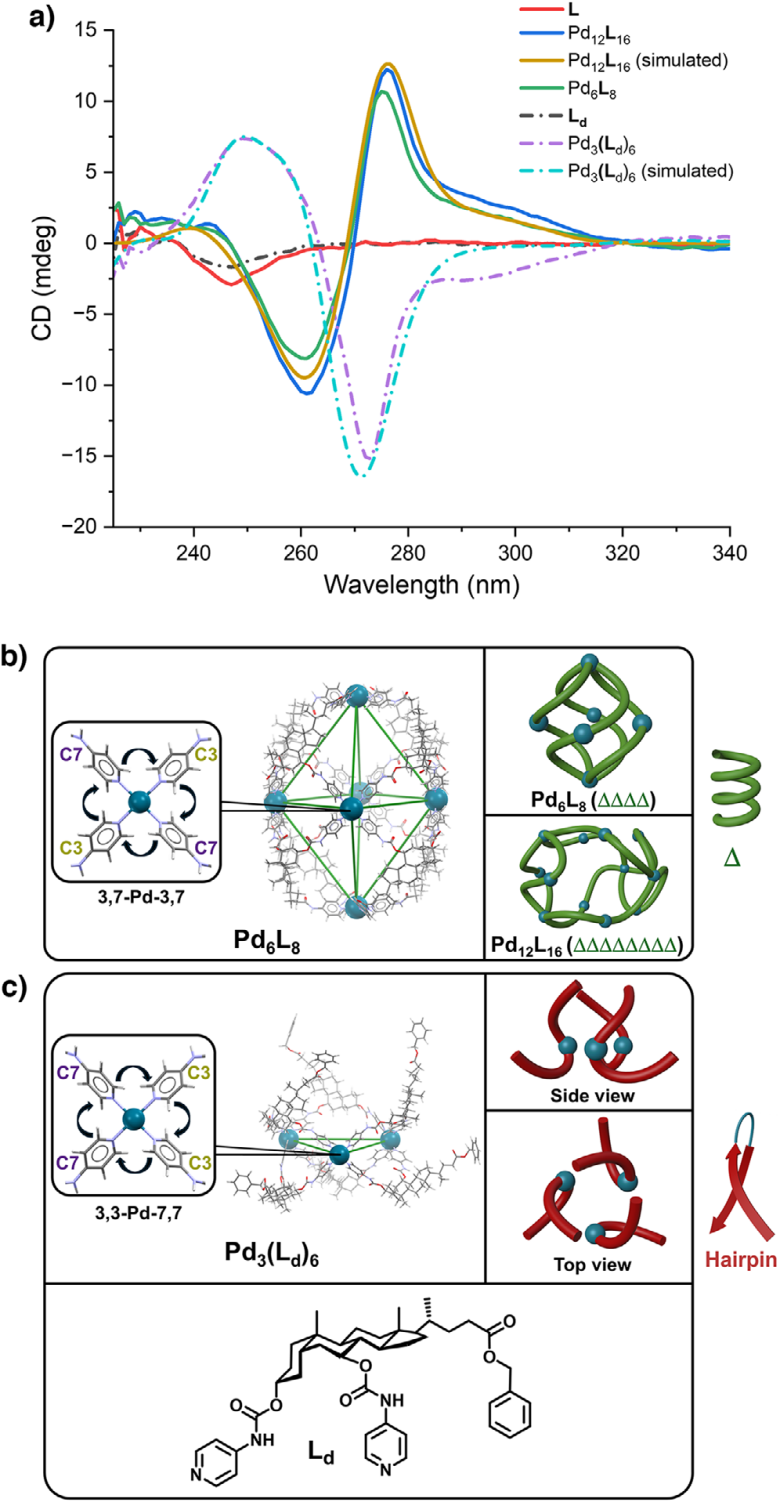
Structural analysis of supramolecular coordination complexes using CD spectroscopy. a) CD spectra of ligands and their coordination complexes in methanol at 25 °C. Interpretation of helical structures of b) Pd_6_ **L**_8_ or Pd_12_ **L**_16_, and c) Pd_3_(**L_d_**)_6_, following the C24‐C3‐Pd‐C3‐C24 backbone.

Finally, the CD spectra of **L**, Pd_6_ **L**_8_, and Pd_12_ **L**_16_ were recorded to investigate the chirality of the SCCs (Figure 4a). **L** gives a single negative CD band at 247 nm in the spectral region studied, whereas Pd_6_ **L**_8_ and Pd_12_ **L**_16_ display a strong positive band at 275 nm and strong negative band at 260 nm, resulting in a (+ −) CD couplet. The coordination sphere of Pd_C3‐C7_ in models of Pd_6_ **L**_8_ and Pd_12_ **L**_16_ shows a clockwise C3‐C7‐C3‐C7 connectivity (3,7‐Pd_C3‐C7_‐3,7) which induces right‐handed helicity (Δ), as observed on following two diagonally interconnected ligands through the C24‐C3‐Pd‐C3‐C24 backbone (Figures 4b and S41). This helicity is present in both cages, leading overall to a right‐handed quadruple (ΔΔΔΔ) for Pd_6_ **L**_8_ or octuple helices (ΔΔΔΔΔΔΔΔ) for Pd_12_ **L**_16_. In general, the structural organization of natural polymers into a right‐handed double helix is quite abundant, e.g., the most common form of DNA, canonical B‐DNA, is represented by a similar (+ −) CD couplet.^[^ ^46^ ^]^ The CD spectra of the SCCs were further compared with the previously reported *bis*‐pyridyl UDCA‐based ligand **L_d_** and its macrocyclic complex Pd_3_(**L_d_**)_6_^[^ ^22^ ^]^ (Figure 4c) where, interestingly, almost the opposite (− +) CD couplet was observed (Figure 4a). It was demonstrated that a single crown‐like constitutional isomer of Pd_3_(**L_d_**)_6_ was formed where the Pd^2+^ centres have a different clockwise connectivity C3‐C3‐C7‐C7 (3,3‐Pd‐7,7) (Figure 4c). If, similarly, the curvature of the C24‐C3‐Pd‐C3‐C24 backbone is followed, a hairpin structure is observed. This makes Pd_3_(**L_d_**)_6_ an ensemble of three hairpin subunits accounting for the opposite CD couplet. Interestingly, a similar inverse CD couplet can also be observed in nature representing the duplex‐to‐hairpin transformation of B‐DNA.^[^ ^46^ ^]^

To further support the structural assignment, we carried out a computational study at the time‐dependent DFT level to calculate the CD spectrum of Pd_12_ **L**_16_. Even though the large size of the Pd_12_ **L**_16_ complex and the computational demands connected with that led us to make certain approximations in the DFT calculations, the resulting spectrum using a fragment‐based approach is in very good agreement with the experimental one (Figure 4a, Supporting Information Section 3.1). The calculations also describe well the spectrum of Pd_3_(**L_d_**)_6_ (Figure 4a).

Therefore, a different structural organization of the same steroidal backbone in a supramolecular complex can lead to a different CD spectrum. Finding such a relationship between the CD signal and the structure represents a very useful tool in elucidating the connectivity of the ligands, i.e., 3,7‐Pd_C3‐C7_‐3,7 and the 24,24‐Pd_C24_‐24,24 corresponding to it, confirming the structural isomerism in Pd_6_ **L**_8_ and Pd_12_ **L**_16_. On that account, revealing the origin of the CD signals in these SCCs and comparing them with known supramolecular species (Supporting Information Section 3.1) and natural products, the CD method provides a potent alternative for the structural determination of chiral supramolecular species comparable to the often challenging single‐crystal X‐ray diffraction.

**Figure 5:**
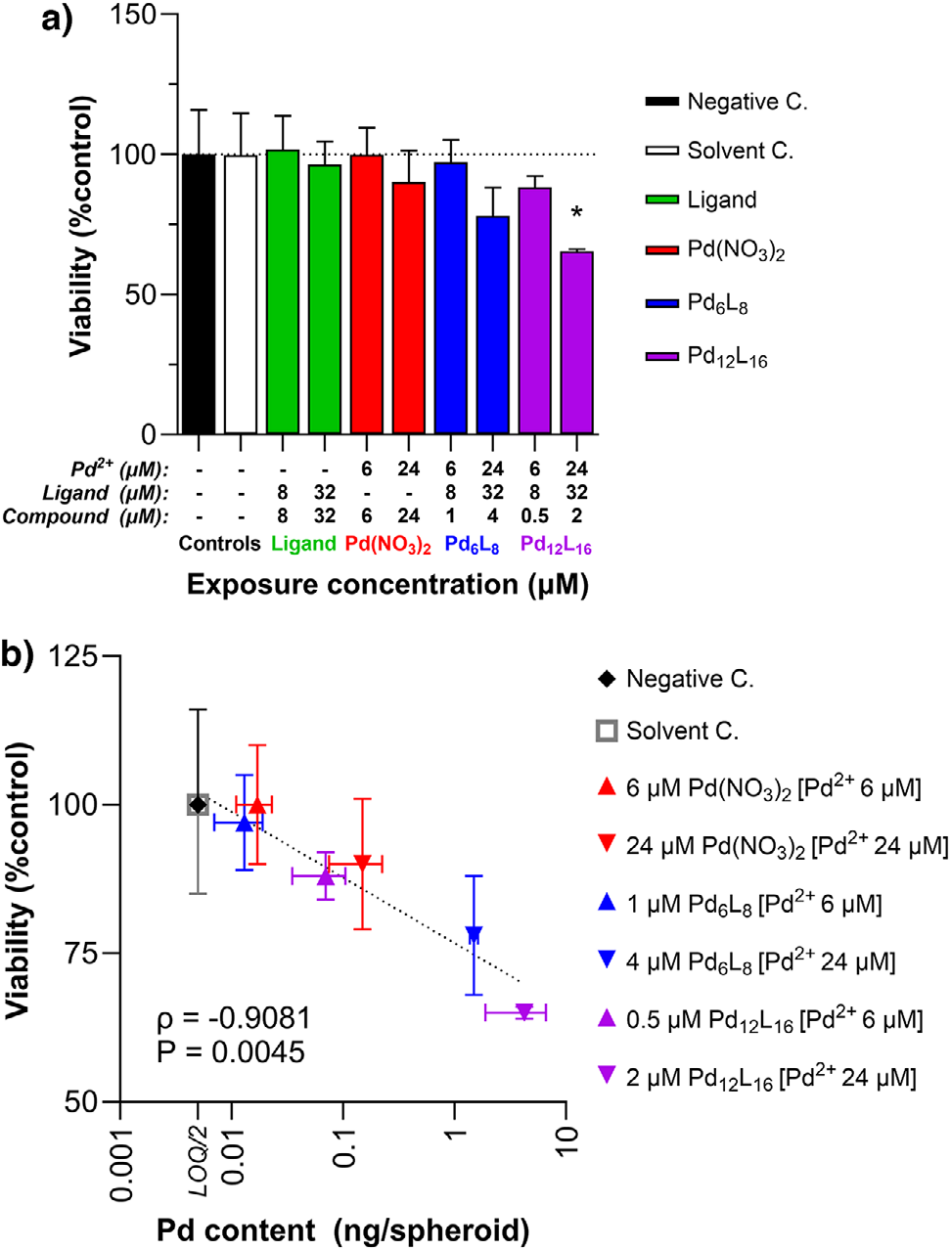
Toxicological studies of the SCCs. a) Concentration‐response of HepG2 spheroid viability (ATP content) after 8 days of exposure to Pd(NO_3_)_2_, **L**, Pd_6_ **L**_8_, and Pd_12_ **L**_16_. The asterisk (*) indicates a statistically significant (*P* < 0.05) difference from the solvent control. b) Relation of spheroid viability to palladium content measured in spheroids. *ρ* represents Spearman's rank correlation coefficient with a P value.

Moreover, CD spectroscopy may serve as a reliable and sensitive method to study the aqueous stability of Pd_6_ **L**_8_ and Pd_12_ **L**_16_ at the low concentrations relevant for biological studies (Figure S42). Consequently, considering the inherent transport ability of BAs within the enterohepatic circulation and the biological activity of Pd^2+^, we used spheroids of the human hepatoblastoma cell line HepG2 as a 3D in vitro model of the target liver tissue. The hepatospheroids were exposed to samples of Pd(NO_3_)_2_, **L**, Pd_6_ **L**_8_, and Pd_12_ **L**_16_. While **L** has shown negligible toxicity after an 8‐d exposure, the spheroid viability in response to an inorganic salt or SCCs decreased with increasing concentrations of Pd^2+^ ions. A comparison of equimolar Pd^2+^ concentrations (24 µM) of Pd(NO_3_)_2_, Pd_6_ **L**_8_, and Pd_12_ **L**_16_ shows spheroid viability reduced to 90%, 78%, and 65% of the control, respectively, as determined by the adenosine triphosphate (ATP) assay (Figure 5a) or by following on the spheroid size (Figure S57a, Table S9). At the end of the experiments, the spheroids were isolated, carefully and thoroughly washed, and submitted for studies by inductively coupled plasma mass spectrometry (ICP‐MS) to determine the palladium content. The palladium content in the spheroids displays a significant negative correlation with their viability (Figure 5b) and size (Figure S57b). The spheroids treated with 24 µM Pd(NO_3_)_2_ contained a low palladium content of 0.15 ng per spheroid, whereas 4 µM Pd_6_ **L**_8_ and 2 µM Pd_12_ **L**_16_ delivered 1.5 and 4.26 ng of bioactive palladium per spheroid, respectively (Figure 5b, Table S8). This corresponds to 0.023% cellular absorption efficiency at 24 µM Pd(NO_3_)_2_ (in total 638.5 ng of Pd(II) was applied), while the Pd_6_ **L**_8_ and the Pd_12_ **L**_16_ showed 0.2% and 0.7% uptake efficiency, respectively, at the equivalent palladium dose. The intraspheroid to extracellular concentration (IC:EC) ratios calculated across different species exhibited even more pronounced differences due to the reduced spheroid size in response to SCCs. At the high palladium dose, the IC:EC ratio was 248 for Pd_12_ **L**_16_ and 51 for Pd_6_ **L**_8_, representing a 56‐fold and 12‐fold increase, respectively, compared to the IC:EC ratio of 4.4 observed for Pd(NO_3_)_2_ (Table S9, Figure S57c). These results show that with larger and more charged SCCs, the affinity for uptake by the hepatospheroids, and consequently their toxic effects, increased (Figures 5b and S57b).

## Conclusion

The majority of the artificial metallo‐supramolecular complexes, unlike natural systems, are made of achiral, symmetric, and rigid ligands. In contrast, nature is ruled by the handedness of molecules, whereas the amino acids build and regulate living organisms through their left‐handedness, evolution assigned carbohydrates with right‐handed features. Control over the handedness of supramolecular coordination complexes (SCCs), by simply changing the connectivity of the ligand to a metal node, e.g., by introducing additional denticity to the ligand, is a very interesting feature which can lead to stimuli‐responsive interconversion of such cavity containing structures.

In this study, we provide a concept to design chiral, unsymmetric, flexible ligands employing natural bile acids in the construction of the “next generation” supramolecular coordination cages (SCCs). Implementing this concept we have introduced the first natural‐molecule‐based unsymmetric *tris*‐pyridyl ligand (**L**) derived from ursodeoxycholic bile acid. Because of the inherent flexibility, **L** can structurally adjust by expanding or contracting in coordination self‐assemblies with square‐planar Pd(II) into a Pd_6_ **L**_8_ octahedron or a giant Pd_12_ **L**_16_ cuboctahedron (5 nm in diameter). We refer to this controllable self‐assembly process as “flexibility‐aided orientational self‐sorting”. At the same time, Pd_12_ **L**_16_ represents the very first face‐directed self‐assembly of a tridentate ligand into the cuboctahedron, introducing a new group of M_12_L_16_ species.

So far, product mixtures resulting from rigid and symmetric ligands usually contain smaller species as the kinetically trapped ones,^[^ ^30^, ^43^ ^]^ whereas, in our case, it was found that using the unsymmetric and flexible ligand, the larger Pd_12_ **L**_16_ species represents the metastable form. Similarly in nature, while some large kinetic self‐assemblies might be less stable because of increased complexity and the potential for structural defects, others can be exceptionally stable because of robust intermolecular interactions and intricate design. Our example seems to be reaching the natural complexity.

The course of the reaction and the resulting product can be controlled by the choice of reaction conditions or later via interspecific transformations between Pd_6_ **L**_8_ and Pd_12_ **L**_16_. The process of structural switching has a special significance in view of the system adaptation to the environment representing a step forward towards artificial adaptive materials derived from natural products.

As for the structural elucidation, single‐crystal X‐ray diffraction is of paramount importance for SCCs. However, getting a quality single crystal is a challenging task in many cases, especially for flexible SCCs like ours. Accordingly, we employed various analytical techniques, with importance laid on CD spectroscopy in combination with molecular modelling, which confirm the orientational self‐sorting and structural isomerism of the SCCs into right‐handed quadruple (ΔΔΔΔ) or octuple (ΔΔΔΔΔΔΔΔ) helices. We envisage that CD spectroscopy, being a well‐established, sensitive, fast, and reliable tool for large chiral systems (e.g., DNA, proteins), can also be used to predict the structural organization of chiral metallo‐supramolecular complexes.

Because of their tailored composition, these water‐soluble SCCs show the enhanced cellular absorption and increased toxicity effect of palladium(II) cations when studied with spheroids of hepatoblastoma HepG2 cells. This anticancer effect of Pd^2+^ amplifies with increasing charge and size of the SCC. ICP‐MS analysis of hepatospheroids reveals 12‐fold (Pd_6_ **L**_8_) or 56‐fold (Pd_12_ **L**_16_) higher uptake of toxic Pd^2+^ than that of inorganic Pd(NO_3_)_2_. This interesting toxicological behaviour of bile‐acid‐based SCCs highlights the importance of developing natural molecule‐based coordination ligands and their supramolecular complexes for modern therapeutics.

The steroidal SCCs developed mimic natural hydrophobic cavities containing 80 (Pd_6_ **L**_8_) or a record 160 chiral centres (Pd_12_ **L**_16_) in their structures. Therefore, these SCCs, along with possible biomedical applications, are equally attractive for molecular recognition, enantioselective catalysis, and material science. We believe that the future of metallo‐supramolecular chemistry lies in the development and control of SCCs containing chiral nanocavities. Advancement in the field requires crucial understanding of their structural design and of their additional features such as substrate selective recognition, concerted interplay of reactive functionalities, or reversible dynamic structural changes for efficient substrate‐product turnover. The *flexibility‐aided orientational self‐sorting* we have introduced herein represents a novel approach leading to such supramolecular self‐assemblies at the interface of synthetic and biological fields. It will certainly be an interesting endeavour to continue in the future.

## Supporting Information

The authors have cited additional references within the Supporting Information.^[^ ^47^, ^48^, ^49^, ^50^, ^51^, ^52^, ^53^, ^54^ ^]^

## Conflict of Interests

The authors declare no conflict of interest.

## Supporting information



Supporting Information

## Data Availability

The data that support the findings of this study are available from the corresponding author upon reasonable request.
