# Supplementary material for: Flexibility‐Aided Orientational Self‐Sorting and Transformations of Bioactive Homochiral Cuboctahedron Pd12L16
Source: Angew Chem Int Ed Engl. 2025 Aug 10;64(37):e202513902. doi: 10.1002/anie.202513902 (PMC12416454; doi:10.1002/anie.202513902)
Supplement: Supplementary file 1 — Supporting Information [file ANIE-64-e202513902-s001.pdf]

Supporting Information
©Wiley-VCH 2024
69451 Weinheim, Germany

Flexibility-Aided Orientational Self-sorting and Transformations of Bioactive Homochiral Cuboctahedron $Pd_{12}L_{16}$

Subhasis Chattopadhyay, Robin Durník, Anniina Kiesilä, Elina Kalenius, Juha M. Linnanto, Pavel Babica, Jan Kuta, Radek Marek, Ondřej Jurček*

Abstract: Rational design and selective self-assembly of flexible and unsymmetric ligands into large coordination complexes is an eminent challenge in supramolecular coordination chemistry. Here we present coordination-driven self-assembly of natural ursodeoxycholic-bile-acid-derived unsymmetric *tris*-pyridyl ligand (**L**) resulting in selective and switchable formation of chiral stellated $Pd_6\mathbf{L}_8$ and $Pd_{12}\mathbf{L}_{16}$ cages. The cage selectivity originates in adaptivity and flexibility of ligand's arms bearing pyridyl moieties. The interspecific transformations can be controlled by changes in reaction conditions. Orientational self-sorting of **L** into a single constitutional isomer of each cage, i.e., homochiral quadruple and octuple right-handed helical species, was confirmed by combination of molecular modelling and circular dichroism. The cages, being derived from natural amphiphilic transport molecules, mediate higher cellular uptake and increase anticancer activity of bioactive palladium cations as determined in studies using *in vitro* 3D spheroids of human hepatic cells HepG2.

DOI: 10.1002/anie.2023XXXXX

Content

1 General Methods 3

1.1 NMR Spectroscopy 3

1.2 Electrospray Ionization Mass Spectrometry (ESI-MS) and Ion-Mobility Mass Spectrometry (IM-MS) 3

1.3 Circular Dichroism (CD) Spectroscopy 3

1.4 Fourier-Transform Infrared (FT-IR) Spectroscopy 4

1.5 Inductively Coupled Plasma Mass Spectrometry (ICP-MS) 4

1.6 Molecular Modelling 4

1.6.1 DFT calculations 4

1.7 Hepatotoxicity and Activity Assessment 5

1.7.1 HepG2 cell cultivation and spheroid preparation 5

1.7.2 Spheroid exposure 6

1.7.3 Statistical analysis 6

2 Synthesis and Characterization of Ligand and its Coordination Cages 7

2.1 Synthesis and Characterization of L 7

2.2 Complexation of L with Pd^{2+} Salts 14

2.2.1 $[Pd(CH_3CN)_4](BF_4)_2$ in $[D_6]$-DMSO (RM1) 14

2.2.2 Transformation reactions between Pd_6L_8 and $Pd_{12}L_{16}$ of RM1 18

2.2.3 $Pd(NO_3)_2{\cdot}2H_2O$ in $[D_6]$-DMSO (RM2 and RM2 3:2) 24

2.2.4 Variable temperature (VT) NMR spectroscopic study of RM2 27

2.2.5 $Pd(NO_3)_2{\cdot}2H_2O$ in 5% (v/v) $[D_3]$-ACN in $[D_6]$-DMSO solvent mixture (RM3) 29

2.2.6 Transformation reactions between Pd_6L_8 and $Pd_{12}L_{16}$ of RM2 and RM2 3:2 31

3 Computational Models 36

3.1 Helical Chirality 39

4 Monodentate Ligands and their Complexes with $Pd(NO_3)_2$ 40

4.1 Synthesis of L_{M24} 40

4.2 Synthesis of L_{M3} 43

4.3 Coordination of L_{M3} and L_{M24} with $Pd(NO_3)_2$ 46

5 Hepatotoxicity and Activity Evaluation 51

6 References 52

1 General Methods

1.1 NMR Spectroscopy

^{1}H, ^{13}C, DEPT-135, ^{1}H-^{13}C HSQC and ^{1}H-^{13}C HMBC NMR spectra of **L** were recorded using 5 mm dual inverse broad-band inversion probe at 700/500 MHz, 298.2 K and 10 mM concentration. ^{1}H, ^{13}C and ^{1}H-^{1}H NOESY NMR experiment for $Pd_{12}\mathbf{L}_{16}$ and $Pd_{6}\mathbf{L}_{8}$ cages were performed using TXO-cryoprobe. Residual internal solvent signal of [D_6]-DMSO was used as the reference for all spectra. ^{1}H-DOSY NMR experiments were conducted using 5 mm dual broad-band probe for diffusion measurements with Z-gradient strengths 23.1 mT/m at 700 MHz and 303.2 K. DiffSte pulse program was used to measure ^{1}H DOSY NMR spectra and Bruker's Dynamic Center software version 2.8.0.1 was used for data processing.

1.2 Electrospray Ionization Mass Spectrometry (ESI-MS) and Ion-Mobility Mass Spectrometry (IM-MS)

The DMSO stock solution of the reaction mixtures were diluted in MeCN. Samples were injected by direct infusion from syringe pump, using flowrate of 5 µl/min.

Mass spectrometry experiments were performed on an Agilent 6560 ESI-IM-QTOF mass spectrometer equipped with AJS ESI ion source. Nitrogen was used as dry-, sheath and nebulizer gas from nitrogen generator. A dry-gas temperature of 225 ºC, drying gas flow rate of 5 l/min, nebulizer pressure of 60 psi, sheath gas temperature of 225 ºC and sheath gas flow of 10 l/min were used. Capillary voltage of 3000 V and fragmentation voltage of 400 V were set as source parameters. The mass spectrometer was calibrated with an ES tuning mix from Agilent Technologies. Data was acquired with MassHunter Acquisition B.09.00 and analyzed using MassHunter Qualitative analysis B.08.00 as software packages from Agilent Technologies, USA.

In IM-MS experiments N_2 (6.0) was used as drift gas. Pressures for drift tube and high-pressure funnel were set as 3.95 and 3.80 Torr. The drift tube entrance and exit voltages were set as 1574 V and 224 V. A trap filling time of 5000 µs and trap release time of 350 µs were used. For multi-field measurements, the drift tube entrance voltage was varied from 1074 to 1674 V with 100 V increments. ES tuning mix was measured as a quality control sample to verify collision cross section (CCS) values.[1,2]

1.3 Circular Dichroism (CD) Spectroscopy

The stock solutions were 10 mM in [D_6]-DMSO as referred to ligand used, *i.e.*, **L**, $\mathbf{L_d}$, $Pd_{6}\mathbf{L}_{8}$, $Pd_{12}\mathbf{L}_{16}$, and $Pd_{3}(\mathbf{L_d})_{6}$. The CD spectra were measured on Jasco J-815 circular dichroism spectrometer. The 10 µM samples for measurement were prepared in a 10 mm cuvette by diluting the [D_6]-DMSO stock solutions in methanol or water. CD spectra were recorded in 225-340 nm range with 1 nm data pitch, 1 nm bandwidth, 2 s of data integration time and 100 nm/min scanning speed at 25 °C. Each data point was accumulated five times, and the average value is used for the spectrum. Solvent

spectra were used as the background and subtracted to produce the final spectra. Processing of data was performed using Jasco's spectra manager.

1.4 Fourier-Transform Infrared (FT-IR) Spectroscopy

FT-IR spectra were recorded of ligand as a powder using Bruker's ALPHA Compact FTIR Spectrometer and the data were processed using OPUS software.

1.5 Inductively Coupled Plasma Mass Spectrometry (ICP-MS)

Spheroids were hand-picked using Pasteur pipette, carefully and thoroughly washed with PBS buffer, and finally dispersed in 100 µL of PBS buffer. Subsequently, samples were transferred into 15 mL polypropylene tube and 0.3 mL of 65% nitric acid (Suprapur, Merck) and 0.15 mL of 36% hydrochloric acid (Suprapur, Merck) were added. Samples were mineralized for 4 hours at 70 °C in a Stuart SBH 200D/3 block heater and finally diluted to 5 mL with Milli-Q type 1 ultrapure water (Milli-Q Direct, Milliore). Determination of Pd in mineralized samples was performed by inductively coupled plasma mass spectrometry (Agilent 7700x ICP-MS, Agilent technologies). An ICP-MS instrument was equipped with MicroMist concentric nebulizer, Scott double-pass spray chamber, quartz torch with 2.5 mm i.d. injector and nickel sampling/skimmer cones. ICP-MS was operated at 1550 W input power, 8 mm sampling depth and 1.20 L/min flow of carrier gas (Ar). Octapole reaction system (3rd generation ORS3) operated at collision mode with helium flow 6 mL/min was used for suppression of spectral interferences. Limit of detection (LOD) was typically 10 pg/spheroid.

1.6 Molecular Modelling

The initial molecular modelling of $Pd_6\mathbf{L}_8$ and $Pd_{12}\mathbf{L}_{16}$ cage was performed using Spartan'20 (version 1.1.2). The next level molecular modeling was done by DFT calculations.

1.6.1 DFT calculations

The ground state geometries of studied structures were optimized at density functional CAM-B3LYP method using Gaussian 09 program.[3] SCRF solvent model for methanol was used in calculations. The initial geometries for the density functional calculations were taken from semi-empirical PM6 geometry optimization calculations. Time-dependent (TD) CAM-B3LYP method was used to calculate electronic transitions and CD intensities of the CAM-B3LYP optimized structures. LANL2DZ and 6-311G(d,p) basis sets on palladium and all other atoms, respectively, were used in the calculations. It appeared that calculations overestimate transition energies, thus linear correlation method was used for their estimation.[4] Experimental absorption band positions of the molecules and corresponding calculated transition energies gave the linear equation: $E_{estimated}$ [cm^{-1}] = 0.688 * $E_{calculated}$ [cm^{-1}] + 8839.5 cm^{-1} ($R^2 = 0.998$), needed in spectral simulations. In spectral simulations, inhomogeneous broadening of the spectral lines was modelled by using Gaussian distribution of random energy disorder. One spectral

simulation consisted of 10,000 iterations. The disorder (full width at half maximum) was 2 % of the value of the estimated transition energy. Gaussian line shape function of a width of 1800 cm^{-1} was used for all transitions.

Due to the large size of $Pd_{12}\mathbf{L}_{16}$ complex, its full geometry was optimized part by part. During optimization processes about half of the complex was free to move, while the remaining was fixed. This kind of local optimization process was continued several times until the whole complex was optimized at the DFT level and until atom positions of the complex were identical between adjacent optimization processes. The electronic transitions and CD intensities of the $Pd_{12}\mathbf{L}_{16}$ complex were calculated by using fragment-based approach.

1.7 Hepatotoxicity and Activity Assessment

1.7.1 HepG2 cell cultivation and spheroid preparation

The human hepatoblastoma cell line HepG2 (ATCC® HB-8065™) was provided by LGC Standards (Łomianki, Poland). The cells were grown in a low glucose (1 g/L) Minimum Essential Medium with phenol red (Thermo Fisher, Waltham, MA, Gibco, 61100), supplemented with 1% (v/v) Minimum Essential Medium Non-Essential Amino Acids (Thermo Fisher, Gibco 11140050), 1 mM sodium pyruvate (Thermo Fisher), and sodium bicarbonate (1.5 g/L, Sigma-Aldrich, Prague, Czech Republic). The medium was sterile-filtered (0.2 µm PES filter, TPP, Trasadingen, Switzerland), and it was also supplemented with 10% of fetal bovine serum (Biosera, Nuaillé, France, 1001/500). The cells were routinely cultured in monolayer cultures in 25 cm^2 cell culture flasks (TPP) at 37 °C in 5% CO_2 and passaged twice per week before reaching 80% confluency. Before being transferred to a new flask for further propagation, the cells were detached using trypsin/EDTA (Thermo Fisher), re-suspended and diluted three-fold with fresh culture medium. For the spheroid assays, the concentration of the cell suspension was determined using an automated cell counter (Nexcelom Bioscience, Lawrence, MA).[5]
The inner (6×10) wells of black 96-well plates (Greiner Bio One, Kremsmünster, Austria, CELLSTAR®, 655090) were pre-coated with 50 µL of melted sterile 1.5% (w/v) agarose (Sigma-Aldrich, A9539) in 0.9% NaCl (w/v). Additional three peripheral wells were also pre-coated with agarose to function as a blank in the selected assays. The remaining peripheral wells were filled with 250 µL of sterile phosphate-buffered saline (PBS). After solidification of the agarose, 1000 cells per well were seeded in 200 µL of the culture medium. The assay blank wells were filled with 200 µL of culture medium only. Agarose prevents attachment of the cells to the microplate bottom and the concave shape of the agarose coating causes aggregation of HepG2 cells in the centre of the well. This leads to formation and 3D growth of a single spheroid per well. The plates were cultured at 37 °C in 5% CO_2 for 4 days prior the experimental treatments.[5]

1.7.2 Spheroid exposure

500X stock solutions of $Pd(NO_3)_2.2H_2O$ (Sigma-Aldrich, 76070, dissolved in DMSO), ligand **L** (dissolved in DMSO), Pd_6**L_8** (dissolved in 95:5 deuterated DMSO:MeCN, MeCN was purchased from Eurisotop, 2206-26-0), and Pd_{12}**L_{16}** (dissolved in deuterated DMSO, purchased from Eurisotop, Saint-Aubin, France, 2206-27-1) were aseptically diluted with complete sterile culture medium to produce exposure medium at a concentration of the sample being 2.5-times the final desired concentration in the assay. This stock solution was then added to the 4-day-old spheroid cultures to achieve the desired final (1X) nominal concentrations, as outlined in Table S6: 8 and 32 μM for ligand **L**, 6 and 24 μM for $Pd(NO_3)_2$, 1 and 4 μM for Pd_6**L_8** (RM2 3:2), and 0.5 and 2 μM for Pd_{12}**L_{16}** (RM2). This allowed the nominal Pd concentration in each well to correspond to either 0 μM (negative control, solvent control, ligand), 6 μM or 24 μM (for $Pd(NO_3)_2$, Pd_6**L_8**, Pd_{12}**L_{16}**). Solvent control was prepared using the corresponding solvents, with the final concentration 0.2% of the organic solvent. The exposure time was 192 h (8 days, with no further medium change conducted during the exposure). The spheroid size (planar area, μm²) was analysed from their microphotographs obtained with BioTek® Cytation 5 imaging reader (using 4x objective) and Gen5 software (Agilent Technologies, Winooski, VT). The spheroid planar area (μm²) obtained from image analysis was converted to the volume of a perfect sphere with an equivalent planar area. The spheroid volumes were normalized to the average volume of non-treated spheroids, i.e., negative control, and expressed as % control.

After imaging, the spheroids were either collected for ICP-MS analysis (Section 1.5) or used for viability assessment by measuring the ATP content, which is proportional to the number of viable and metabolically active cells in the spheroids. Both spheroid and assay blank wells (i.e., agarose with culture medium without cells) were rinsed twice with PBS by removing and replacing 100 μL of solution in each step. Then, 100 μL of the wash solution was removed from each well, and 100 μL of pre-warmed (37 °C) CellTiter-Glo® 3D Reagent (Promega, Madison, WI, #G9682) was added to each well. The microplate was vigorously mixed using a microplate shaker for 5 minutes and incubated for another 25 minutes at room temperature in the dark. Luminescence signal was recorded using Cytation 5 with an integration time of 1 second per well. Luminescence from the blank wells was subtracted from the spheroid readouts, which were then normalized to the blank-subtracted average signal from the negative control wells and expressed as % control. The experiments were conducted with six replicates: three for Pd analysis and three for viability assays.

1.7.3 Statistical analysis

Since there were no differences between the solvent control of DMSO and the solvent control of 95:5 DMSO:ACN, values from both controls were combined for the final analysis and presentation. Statistical significance of differences observed between solvent control and different treatments were evaluated using One Way ANOVA, eventually followed by post-hoc Dunnett's test to identify treatments significantly different from the solvent control using SigmaPlot Systat software (Grafiti

LLC, Palo Alto, CA). Data in graphs represent average values +/- standard deviations from at least 3 replicates.

2 Synthesis and Characterization of Ligand and its Coordination Cages

2.1 Synthesis and Characterization of **L**

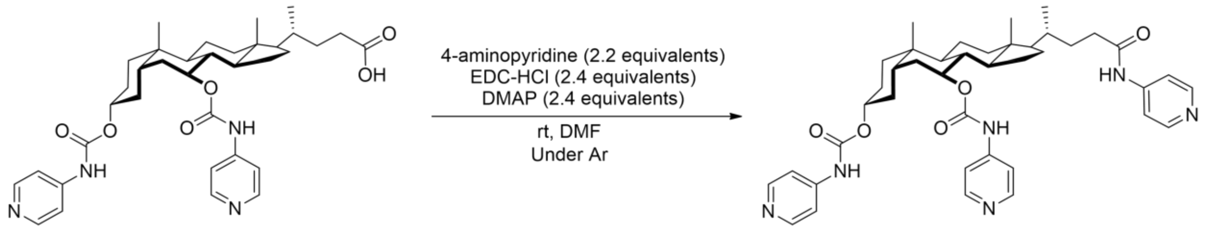

$\mathbf{L}_{SM}$ **L**

Scheme S1. Final step of synthesis of ligand **L**.

The starting material ($\mathbf{L}_{SM}$) was synthesized from UDCA in three steps following previous reports.[6-8] To a solution of $\mathbf{L}_{SM}$ (200 mg, 1 equivalent) in *N,N*-dimethylformamide (5 mL) 4-aminopyridine (2.2 equivalents), 1-ethyl-3-(3′-dimethylaminopropyl) carbodiimide hydrochloride (EDC·HCl, 2.4 equivalents) and 4-(dimethylamino)pyridine (DMAP, 2.4 equivalents) were added in sequence. The reaction mixture was stirred overnight at room temperature under argon atmosphere. The product was purified using preparative thin layer chromatography (TLC) with ethyl acetate:methanol:water 25:2:1 as the mobile phase. The product was extracted from silica using ethanol resulting in a white solid (141 mg) in 63 % yield.

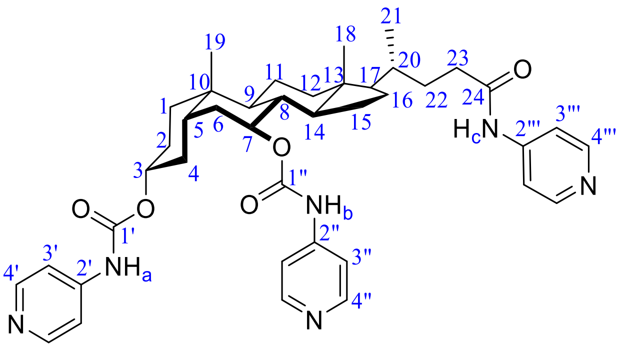

Figure S1. Chemical structure and carbon numbering of **L**.

Analytical data of **L**:
^{1}H NMR (700 MHz, [D$_6$]-DMSO, 298.2 K): δ = 10.23 (s, 1H, -NH$_c$-), 10.05 (s, 1H, -NH$_b$-), 9.98 (s, 1H, -NH$_a$-), 8.35 (m, 6H, H-4', H-4" and H-4"'), 7.51 (m, 2H, H-3"'), 7.43 (m, 4H, H-3' and H-3"), 4.71 (m, 1H, H-7), 4.60 (m, 1H, H-3), 2.37 and 2.24 (m, 2H, H-23), 2.01-1.03 (overlapping, 24H, steroid skeleton), 0.96 (s, 3H, H-19), 0.92 (d, *J* = 6.6 Hz, 3H, H-21), 0.66 (s, 3H, H-18) ppm.
^{13}C NMR (175 MHz, [D$_6$]-DMSO, 298.2 K): δ = 172.96 (C24), 152.79 and 152.73 (C1' and C1"), 150.33 and 150.23 (C4', C4" and C4"'), 146.19 (C2' and C2"), 145.82 (C2"'),

113.06 (C3'''), 112.37 (C3''), 112.19 (C3'), 74.20 (C3), 73.85 (C7), 54.58 (C14 and C17), 43.18 (C13), 41.31 (C5), 40.02 (C8), 39.40 (C12), 38.88 (C9), 34.80 (C20), 33.89 (C1), 33.59 (C10), 33.54 (C23), 33.10 (C6), 32.78 (C4), 31.00 (C22), 27.98 (C16), 26.28 (C2), 25.29 (C15), 22.86 (C19), 20.88 (C11), 18.43 (C21), 11.89 (C18) ppm.

HRMS (*m/z*) for $[M+H]^+ = [C_{41}H_{53}N_6O_5]^+$: theoretical mass 709.4072, found experimental mass 709.4076, mass accuracy 0.6 ppm.

FT-IR (cm^{-1}): 3750-2250 (br), 2937, 2870, 1732, 1717, 1701, 1654, 1636, 1589 (s), 1511 (s), 1456, 1415, 1385, 1332 (s), 1295, 1242 (s), 1224 (s), 1203 (s), 1122, 1095, 1073, 1052 (s), 997 (s), 959, 910, 877, 855, 824 (s), 771, 687, 665, 644, 606, 583, 571, 532 (s), 473, 465, 443, 420.

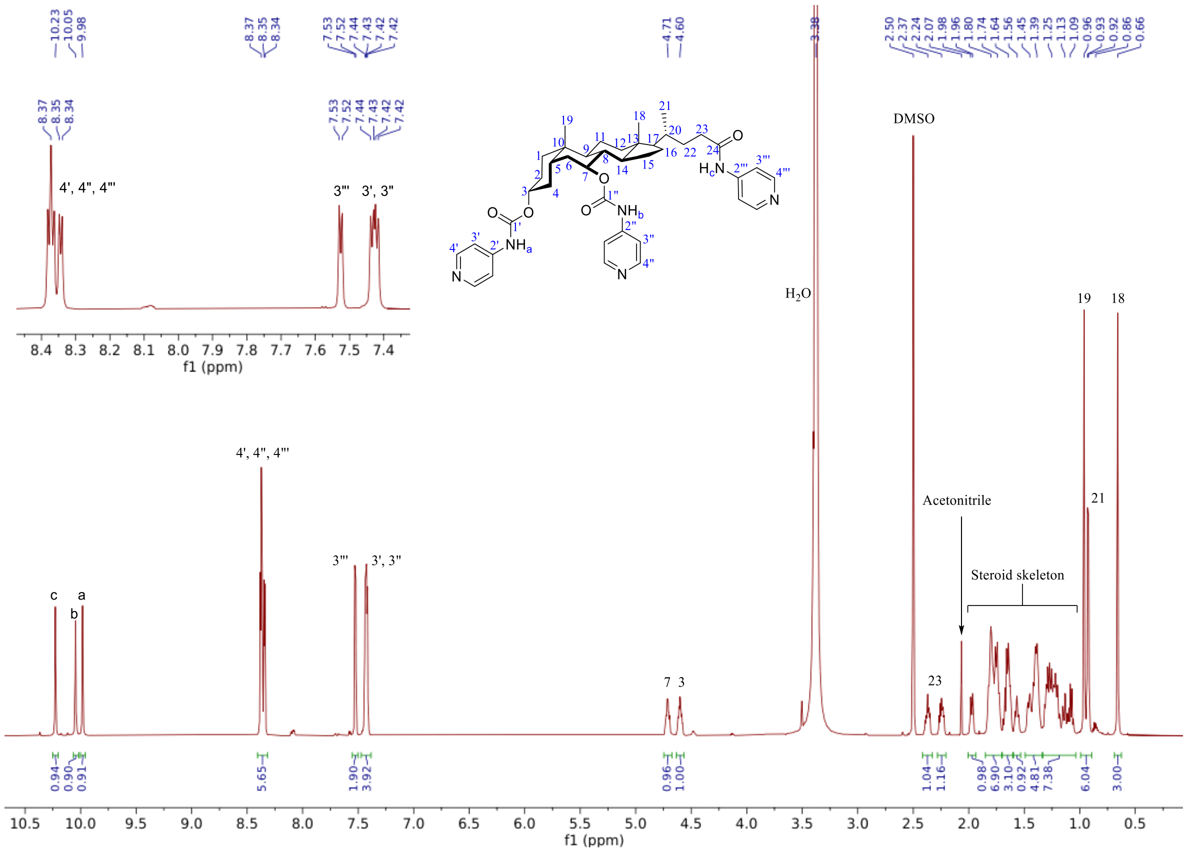

Figure S2. ^{1}H NMR spectrum of **L** measured in [D$_6$]-DMSO at 700 MHz and 298.2 K.

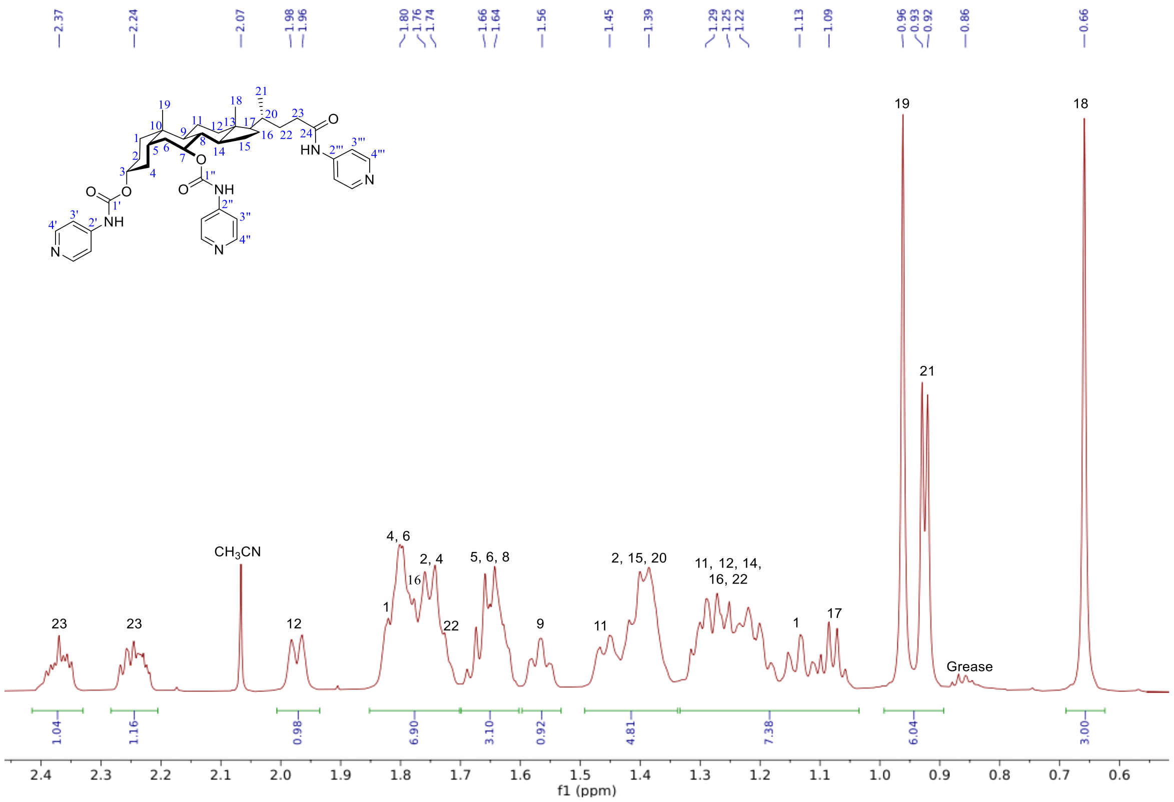

Figure S3. ^{1}H NMR spectrum of **L** steroid skeleton measured in [D6]-DMSO at 700 MHz and 298.2 K.

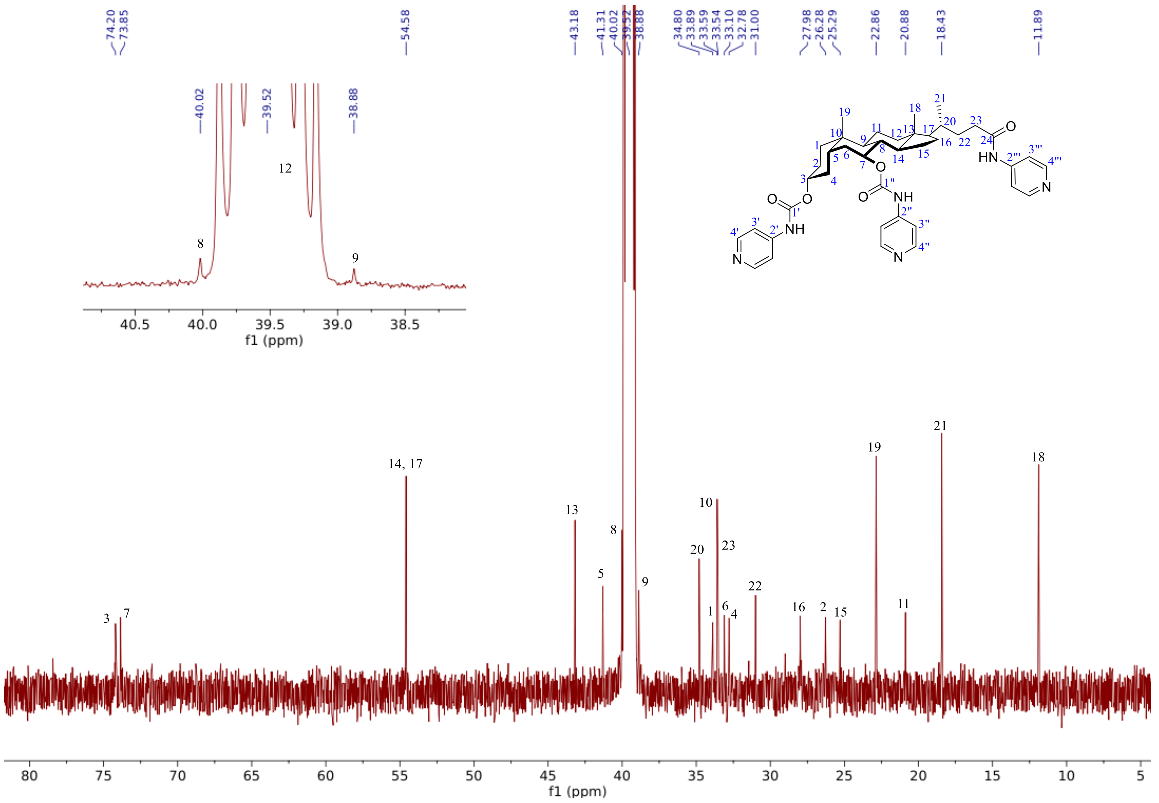

Figure S4. ^{13}C NMR spectrum of **L** (aliphatic region) measured in [D6]-DMSO at 175 MHz and 298.2 K.

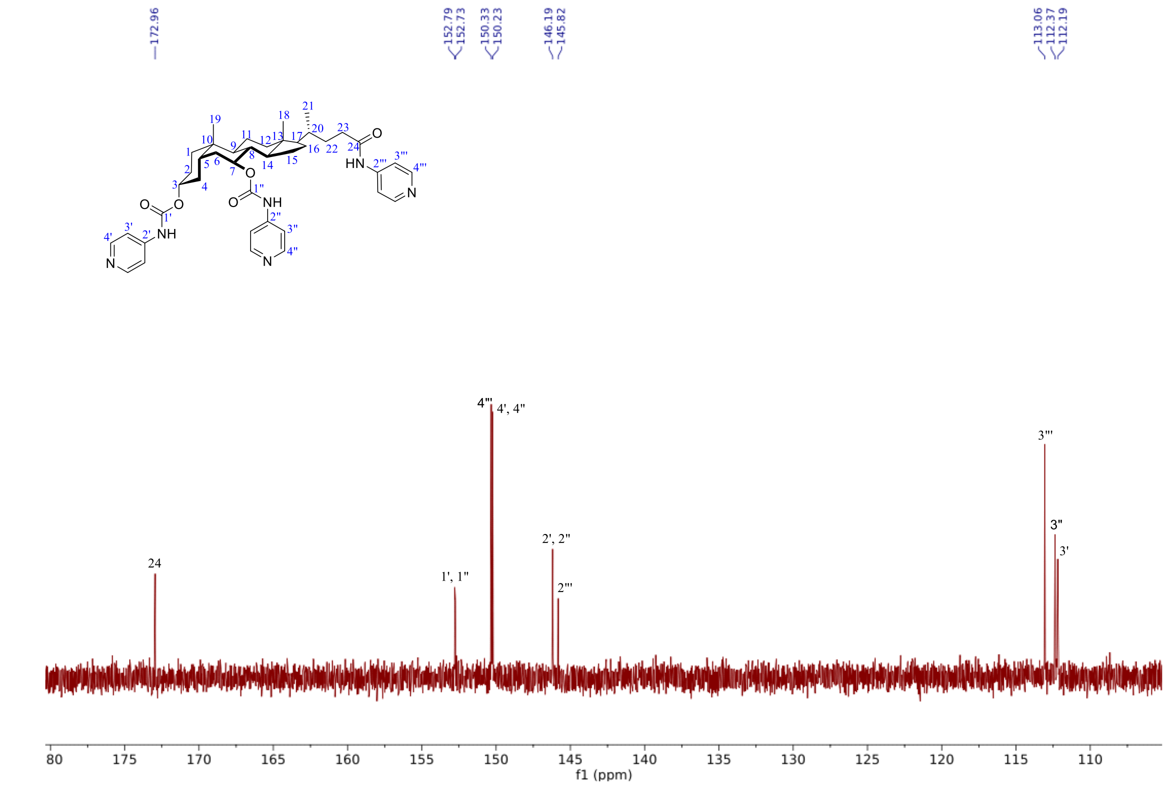

Figure S5. ^{13}C NMR spectrum of **L** (aromatic and carbonyl region) measured in [D$_6$]-DMSO at 175 MHz and 298.2 K.

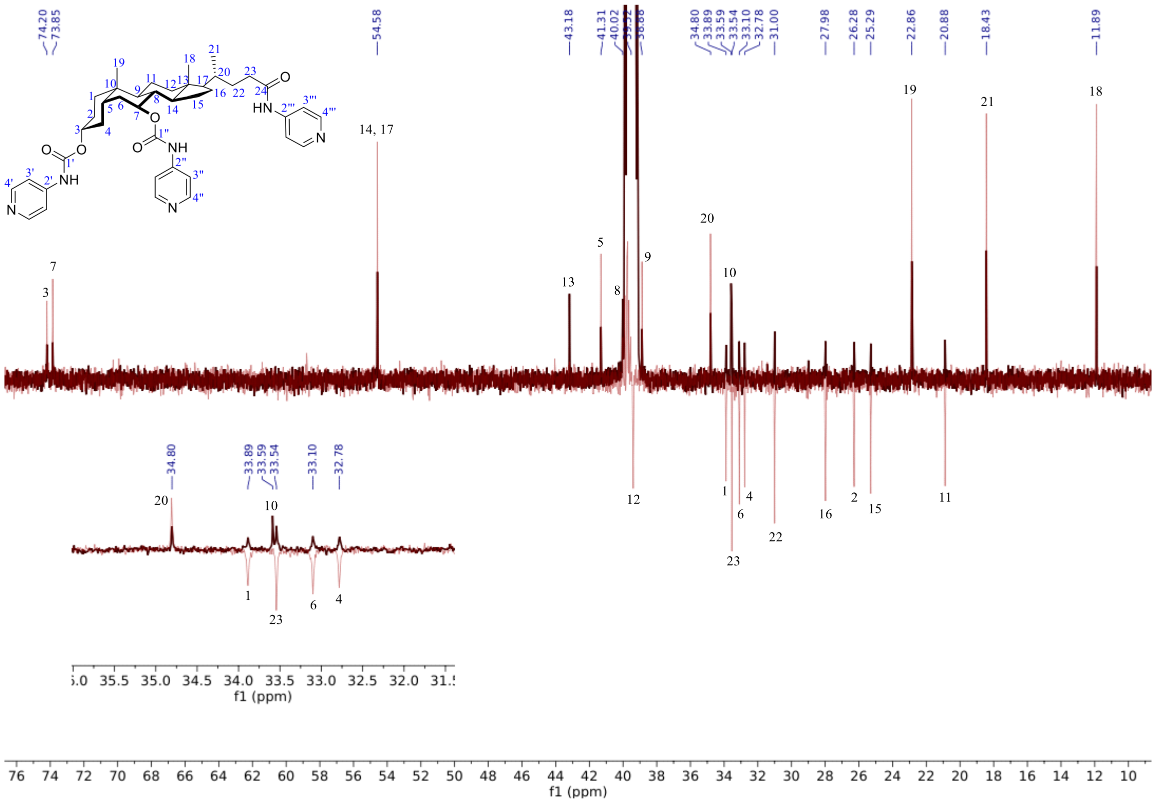

Figure S6. Comparison of ^{13}C and DEPT-135 NMR spectrum (aliphatic region) of **L** measured in [D$_6$]-DMSO at 175 MHz at 298.2 K.

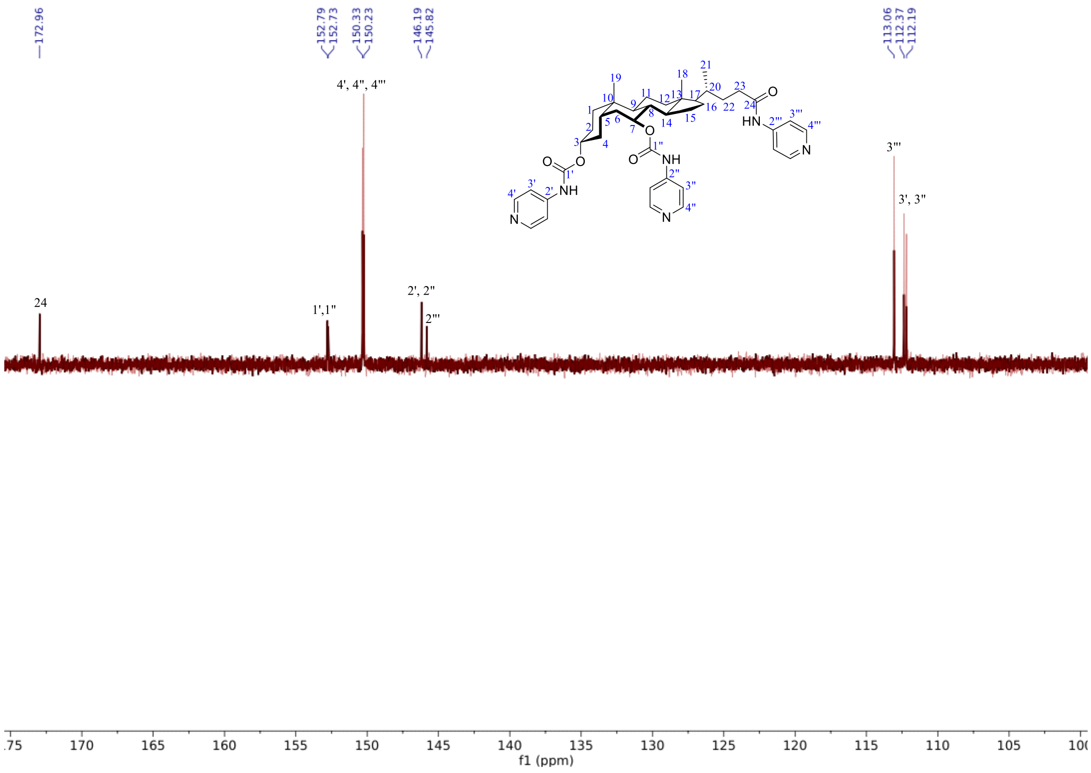

Figure S7. Comparison of ^{13}C and DEPT-135 NMR spectrum (aromatic and carbonyl region) of **L** measured in [D6]-DMSO at 175 MHz at 298.2 K.

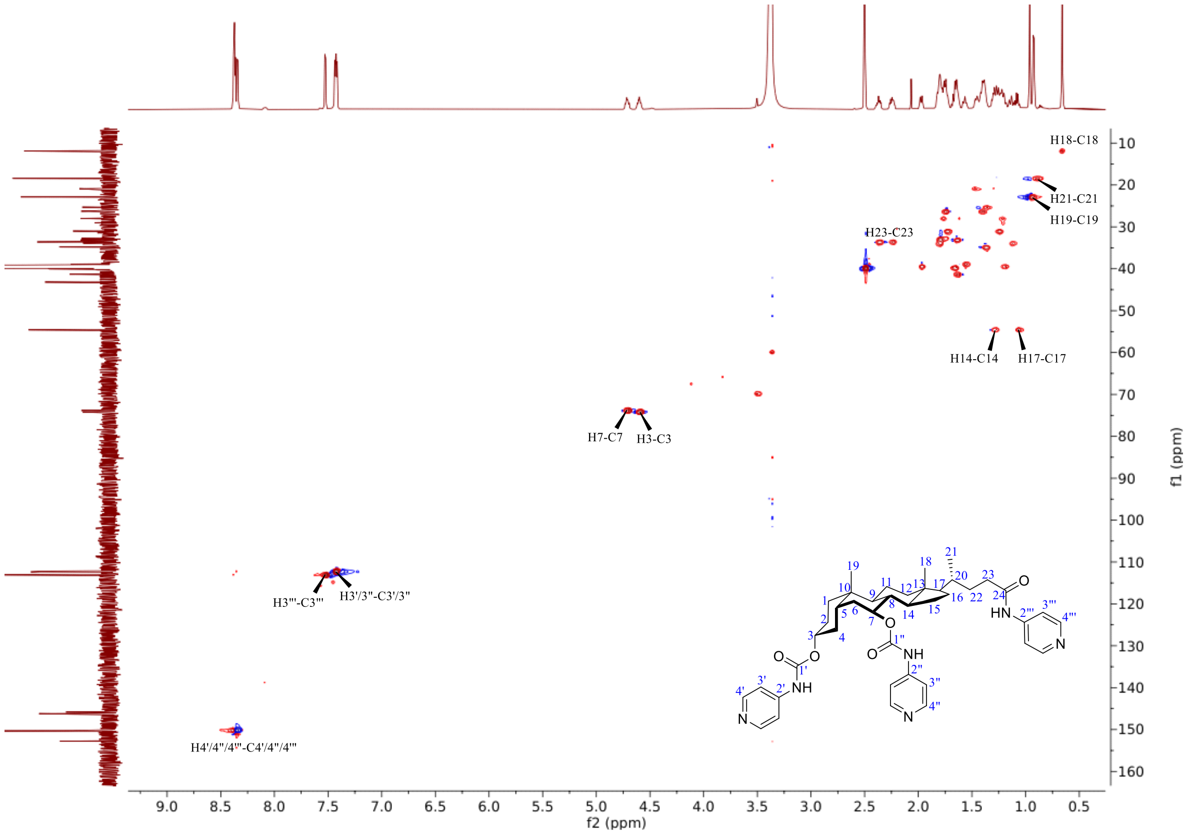

Figure S8. ^{1}H-^{13}C HSQC NMR spectrum of **L** measured in [D6]-DMSO at 700 MHz and 298.2 K.

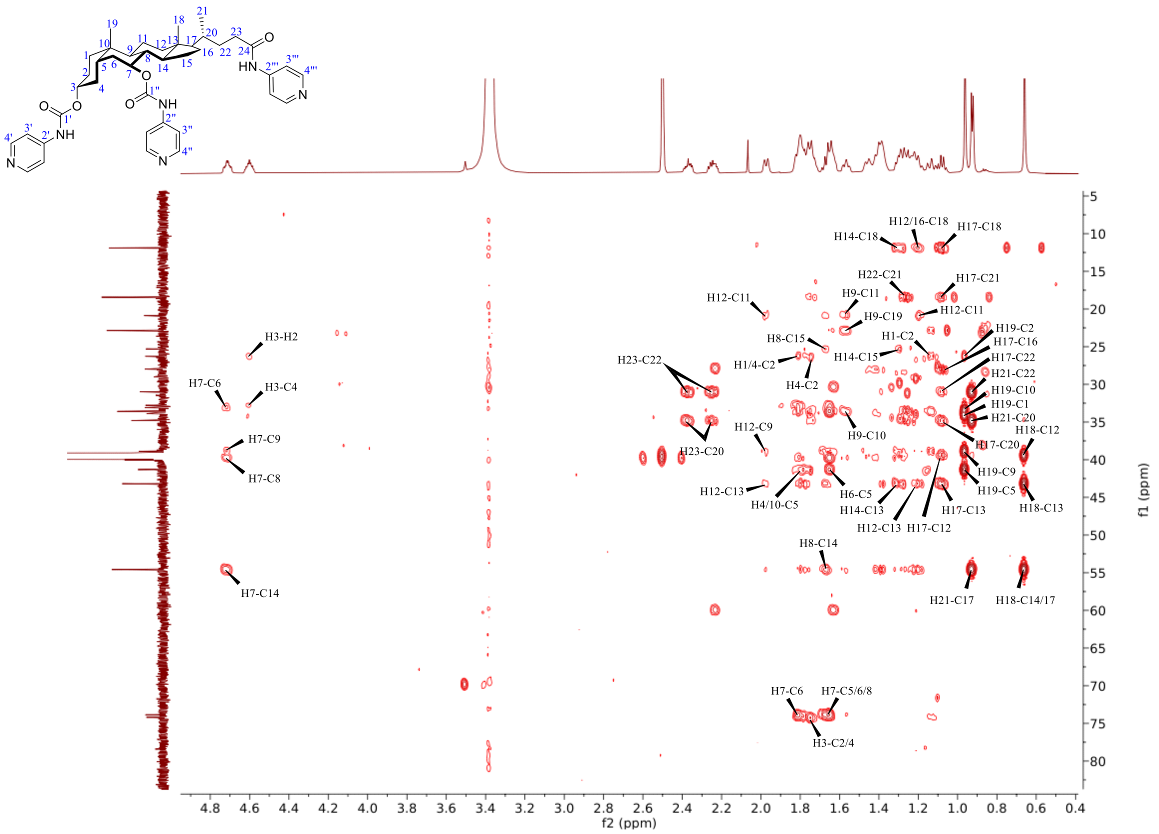

Figure S9. ^{1}H-^{13}C HMBC NMR spectrum (aliphatic region) of **L** measured in [D6]-DMSO at 700 MHz and 298.2 K.

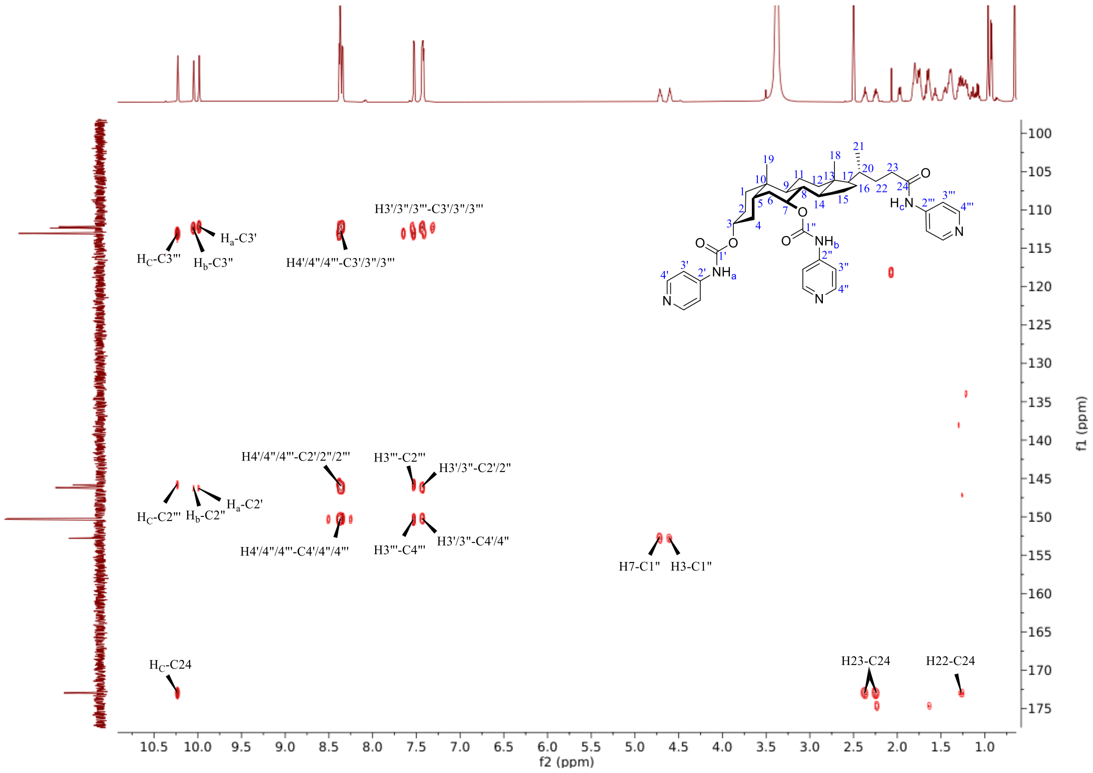

Figure S10. ^{1}H-^{13}C HMBC NMR spectrum (aromatic and carbonyl region) of **L** measured in [D6]-DMSO at 700 MHz and 298.2 K.

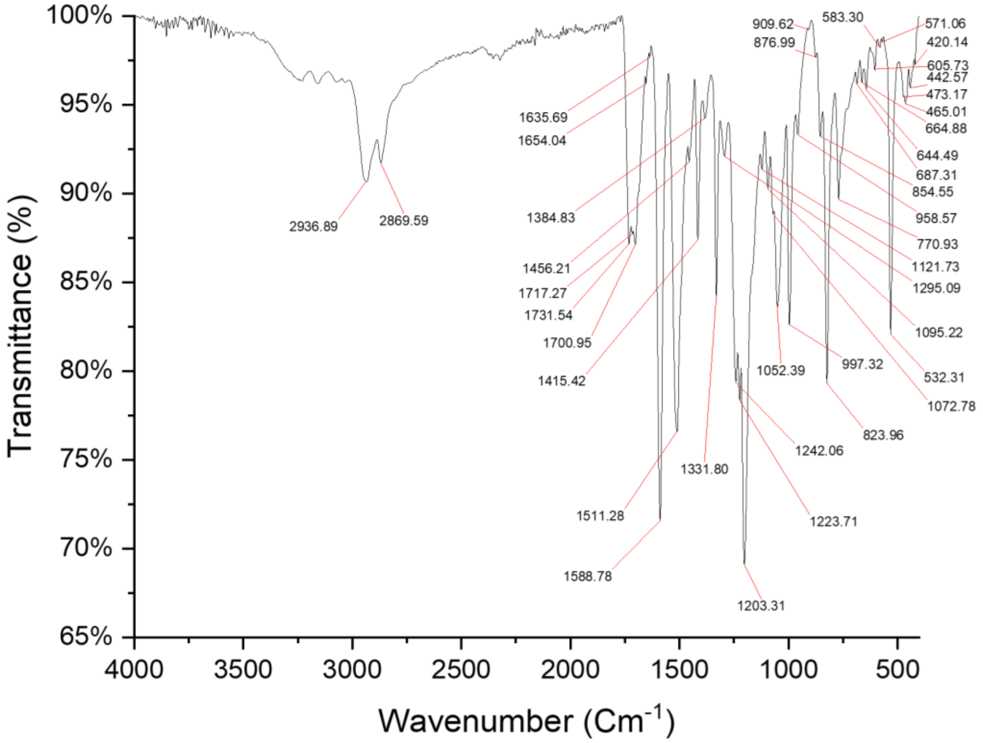

Figure S11. FT-IR spectrum of **L**.

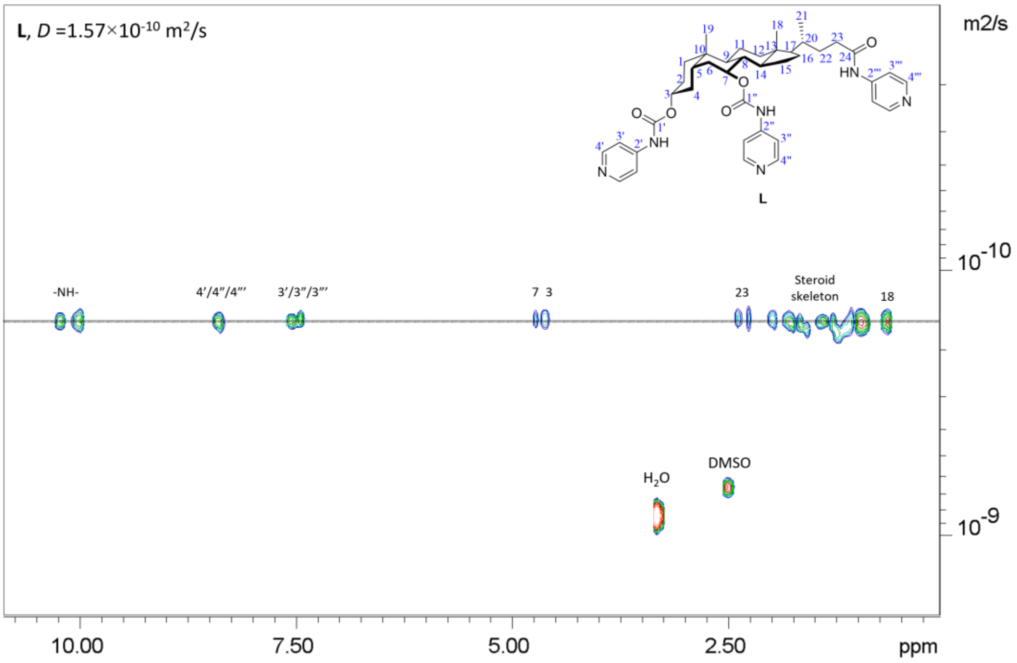

Figure S12. ^{1}H DOSY NMR of L in [D$_6$]-DMSO at 303.2 K and 700 MHz.

2.2 Complexation of **L** with Pd^{2+} Salts

2.2.1 [Pd(CH₃CN)₄](BF₄)₂ in [D₆]-DMSO (RM1)

Complexation reaction was performed with 10 mM [D_6]-DMSO (500 µL) solution of **L** (3.5 mg) which was mixed with [Pd(CH_3CN)$_4$](BF_4)$_2$ (M:**L** 3:4, 1.7 mg) (reaction mixture 1 = RM1). The resulting solution was heated at 70 °C for 1 hour yielding a mixture of Pd_6**L**$_8$ and Pd_{12}**L**$_{16}$.

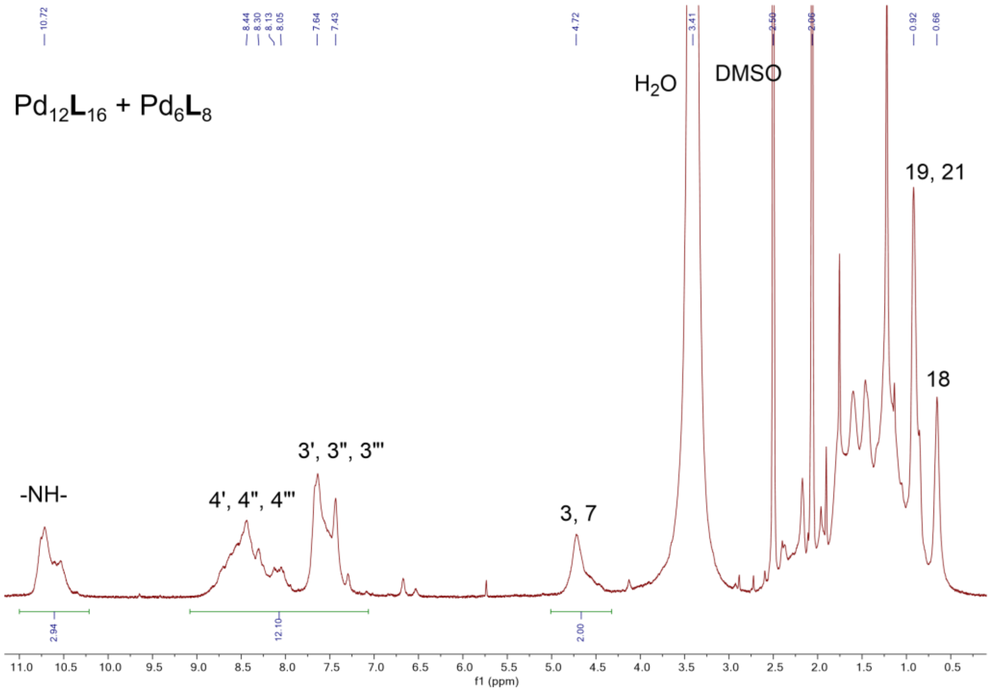

Figure S13. ^{1}H NMR spectrum of complexation reaction of **L** with [Pd(CH_3CN)$_4$](BF_4)$_2$ in [D_6]-DMSO (RM1) at 298.2 K and 700 MHz.

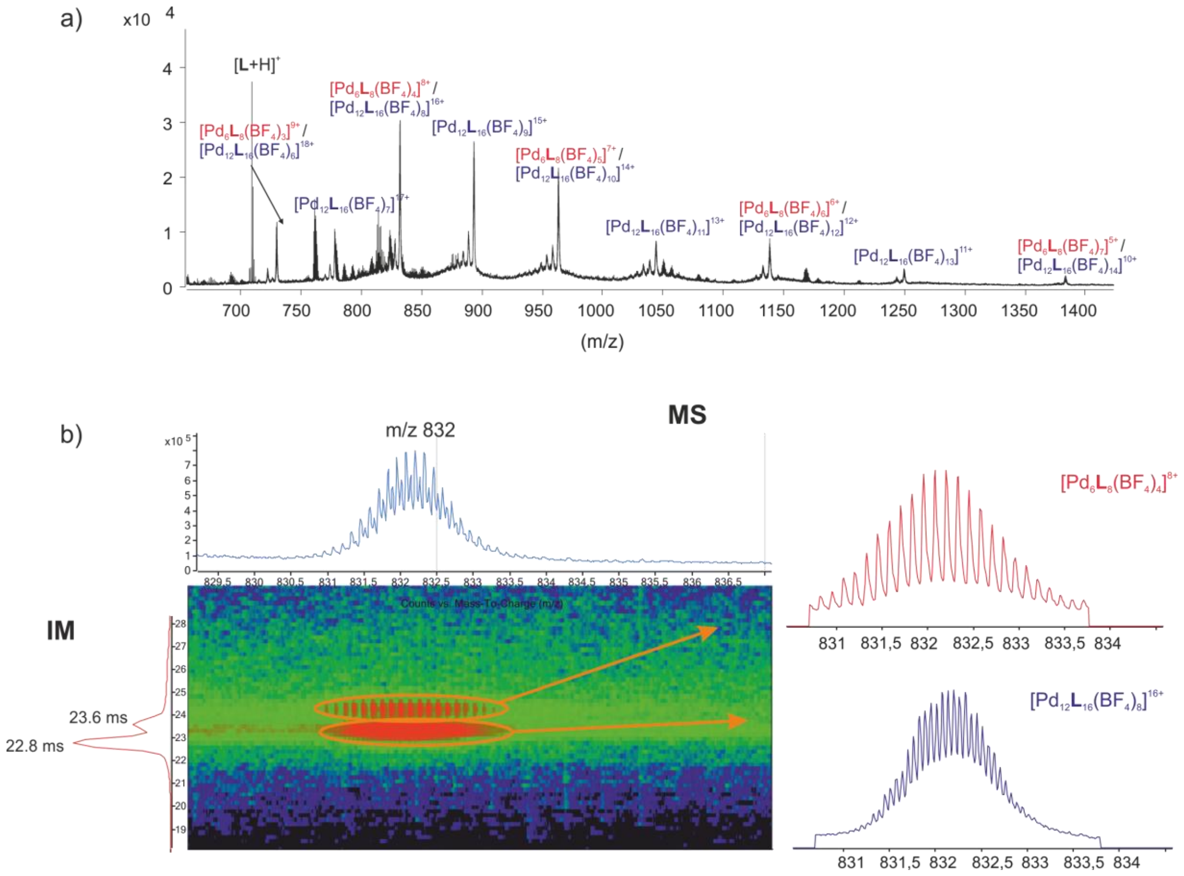

Figure S14. a) ESI-QTOF mass spectrum from sample containing $[Pd(CH_3CN)_4](BF_4)_2$ and **L** with M:L 3:4 ratio (RM1). b) IM-MS data for base peak at m/z 832, showing 2D data (heat plot) for MS (top, light blue) and ion mobility (left, red ion mobilogram). Ion mobilogram shows two species at drift times 23.6 ms and 22.8 ms, for which extracted mass spectra corresponding to $Pd_{12}L_{16}$ (blue) and Pd_6L_8 (red) are shown on right side.

ESI-MS spectra often show formation of unspecific dimers, but there are several observations, which imply that Pd_{12}**L**$_{16}$ is the main product and not a dimer of the smaller Pd_6**L**$_8$ cage: 1) often unspecific dimers are not observed as main products having the highest intensity, 2) dimerization is often concentration dependent process which was not observed here, 3) increase in CCS from Pd_6**L**$_8$ to Pd_{12}**L**$_{16}$ is less than 40%, and 4) the most importantly, Pd_{12}**L**$_{16}$ is also observed at uneven charge states from +9 to +17, which would not be possible for unspecific dimers.

Ion	z	m/z$_{exp}$	m/z$_{theor}$	M$_W$ (Da)	Mass Accuracy (mDa)	DT (ms)	^{DT}CCS$_{N2}$ (Å^2)	Diameter (Å)	Height of drift peak
$[Pd_6\mathbf{L}_8(BF_4)]^{11+}$	11	581.4261	581.4212	6395.6332	-4.9	18.46	1463.8	43	932635
$[Pd_6\mathbf{L}_8(BF_4)_2]^{10+}$	10	648.267	648.2638	6482.638	-3.2	19.65	1420.1	43	3085113
$[Pd_6\mathbf{L}_8(BF_4)_3]^{9+}$	9	729.9616	729.9602	6569.6418	-1.4	21.33	1389.1	42	6222694
$[Pd_{12}\mathbf{L}_{16}(BF_4)_6]^{18+}$	18	729.9627	729.9603	13139.2854	-2.4	20.65	2676	58	1840883
$[Pd_{12}\mathbf{L}_{16}(BF_4)_7]^{17+}$	17	778.0138	778.017	13226	3.2	21.64	2663.1	58	6794673
$[Pd_6\mathbf{L}_8(BF_4)_4]^{8+}$	8	832.0822	832.0808	6656.6576	-1.4	23.59	1448.2	43	5360269
$[Pd_{12}\mathbf{L}_{16}(BF_4)_8]^{16+}$	16	832.0781	832.0808	13313.2496	2.7	22.78	2639.3	58	15821212
$[Pd_{12}\mathbf{L}_{16}(BF_4)_9]^{15+}$	15	893.3502	893.3532	13400.253	3	24.12	2623.8	58	16326231
$[Pd_{12}\mathbf{L}_{16}(BF_4)_{10}]^{14+}$	14	963.3763	963.3787	13487.2682	2.4	25.63	2599.9	58	10236437
$[Pd_6\mathbf{L}_8(BF_4)_5]^{7+}$	7	963.3775	963.3787	6743.6425	1.2	24.58	na	-	1232463
$[Pd_6\mathbf{L}_8(BF_4)_5]^{7+}$	7	963.3798	963.3787	6743.6586	-1.1	23.66	1301.3	41	1879702
$[Pd_{12}\mathbf{L}_{16}(BF_4)_{11}]^{13+}$	13	1044.0982	1044.1004	13573.2766	2.2	27.31	2565.4	57	2346584
$[Pd_{12}\mathbf{L}_{16}(BF_4)_{11}]^{13+}$	13	1044.0983	1044.1004	13573.2779	2.1	23.61	2212.3	53	2068908
$[Pd_{12}\mathbf{L}_{16}(BF_4)_{12}]^{12+}$	12	1138.3548	1138.3592	13660.2576	4.4	24.96	2148.4	52	3622403
$[Pd_6\mathbf{L}_8(BF_4)_6]^{6+}$	6	1138.2777	1138.2758	6829.6662	-1.9	25.99	1146.1	38	644356
$[Pd_{12}\mathbf{L}_{16}(BF_4)_{13}]^{11+}$	11	1249.753	1249.7558	13747.283	2.8	26.01	2066.4	51	1039800
$[Pd_6\mathbf{L}_8(BF_4)_7]^{5+}$	5	1383.3332	1383.3318	6916.666	-1.4	30.29	1086.5	37	132478
$[Pd_{12}\mathbf{L}_{16}(BF_4)_{14}]^{10+}$	10	1383.4279	1383.4318	13834.279	3.9	27.44	1975.9	50	543690
$[Pd_{12}\mathbf{L}_{16}(BF_4)_{15}]^{9+}$	9	1546.8074	1546.8136	13921.2666	6.2	29.13	1887.5	49	157729
$[Pd_6\mathbf{L}_8(BF_4)_8]^{4+}$	4	1750.9161	1750.9158	7003.6644	-0.3	36.78	1105.4	38	41417
$[Pd_{12}\mathbf{L}_{16}(BF_4)_{16}]^{8+}$	8	1750.9088	1750.9158	14007.2704	7	30.65	1776.8	48	99415

Table S1. Ions observed in IM-MS data for $Pd_{12}L_{16}$ and Pd_6L_8 mixture (RM1: M:**L** = 3:4) obtained using $[Pd(CH_3CN)_4](BF_4)_2$. Ion interpretation, charge states (z), *m/z* values, mass accuracies, drift times (DT), collision cross sections, diameter calculated for ions presented, and height of drift peak.

Ion	z	m/z_{exp}	m/z_{theor}	M_W (Da)	Mass accuracy (mDa)	DT (ms)	Height of drift peak
$[\mathbf{L}_8+Pd_6(BF_4)]^{11+}$	11	581.4274	581.4212	6395.6332	-6.2	18.6	2022220
$[\mathbf{L}_8+Pd_6(BF_4)_2]^{10+}$	10	648.2671	648.2638	6482.638	-3.3	19.81	9227667
$[\mathbf{L}_8+Pd_6(BF_4)_3]^{9+}$	9	729.9641	729.9602	6569.6418	-3.9	21.62	17707284
$[\mathbf{L}_{16}+Pd_{12}(BF_4)_7]^{17+}$	17	778.1336	778.017	13226	-116.6	21.84	1957969
$[\mathbf{L}_8+Pd_6(BF_4)_4]^{8+}$	8	832.085	832.0808	6656.68	-4.2	23.75	11736865
$[\mathbf{L}_{16}+Pd_{12}(BF_4)_8]^{16+}$	16	832.0836	832.0808	13313.3376	-2.8	22.93	2860790
$[\mathbf{L}_{16}+Pd_{12}(BF_4)_9]^{15+}$	15	893.3496	893.3532	13400.244	3.6	24.17	3168878
$[\mathbf{L}_{16}+Pd_{12}(BF_4)_{10}]^{14+}$	14	963.3797	963.3787	13487.3158	-1	25.62	2483043
$[\mathbf{L}_8+Pd_6(BF_4)_5]^{7+}$	7	963.3861	963.3787	6743.7027	-7.4	24.63	1337883
$[\mathbf{L}_8+Pd_6(BF_4)_5]^{7+}$	7	963.3853	963.3787	6743.6971	-6.6	23.79	2474778
$[\mathbf{L}_{16}+Pd_{12}(BF_4)_{11}]^{13+}$	13	1044.1059	1044.1004	13573.3767	-5.5	27.23	771147
$[\mathbf{L}_{16}+Pd_{12}(BF_4)_{11}]^{13+}$	13	1044.1037	1044.1004	13573.3481	-3.3	23.75	475456
$[\mathbf{L}_{16}+Pd_{12}(BF_4)_{12}]^{12+}$	12	1138.3627	1138.3592	13660.3524	-3.5	24.99	1186965
$[\mathbf{L}_8+Pd_6(BF_4)_6]^{6+}$	6	1138.282	1138.2758	6829.692	-6.2	26.34	636016
$[\mathbf{L}_{16}+Pd_{12}(BF_4)_{13}]^{11+}$	11	1249.7575	1249.7558	13747.3325	-1.7	26.13	382230
$[\mathbf{L}_8+Pd_6(BF_4)_7]^{5+}$	5	1383.3392	1383.3318	6916.696	-7.4	30.28	173436
$[\mathbf{L}_{16}+Pd_{12}(BF_4)_{14}]^{10+}$	10	1383.4347	1383.4318	13834.347	-2.9	27.44	153648
$[\mathbf{L}_{16}+Pd_{12}(BF_4)_{15}]^{9+}$	9	1546.8148	1546.8136	13921.3332	-1.2	29.04	78380
$[\mathbf{L}_8+Pd_6(BF_4)_8]^{4+}$	4	1750.9169	1750.9158	7003.6676	-1.1	36.77	90406
$[\mathbf{L}_{16}+Pd_{12}(BF_4)_{16}]^{8+}$	8	1750.9138	1750.9158	14007.3104	2	30.95	36099

Table S2. Ions observed in IM-MS data for $Pd_{12}L_{16}$ and Pd_6L_8 mixture obtained using $[Pd(CH_3CN)_4](BF_4)_2$ in (modified RM1: M:**L** = 3:2 ratio). Ion interpretation, charge states (z), *m/z* values, mass accuracies, drift times (DT), and height of drift peak.

Mass spectra measured from the sample RM1 show a wide charge state distribution for both Pd_6**L_8** and Pd_{12}**L_{16}** depending on the number of BF_4^- counter anions. Peaks at *m/z* 730, 832, 963, 1138, 1383, and 1751 show overlapping isotopic distributions for both coordination complexes. Closer examination using IM-MS aided to obtain individual MS spectra and collision cross sections ($^{DT}CCS_{N2}$) (Table S1). For another example, $^{DT}CCS_{N2}$ of $[Pd_{12}L_{16}(BF_4)_6]^{18+}$ (*m/z* 729.961) and $[Pd_6L_8(BF_4)]^{11+}$ (*m/z* 581.425) were 2676 Å and 1464 Å, resulting 5.8 nm and 4.3 nm, respectively. This provides an excellent fit for Pd_{12}**L_{16}** and moderate fit for Pd_6**L_8** with the sizes observed using ^1H DOSY NMR spectra (Fig. S15, 5.8 nm for Pd_{12}**L_{16}** and 4.8 nm for Pd_6**L_8**) and computational models (5.5 nm for Pd_{12}**L_{16}** and 3.6 nm for Pd_6**L_8**) (Fig. S40). Please note that CCS is not directly showing geometric dimension and it is affected by interactions with the drift gas.

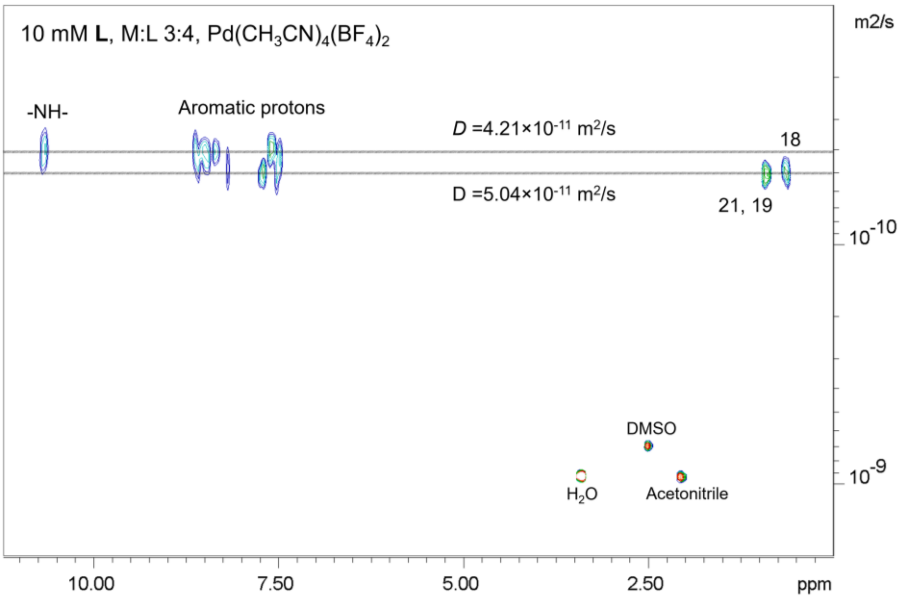

Figure S15. ^1H DOSY NMR spectra for the mixture of Pd_{12}**L_{16}** and Pd_6**L_8** in $[D_6]$-DMSO at 303.2 K and 700 MHz. Because of relatively broad and overlapping signals in ^1H DOSY NMR spectrum we could detect only a few signals originating from the smaller Pd_6**L_8** species. The hydrodynamic diameters calculated using the Stokes-Einstein equation are 5.8 nm (Pd_{12}**L_{16}**) and 4.8 nm (Pd_6**L_8**).

*2.2.2 Transformation reactions between Pd_6**L_8** and Pd_{12}**L_{16}** of RM1*

2.2.2.1 Acetonitrile removal

The sample prepared *via* RM1 was dried under vacuum at 40 °C for 24 h, the residue was suspended in $[D_6]$-DMSO solution and heated at 70 °C. Only a small fraction of the residue dissolved after 1 h heating, but it eventually fully dissolved after another 23 h. The final sample was studied using ^1H- and ^1H DOSY NMR spectroscopy which showed only a trace amount of acetonitrile left (starting from conditions RM1, the ratio between Pd:ACN is reduced from 1:4 to 1:0.2) (Fig. S16).

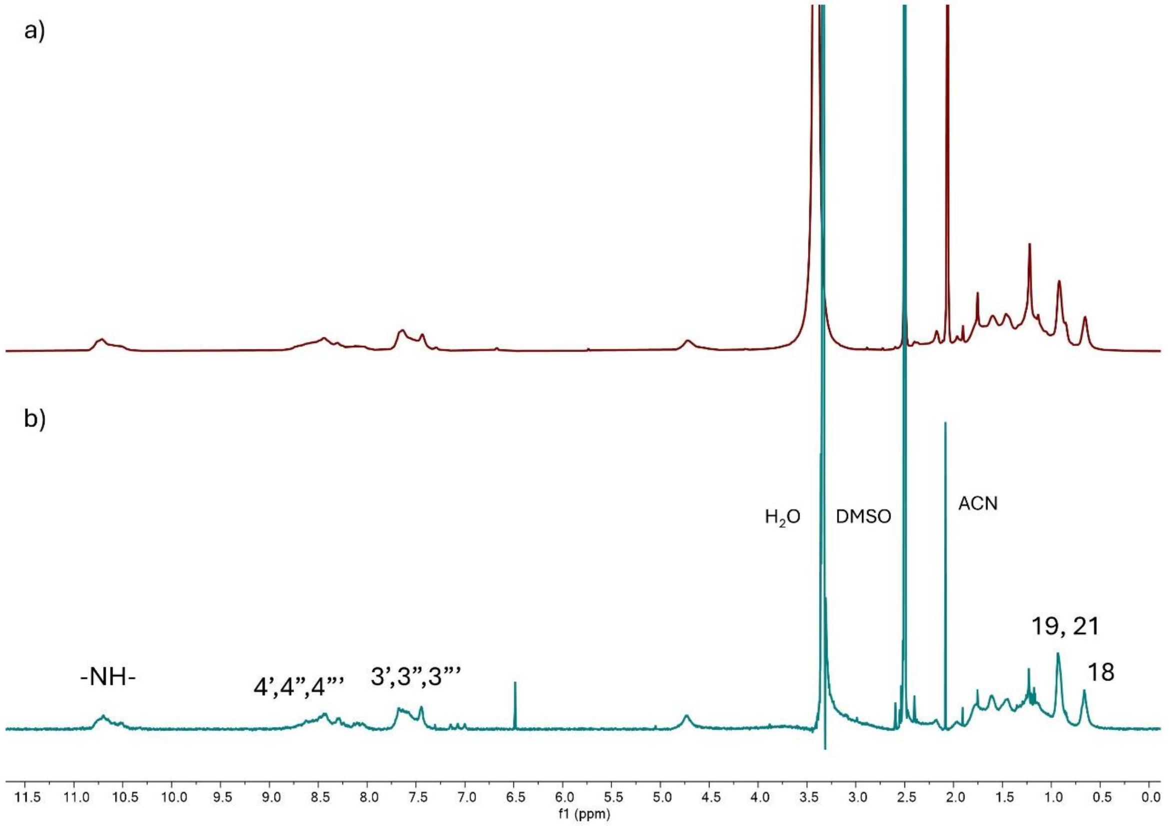

Figure S16. ^{1}H NMR spectrum of: a) original mixture prepared *via* conditions RM1 providing a mixture of $Pd_6\mathbf{L}_8$ and $Pd_{12}\mathbf{L}_{16}$ as confirmed by IM-MS and ^{1}H DOSY NMR, b) the mixture upon evaporation to dryness and redissolving in [D_6]-DMSO (stirred at 70 °C for 24 h). There is about 20 × decrease of ACN content (changing the Pd:ACN ratio from 1:4 to only 1:0.2), which is accompanied by a structural transformation of the whole fraction of $Pd_6\mathbf{L}_8$ into $Pd_{12}\mathbf{L}_{16}$ as confirmed by ^{1}H DOSY NMR.

The ^{1}H DOSY NMR spectrum shows complete conversion of the $Pd_{12}\mathbf{L}_{16}$ and $Pd_6\mathbf{L}_8$ mixture into $Pd_{12}\mathbf{L}_{16}$ (Fig. S17).

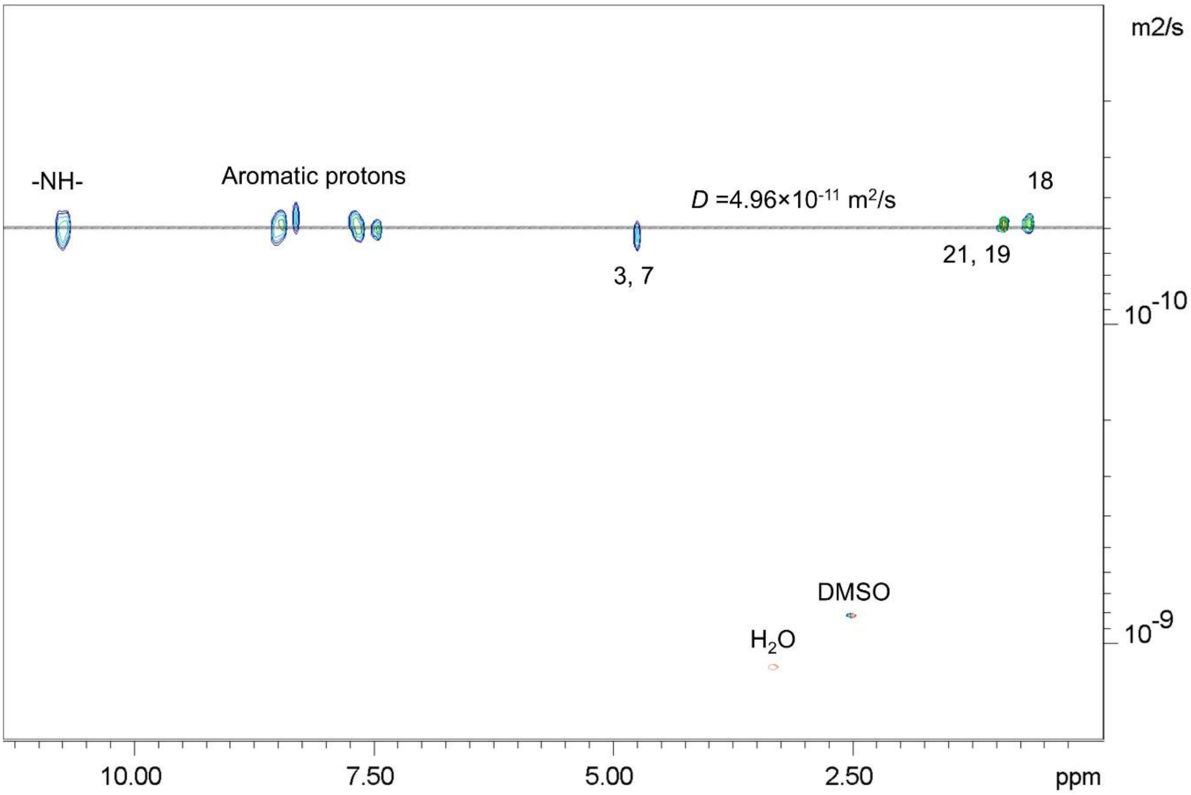

Figure S17. ^{1}H DOSY NMR spectrum of sample prepared from the mixture of Pd_6**L**$_8$ and Pd_{12}**L**$_{16}$ species prepared using $[Pd(CH_3CN)_4](BF_4)_2$ and **L** in M:**L** 3:4 ratio in [D_6]-DMSO (RM1). The mixture was evaporated to dryness to remove maximum of ACN (vacuum, 24 h at 40 °C) and the residue was redissolved in [D_6]-DMSO (stirred at 70 °C for 24 h). Structural transformation of the whole fraction of Pd_6**L**$_8$ into Pd_{12}**L**$_{16}$ was confirmed.

2.2.2.2 Addition of $TBANO_3$

The experiment of adding $TBANO_3$ to the RM1 was performed and followed by ^{1}H- and ^{1}H DOSY NMR as the IM-MS measurement did not provide conclusive results. 1.5 eq. (M:**L**:NO_3 3:4:6) and 3 eq. (M:**L**:NO_3 3:4:12) of $TBANO_3$ with respect to **L** were subsequently added to the DMSO-d_6 solution of RM1 and the reaction mixture was heated at 70 °C for 1 h upon every addition (Fig. S18a, b). The ^{1}H DOSY NMR analysis shows that the solutions contain mixtures of SCCs (even after continued heating for 24 h) (Fig. S18c).

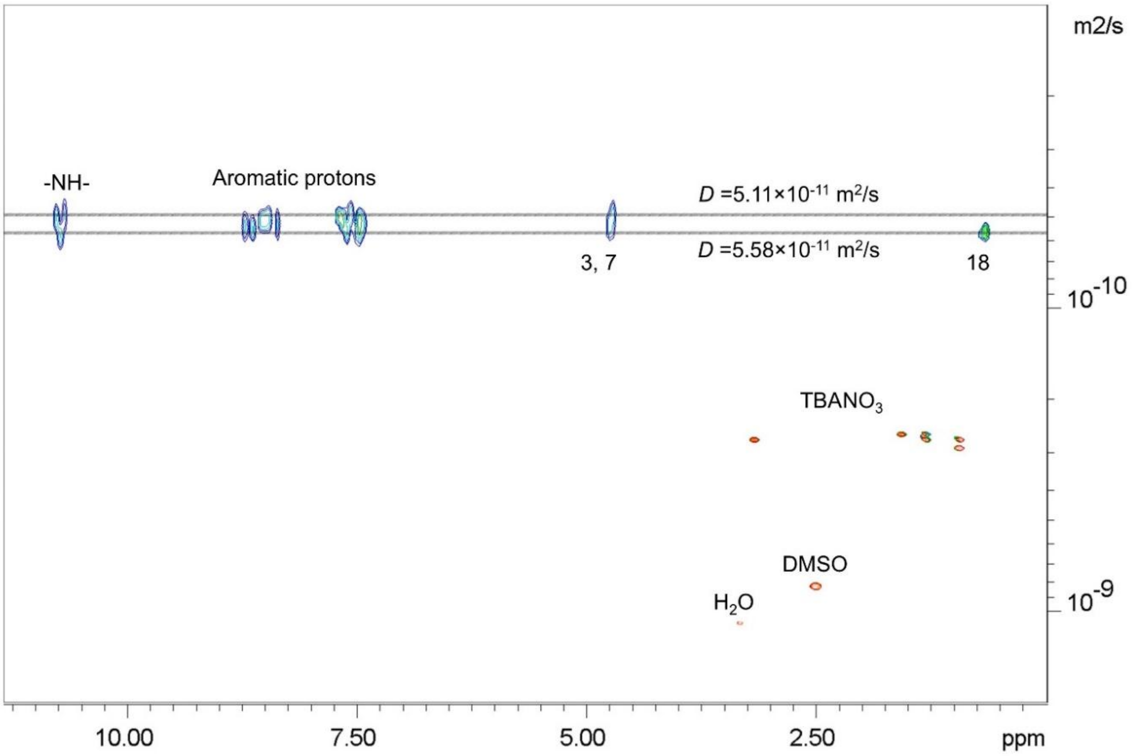
m2/s
-NH-
Aromatic protons
D =5.11×10-11 m2/s
D =5.58×10-11 m2/s
3, 7
18
10-10
TBANO3
DMSO
H2O
10-9
10.00
7.50
5.00
2.50
ppm

a)

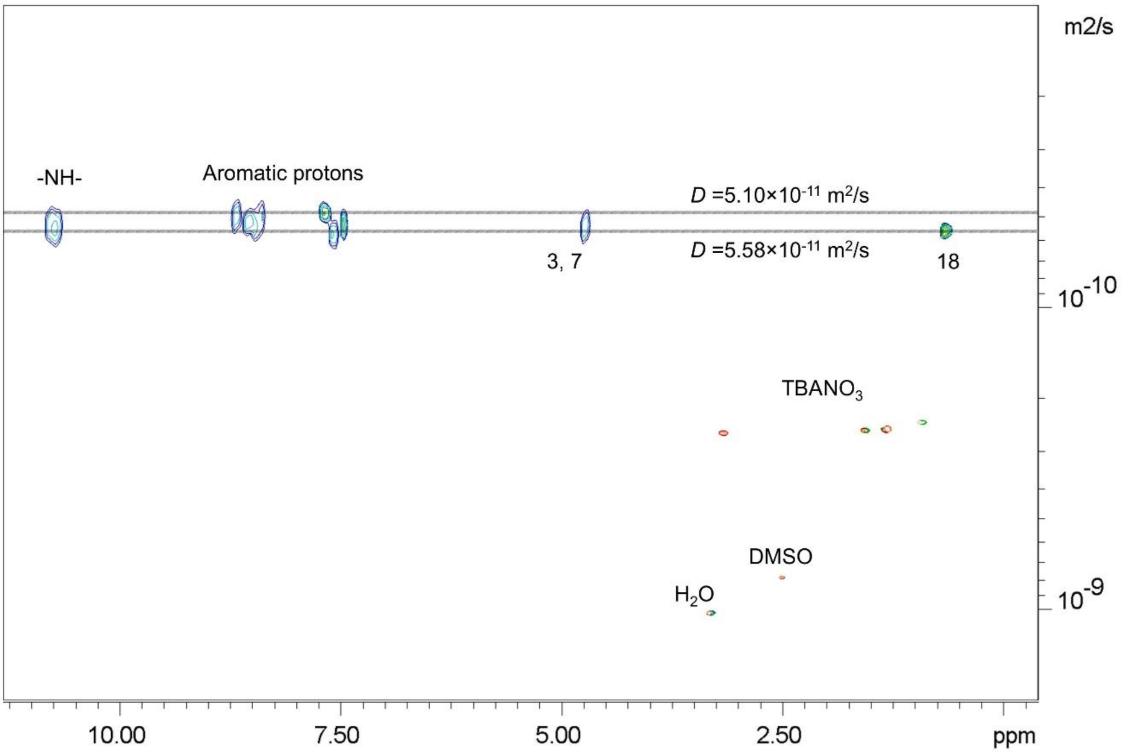
m2/s
-NH-
Aromatic protons
D =5.10×10-11 m2/s
D =5.58×10-11 m2/s
3, 7
18
10-10
TBANO3
DMSO
H2O
10-9
10.00
7.50
5.00
2.50
ppm

b)

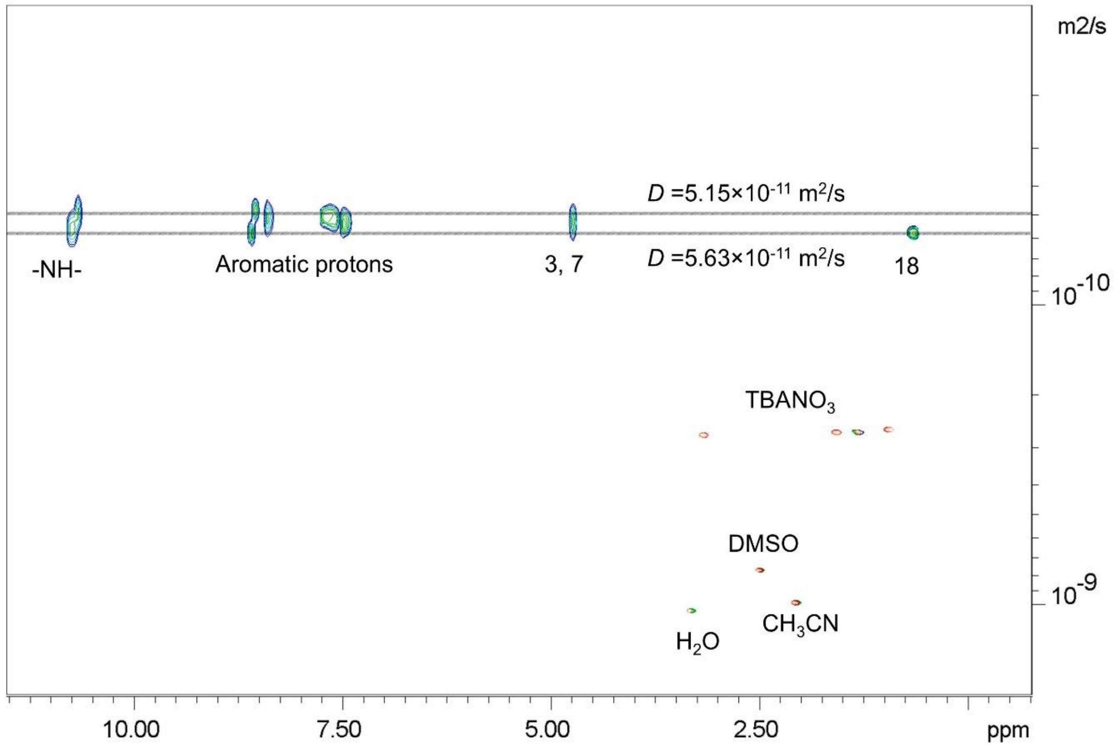

c)

Figure S18. ^{1}H-DOSY NMR spectrum of a mixture prepared using solution of L (10 mM) with $[Pd(CH_3CN)_4](BF_4)_2$ (M:L = 3:4) at 70 °C, 1 h. Subsequently, $TBANO_3$ was added and the mixture was heated: a) addition of 1.5 equiv. of $TBANO_3$ (15 mM $TBANO_3$, resulting in M:L:NO_3^- = 3:4:6, heated for 1 h; b) addition of another 1.5 equiv. of $TBANO_3$ (totaling 30 mM $TBANO_3$, M:L:NO_3^- = 3:4:12, heated for 1 h, c) the same mixture heated for 24 h.

To study the effect of introducing excess of nitrate from the beginning of the coordination reaction, 3 eq. of $TBANO_3$ were mixed with 10 mM [D_6]-DMSO solution of **L** and the resulting solution was added to $[Pd(CH_3CN)_4]BF_4$ and heated at 70 °C. Heating for 1 h showed very broad and low-intensity ^{1}H NMR signals, whereas prolonged heating for 24 h only showed enhancement in the ^{1}H NMR signal intensity (Fig. S19). This reaction similarly resulted in a mixture of both complexes as confirmed by ^{1}H DOSY NMR (Fig. S20). These experiments indicate that the addition of NO_3^- does not influence strongly the equilibrium between both SCCs and it is rather the Pd concentration or ACN content that can control interspatial transformation and stir the equilibrium towards Pd_6**L**$_8$ product.

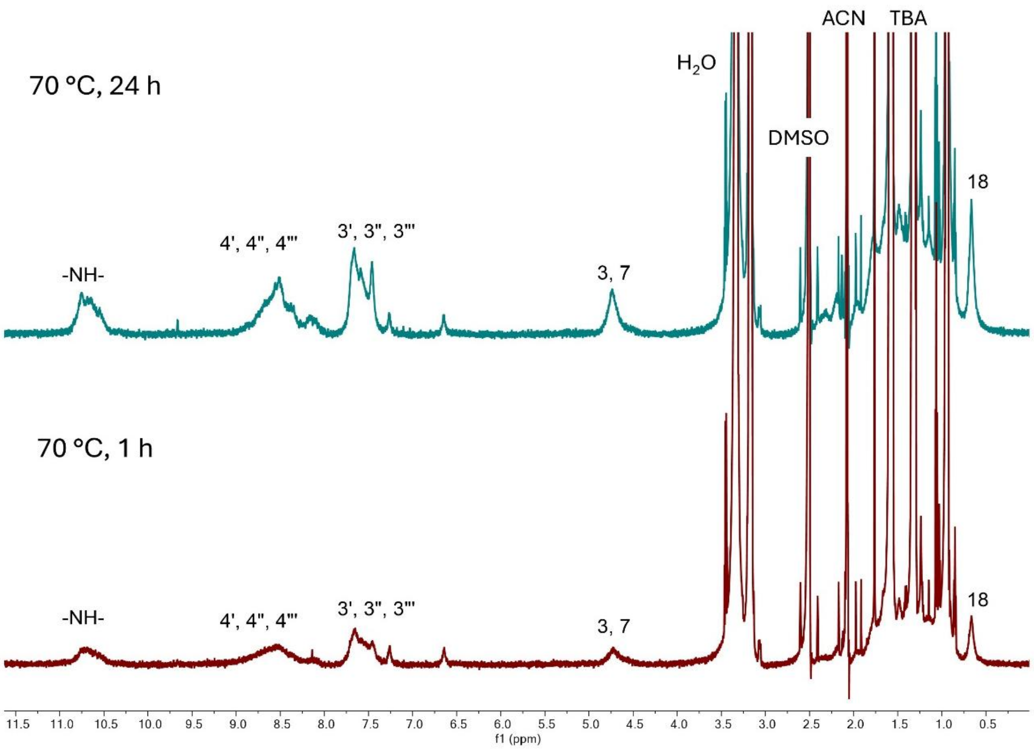

Figure S19. ^{1}H NMR spectra after 1 h and 24 h of reaction performed using solution of L (10 mM) with 3 equiv. of $TBANO_3$ (30 mM) to dissolve $[Pd(CH_3CN)_4](BF_4)_2$ (M:L = 3:4) followed by heating at 70 °C.

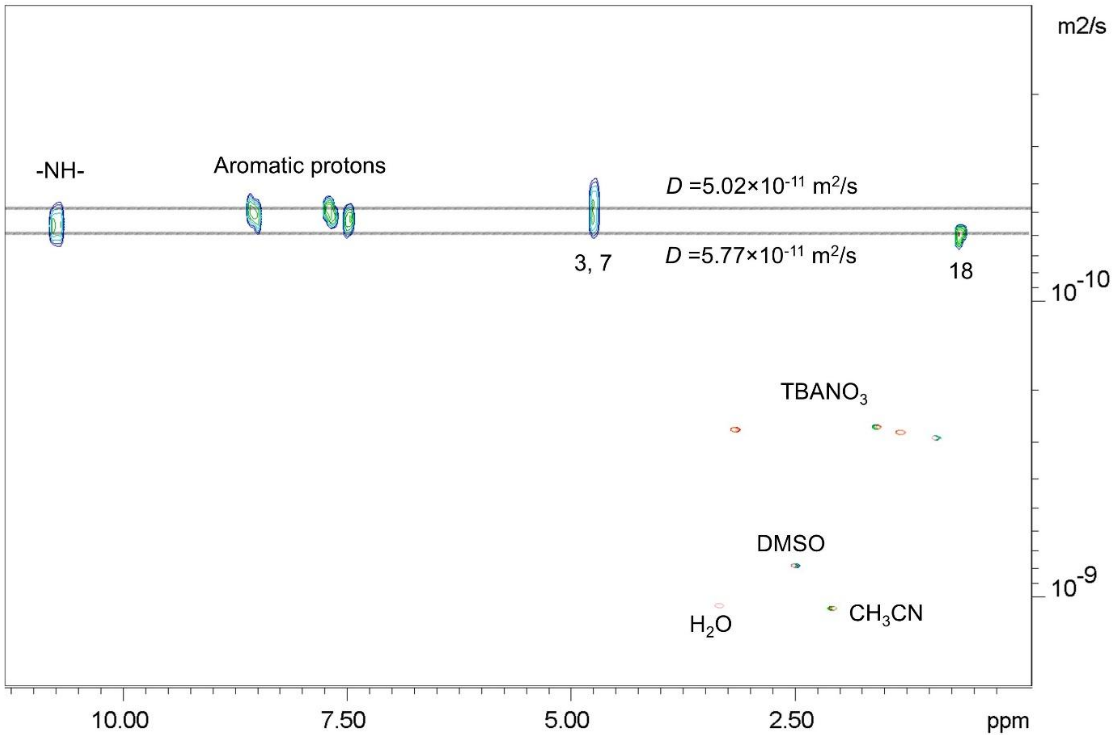

Figure S20. ^{1}H-DOSY NMR spectrum of a mixture prepared using solution of **L** (10 mM) with 3 equiv. of $TBANO_3$ (30 mM) to dissolve $[Pd(CH_3CN)_4](BF_4)_2$ (M:**L** = 3:4). The mixture was heated at 70 °C for 24 h.

2.2.3 $Pd(NO_3)_2{\cdot}2H_2O$ in [D_6]-DMSO (RM2 and RM2 3:2)

Complexation reactions were performed with 10 mM [D_6]-DMSO (500 µL) solution of **L** (3.5 mg) which was mixed with $Pd(NO_3)_2{\cdot}2H_2O$ (1 mg for M:**L** 3:4 resulting in Pd_{12}**L**$_{16}$ (RM2) and 2 mg M:**L** 3:2 resulting in Pd_6**L**$_8$ (RM2 3:2)). The solutions were heated at 70 °C for 1 hour.

^{1}H NMR (700 MHz, [D_6]-DMSO, 298.2 K): δ = 11.00-10.30 (m, -NH-), 9.10-7.90 (m, H-4', H-4" and H-4"'), 7.90-7.20 (m, H-3', H-3" and H-3"'), 4.95-4.30 (m, H-3 and H-7), 0.40-2.45 (overlapping steroid skeleton) ppm.

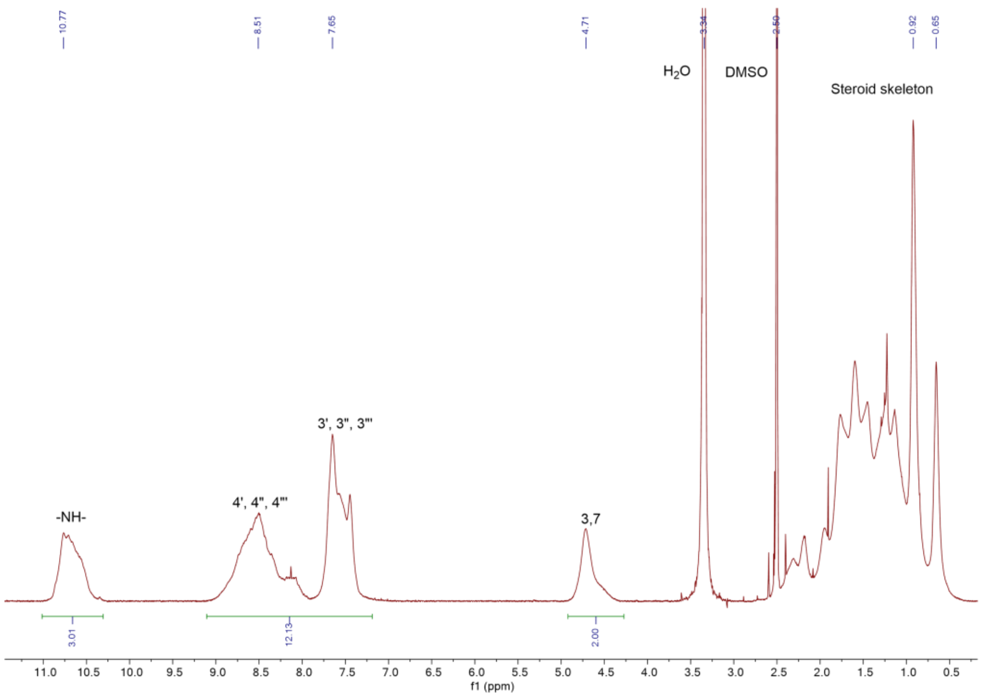

Figure S21. ^{1}H NMR spectra of Pd_{12}**L**$_{16}$ in [D_6]-DMSO at 298.2 K and 700 MHz (RM2).

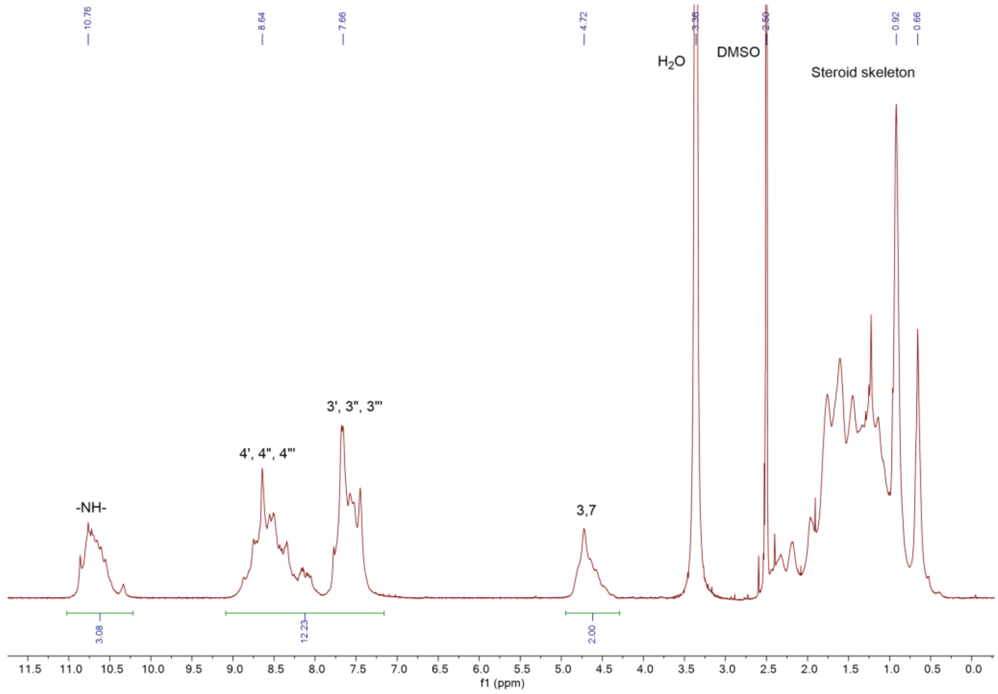

Figure S22. ^{1}H NMR spectra of Pd_6**L_8** in [D_6]-DMSO at 298.2 K and 700 MHz (RM2 3:2).

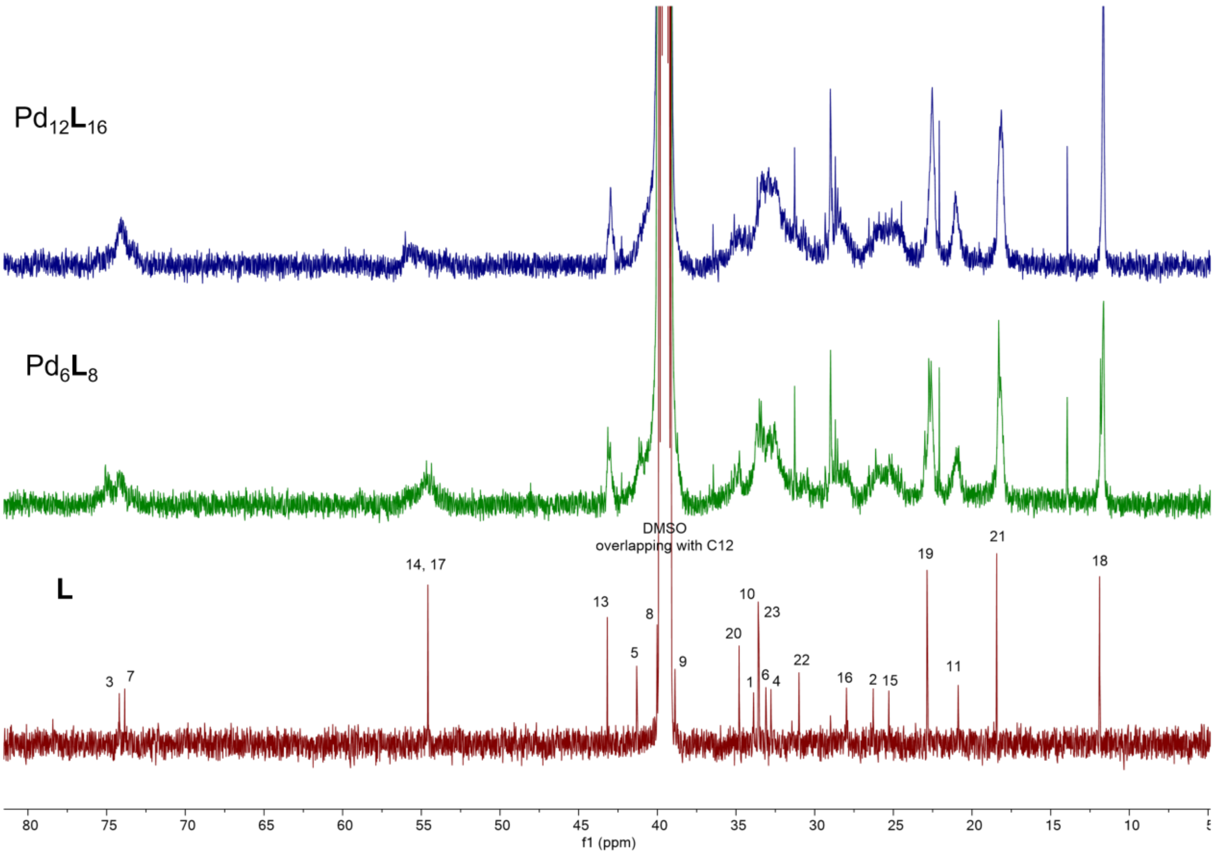

Figure S23. Comparison of ^{13}C NMR spectra (aliphatic region) of **L**, Pd_6**L_8** (RM2 3:2), and Pd_{12}**L_{16}** (RM2) in [D_6]-DMSO at 298.2 K and 700 MHz.

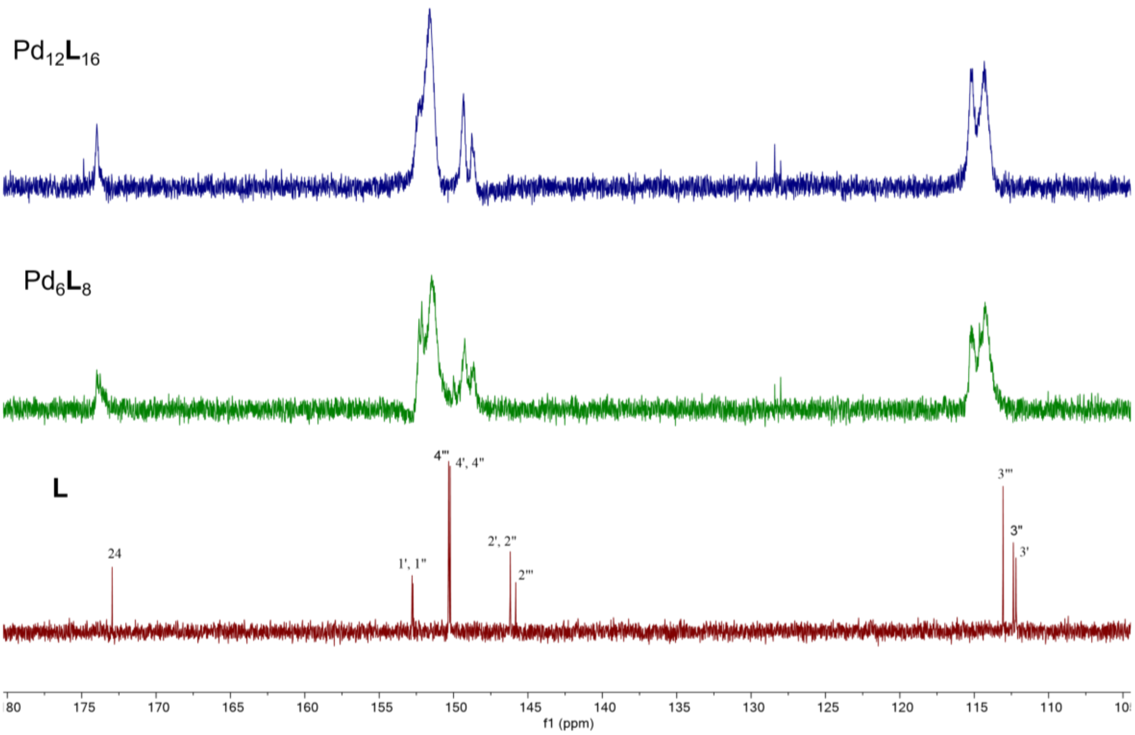

Figure S24. Comparison of ^{13}C NMR spectra (aromatic and carbonyl region) of **L**, Pd_6**L_8** (RM2 3:2), and Pd_{12}**L_{16}** (RM2) in [D6]-DMSO at 298.2 K and 700 MHz.

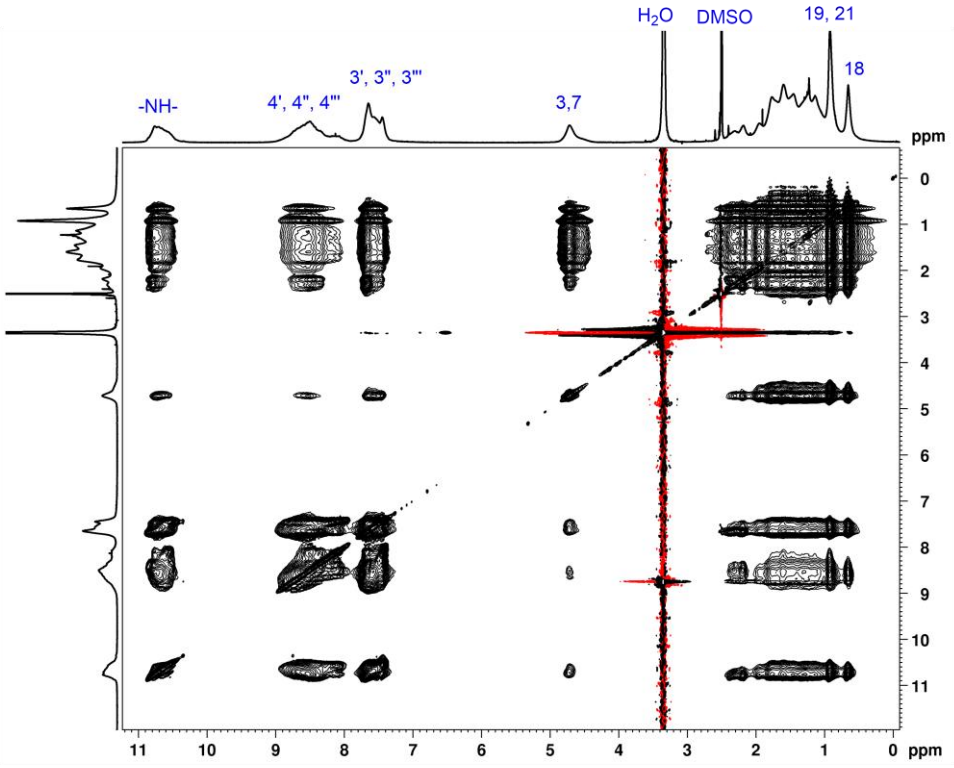

Figure S25. ^1H-^1H NOESY NMR spectra of Pd_{12}**L_{16}** (RM2) in [D6]-DMSO at 298.2 K and 700 MHz.

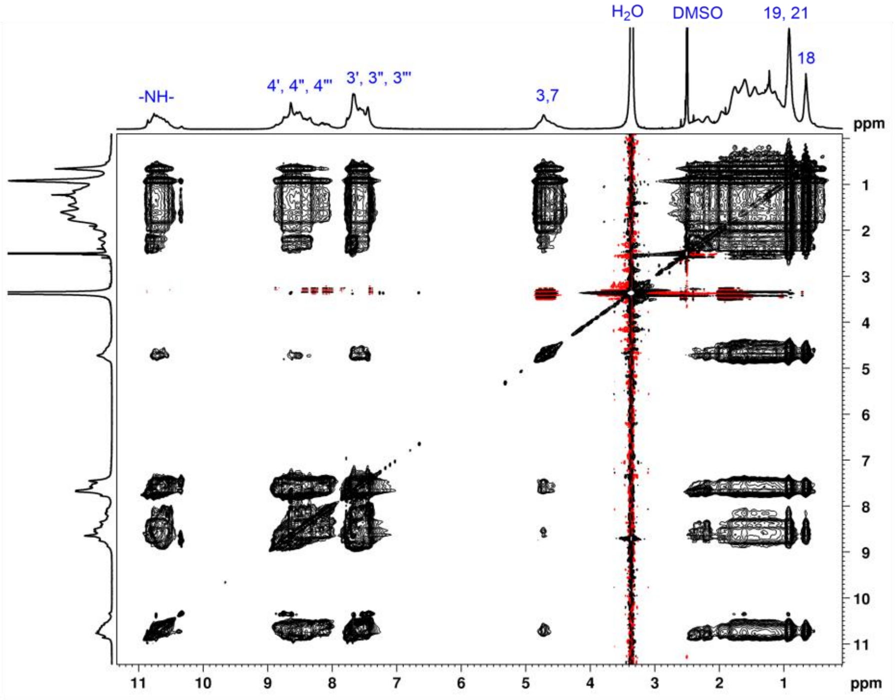

Figure S26. ^{1}H-^{1}H NOESY NMR spectra of Pd_6**L_8** (RM2 3:2) in [D_6]-DMSO at 298.2 K and 700 MHz.

2.2.4 Variable temperature (VT) NMR spectroscopic study of RM2

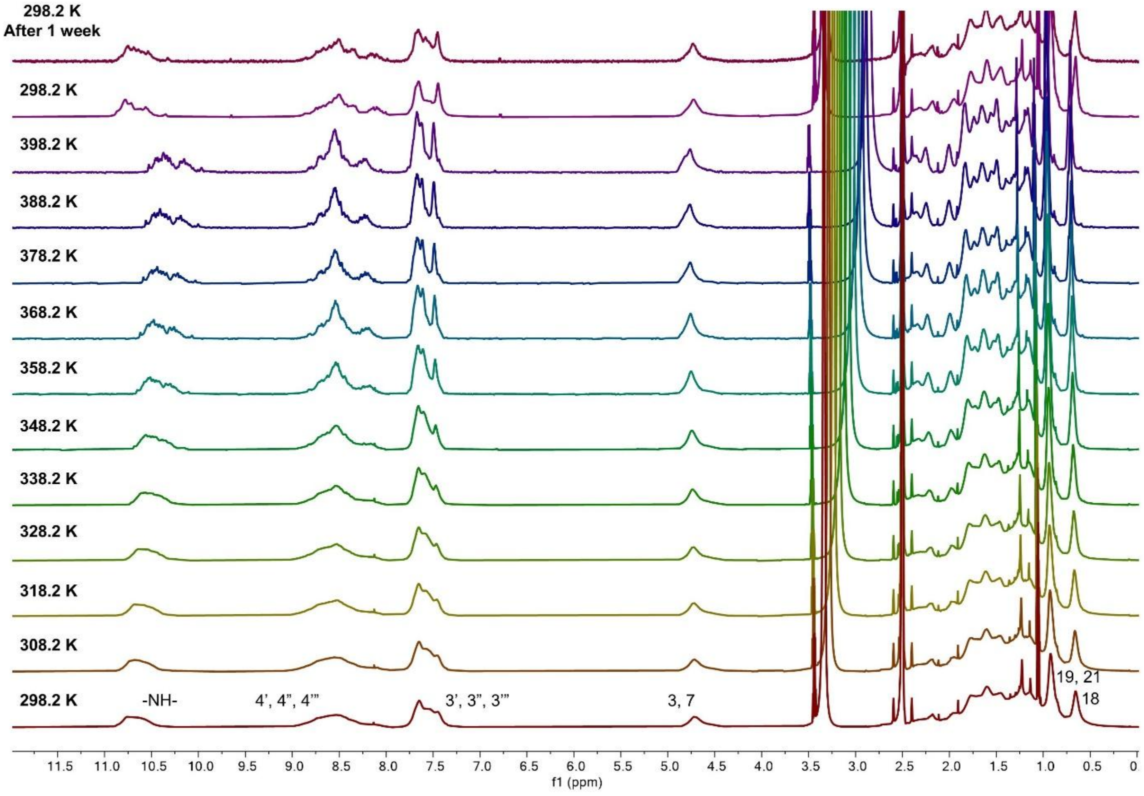

Figure S27. VT ^{1}H NMR experiment using Pd_{12}**L_{16}** (prepare *via* RM2: M:L 3:4, [D_6]-DMSO, 70 °C, 1 h) in range 298.2-398.2 K (10 K/step).

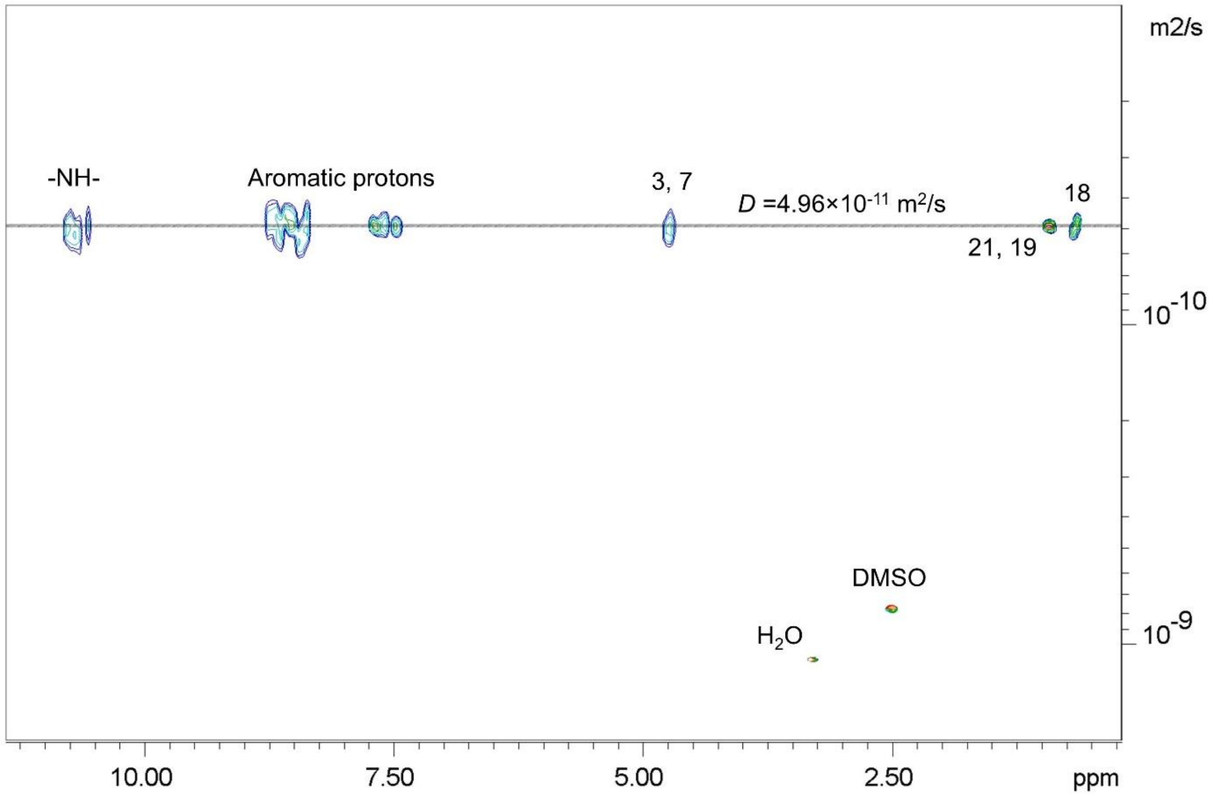

Figure S28. ^{1}H-DOSY NMR spectrum of $Pd_{12}\mathbf{L}_{16}$ (*via* RM2) heated from 298.2 to 398.2 K (10 K/step), cooled down back to 298.2 K and measured.

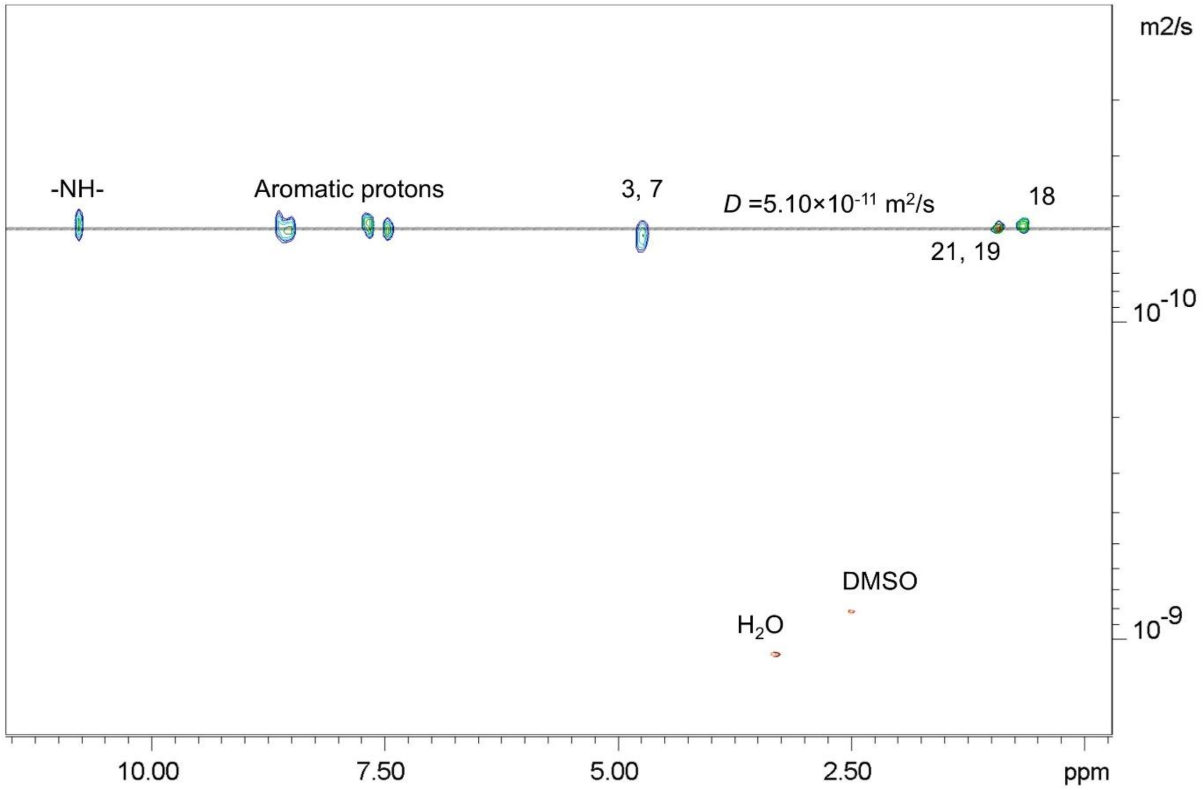

Figure S29. ^{1}H-DOSY NMR spectrum of $Pd_{12}\mathbf{L}_{16}$ (*via* RM2) heated for 1 h at 100 °C, cooled down, and measured at 298.2 K.

2.2.5 $Pd(NO_3)_2{\cdot}2H_2O$ in 5% (v/v) [D_3]-ACN in [D_6]-DMSO solvent mixture (RM3)

Complexation reaction was performed with 10 mM solution of **L** in 5% (*v/v*) [D_3]-ACN in [D_6]-DMSO solvent mixture (500 µL, 3.5 mg of **L**) using $Pd(NO_3)_2{\cdot}2H_2O$ (M:L 3:4, 2 mg). The resulting solution was heated at 70 °C for 1 hour yielding selectively Pd_6**L**$_8$ (RM3).

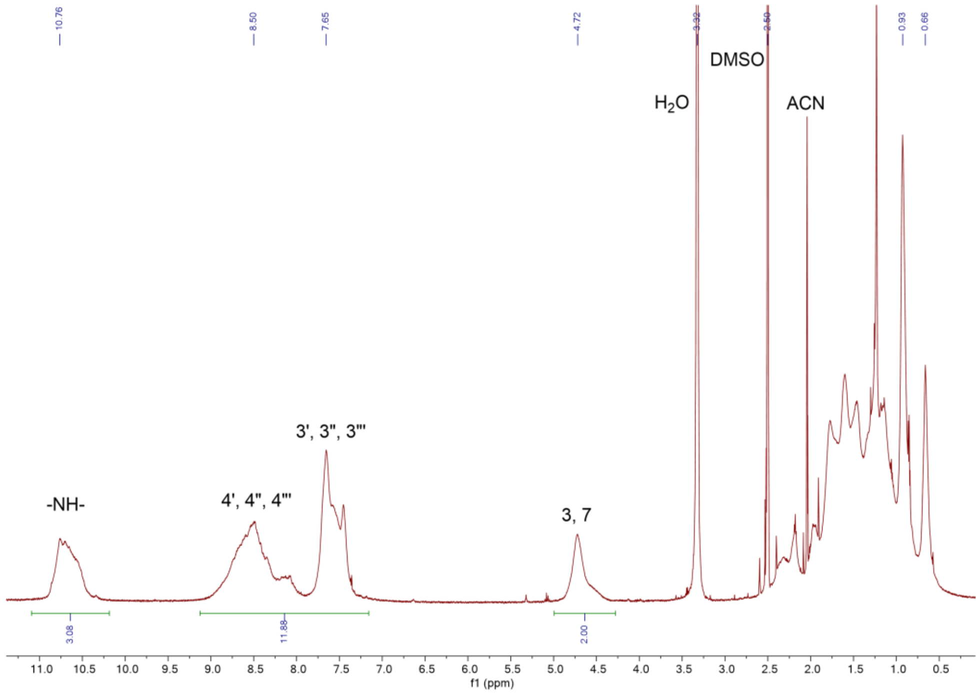

Figure S30. ^{1}H NMR spectra of Pd_6**L**$_8$ in 5% (*v/v*) [D_3]-ACN in [D_6]-DMSO solvent mixture at 298.2 K and 700 MHz.

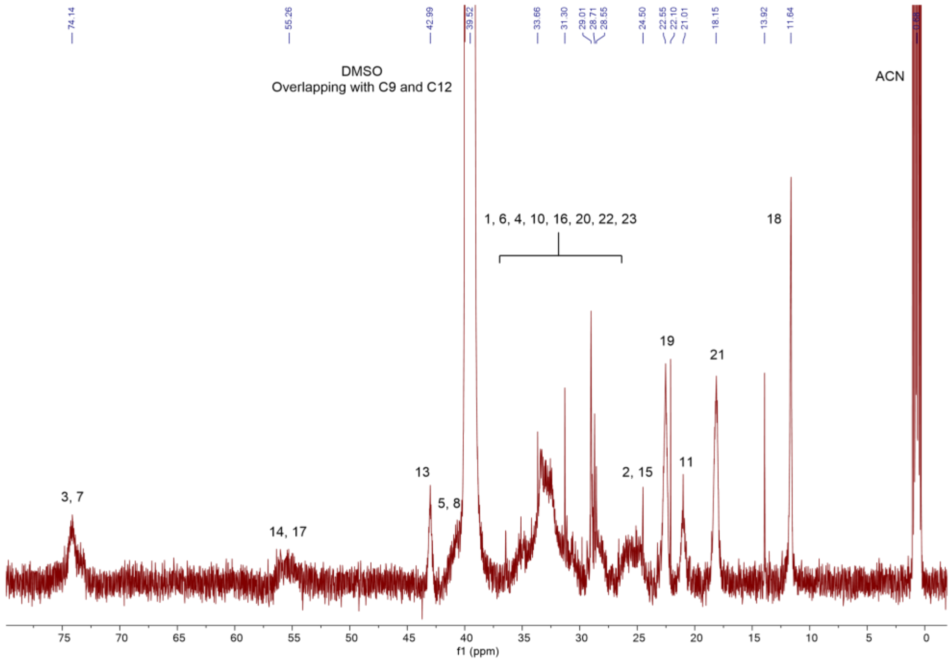

Figure S31. ^{13}C NMR spectra (aliphatic region) of Pd_6L_8 using 5% (*v/v*) [D_3]-ACN solvent mixture in [D_6]-DMSO at 298.2 K and 700 MHz.

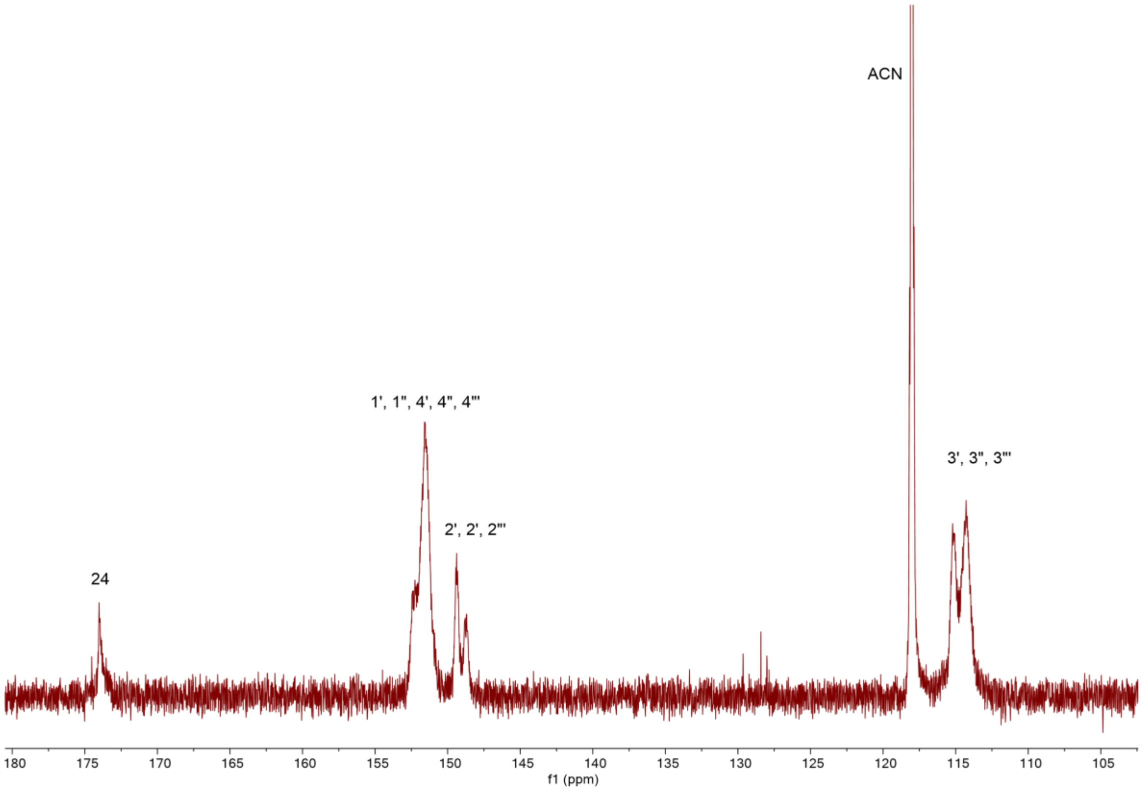

Figure S32. ^{13}C NMR spectra (aromatic and carbonyl regions) of Pd_6**L**$_8$ using 95:5 [D_3]-ACN:[D_6]-DMSO solvent mixture at 298.2 K and 700 MHz.

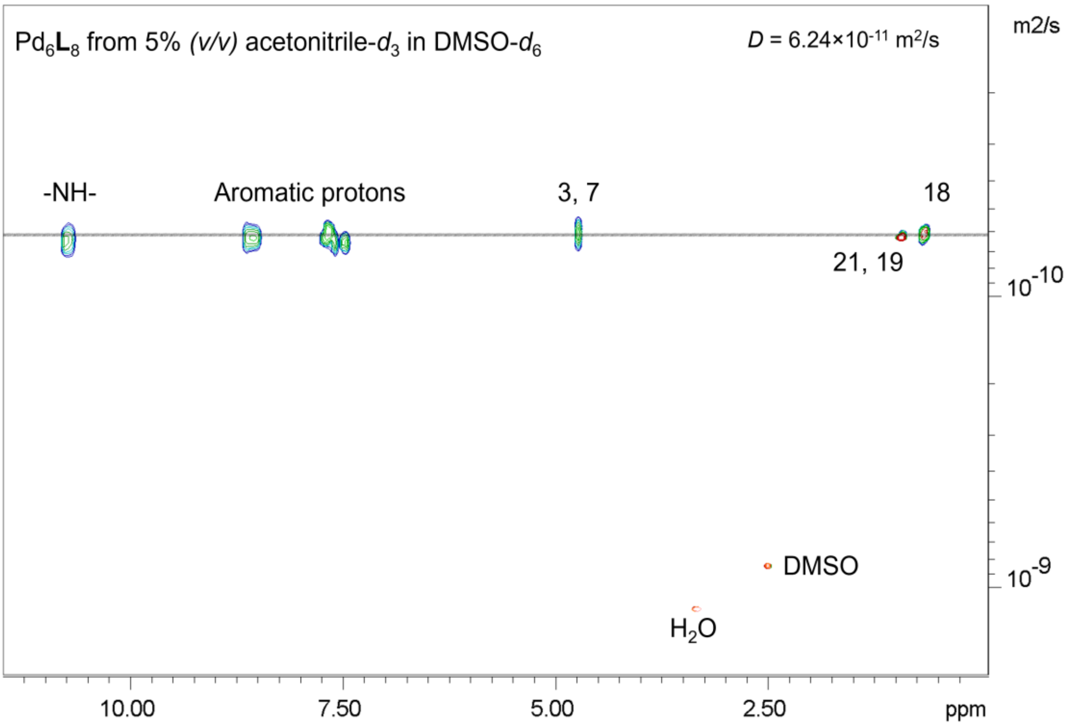

Figure S33. ^{1}H DOSY NMR spectra of Pd_6**L**$_8$ using 95:5 [D_3]-ACN:[D_6]-DMSO solvent mixture at 303.2 K and 700 MHz (acetonitrile being less viscous compared to DMSO, resulting in higher diffusion of complex than in neat DMSO).

*2.2.6 Transformation reactions between Pd_6**L**$_8$ and Pd_{12}**L**$_{16}$ of RM2 and RM2 3:2*

2.2.6.1 Addition of acetonitirile to Pd_{12}**L**$_{16}$ of RM2

The experiment was performed using 10 mM **L** (3.5 mg) with $Pd(NO_3)_2.2H_2O$ (1 mg) in M:**L** 3:4 ratio in 500 µL DMSO-d_6. The reaction mixture was kept at 70 °C for 1 h. This was subsequently followed by an addition of 25 µL of [D_3]-ACN to 475 µL of the reaction mixture and heated at 70 °C for 1 h. The ^{1}H- and ^{1}H DOSY NMR were measured after the first and the second complexation (Fig. S34 and S35). The resulting spectra show total conversion of Pd_{12}**L**$_{16}$ into Pd_6**L**$_8$.

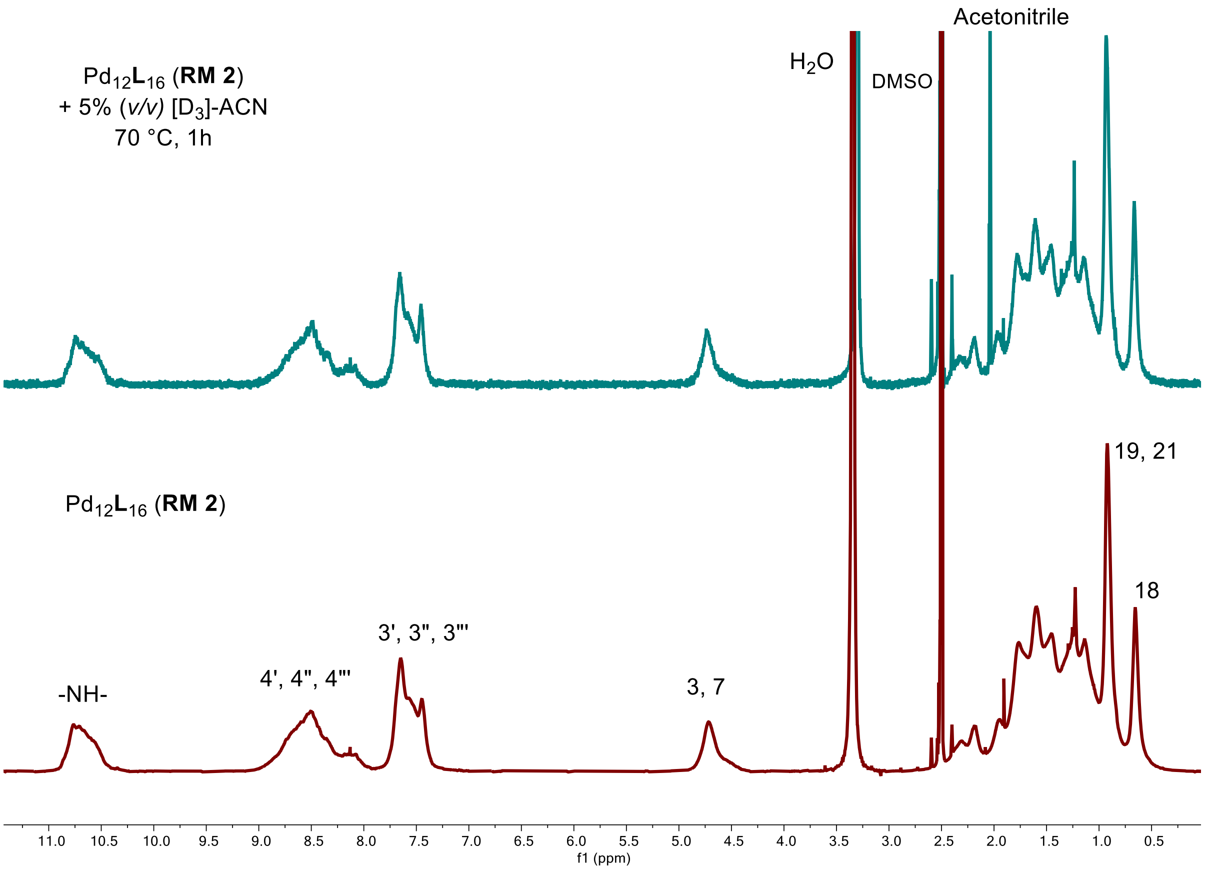

Figure S34. Transformation of Pd_{12}**L**$_{16}$ upon addition of [D_3]-ACN as followed by ^{1}H NMR spectroscopy (5% [D_3]-ACN in [D_6]-DMSO, 298.2 K, 700 MHz).

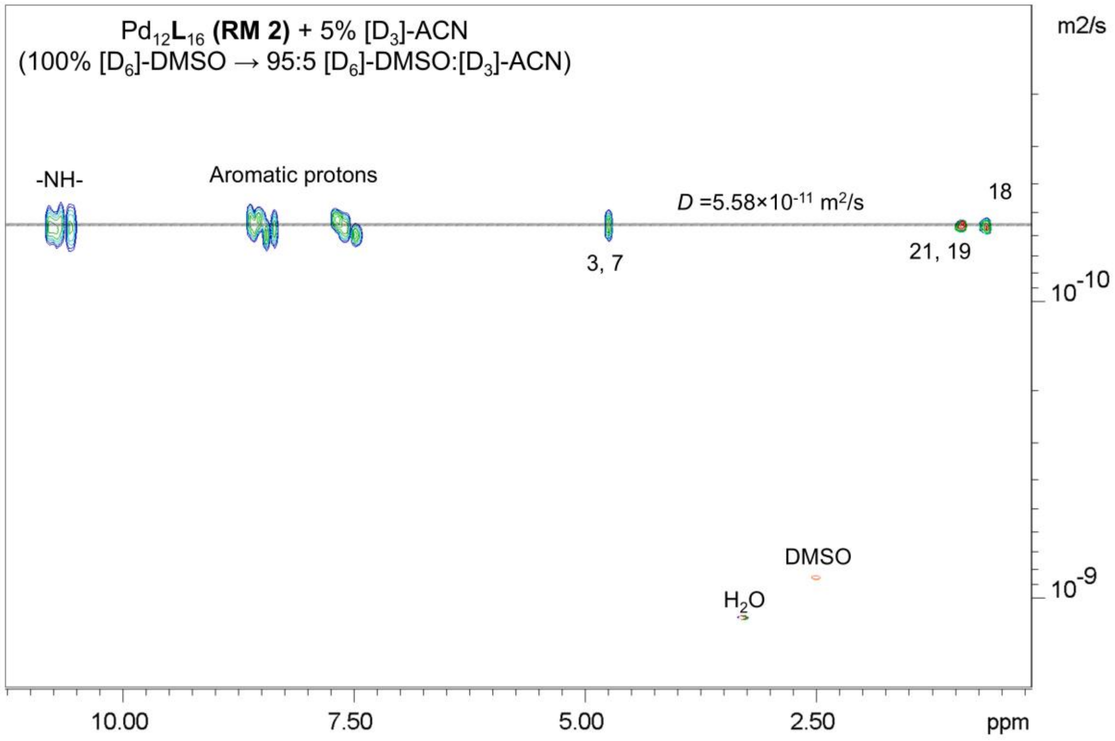

Figure S35. Transformation of $Pd_{12}\mathbf{L}_{16}$ upon addition of $[D_3]$-ACN as followed by ^{1}H DOSY NMR spectroscopy (5% $[D_3]$-ACN in $[D_6]$-DMSO, 298.2 K, 700 MHz).

2.2.6.2 Addition of ligand to $Pd_6\mathbf{L}_8$ of RM2 3:2

The experiment was performed using 10 mM **L** (3.5 mg) with $Pd(NO_3)_2.2H_2O$ (2 mg) in M:**L** 3:2 ratio in 500 µL DMSO-d_6. The reaction mixture was kept at 70 °C for 1 h. This was subsequently followed by an addition of 3.5 mg of **L** and heating at 70 °C for 1 h. The ^{1}H- and ^{1}H DOSY NMR were measured after the first and the second complexation (Fig. S36 and S37). The resulting spectra show total conversion of $Pd_6\mathbf{L}_8$ into $Pd_{12}\mathbf{L}_{16}$.

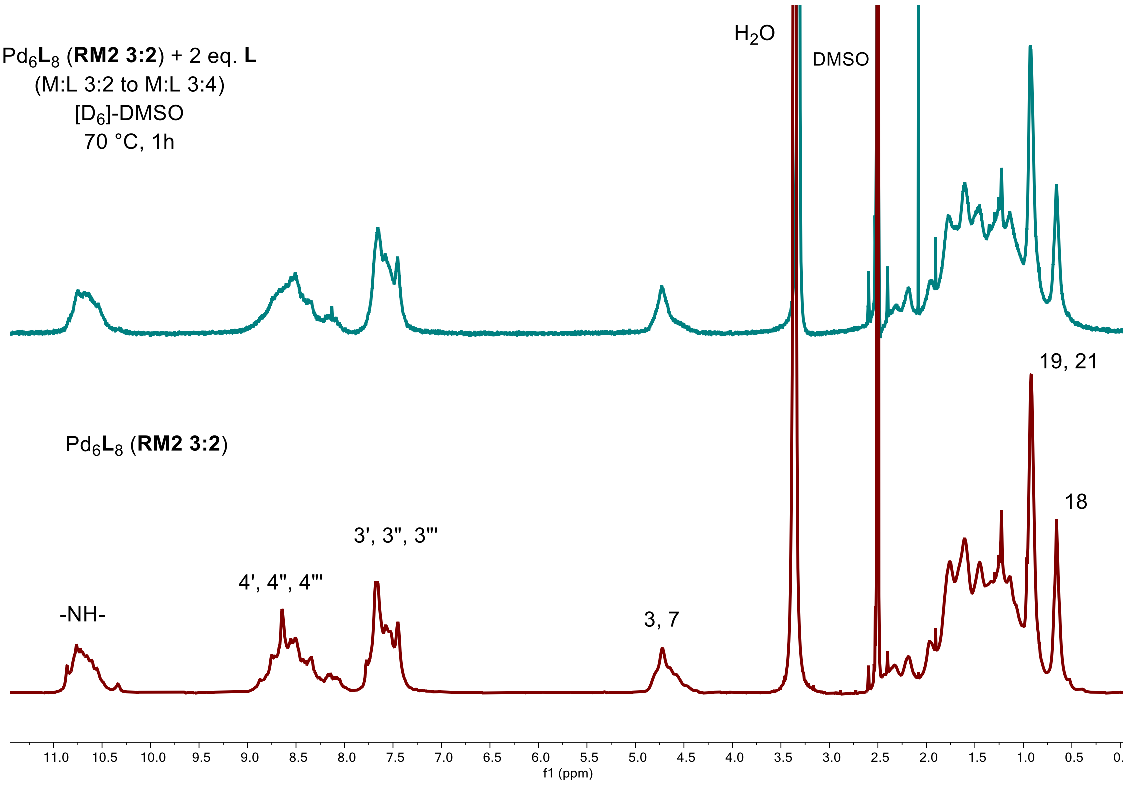

Figure S36. Transformation of Pd_6**L**$_8$ upon addition of **L** as followed by ^{1}H NMR spectroscopy ([D$_6$]-DMSO, 298.2 K, 700 MHz).

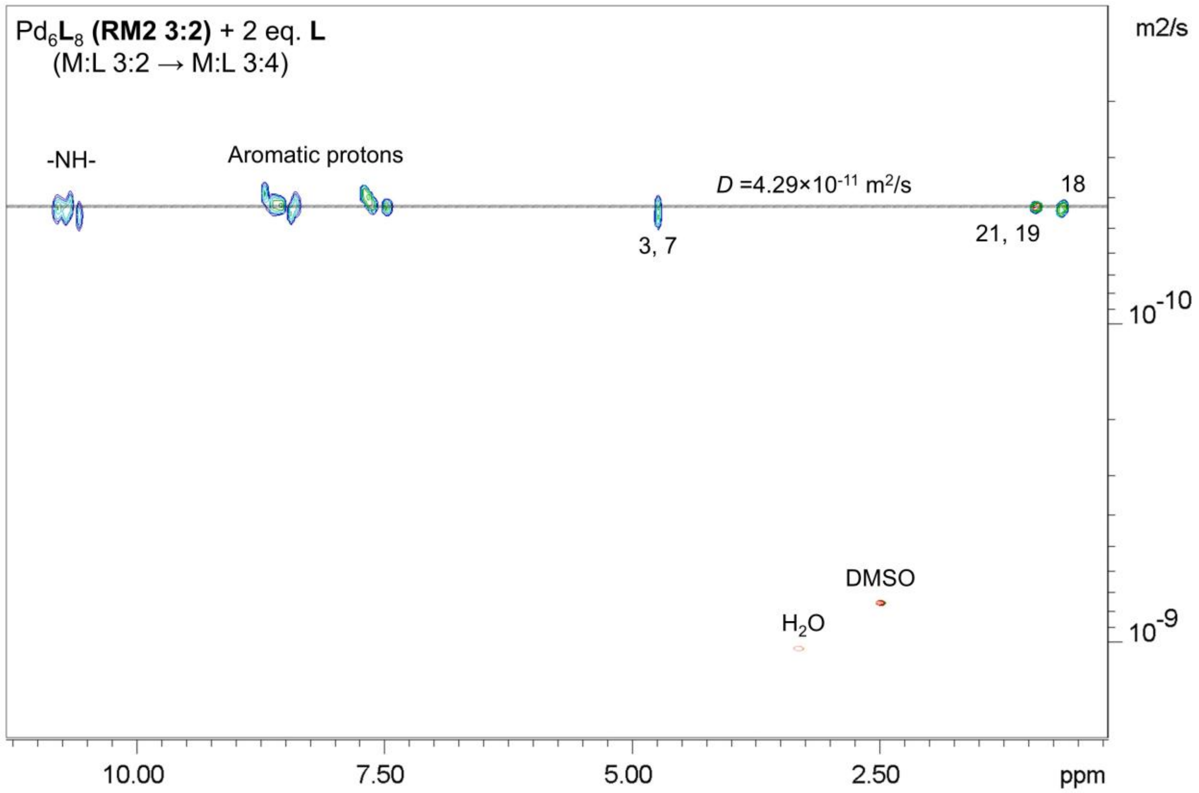

Figure S37. Transformation of Pd_6**L**$_8$ upon addition of **L** as followed by ^{1}H DOSY NMR spectroscopy ([D$_6$]-DMSO, 298.2 K, 700 MHz).

2.2.6.3 Addition of palladium(II) nitrate to Pd_{12}**L**$_{16}$ of RM2

The experiment was performed using 10 mM **L** (3.5 mg) with $Pd(NO_3)_2.2H_2O$ (1 mg) in M:**L** 3:4 ratio in 500 µL DMSO-d_6 (conditions of RM2). The reaction mixture was kept at 70 °C for 1 h. This was subsequently followed by an addition of 1 mg of $Pd(NO_3)_2.2H_2O$ and consequential heating at 70 °C for 1 h. The ^{1}H- and ^{1}H DOSY NMR were measured after the first and the second complexation (Fig. S38 and S39). The resulting spectra show partial conversion of Pd_{12}**L**$_{16}$ into Pd_6**L**$_8$. Prolonged heating did not change the outcome of the reaction as followed by ^{1}H DOSY NMR spectroscopy.

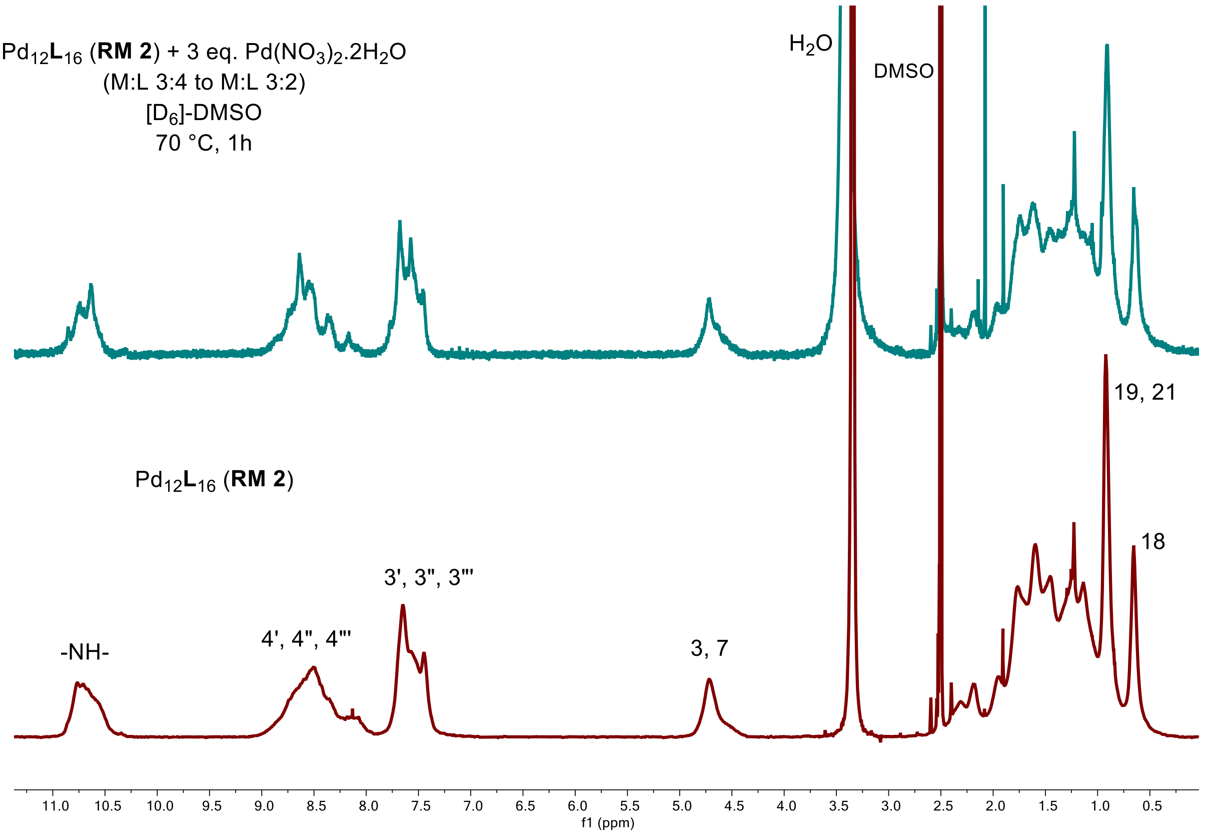

Figure S38. Transformation of Pd_{12}**L**$_{16}$ upon addition of $Pd(NO_3)_2$ as followed by ^{1}H NMR spectroscopy ([D_6]-DMSO, 298.2 K, 700 MHz).

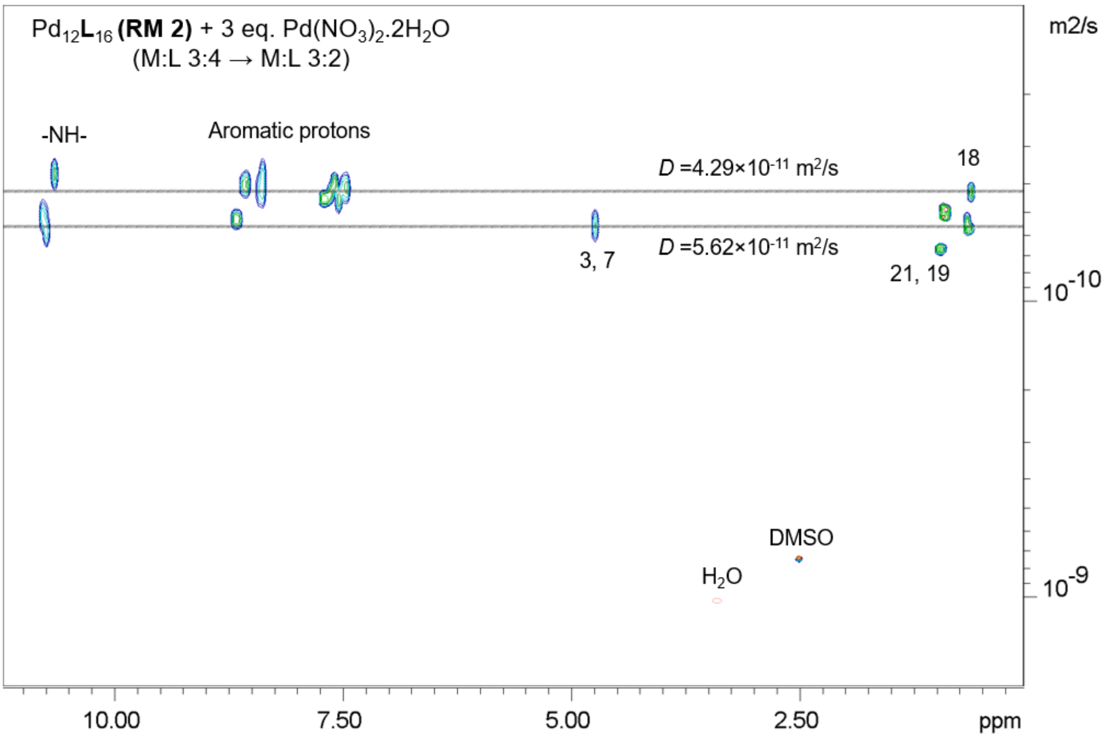

Figure S39. Transformation of $Pd_{12}\mathbf{L}_{16}$ upon addition of $Pd(NO_3)_2$ as followed by ^{1}H DOSY NMR spectroscopy ([D$_6$]-DMSO, 298.2 K, 700 MHz).

Table S3. Comparison of diffusion coefficients (D) of products and residual internal solvent signal of [D6]-DMSO as obtained using ^{1}H DOSY NMR spectroscopy (700 MHz, 303.2 K).

Experiment	Reaction conditions	Product	$D_{product}$ (m^2/s)
RM1	$Pd(CH_3CN)_4(BF_4)_2$:**L** = 3:4 in [D$_6$]-DMSO	$Pd_6\mathbf{L}_8$ + $Pd_{12}\mathbf{L}_{16}$	4.21×10^{-11}<br>5.04×10^{-11}
RM1 – ACN	$Pd(CH_3CN)_4(BF_4)_2$:**L** = 3:4 in [D$_6$]-DMSO (evaporated and redissolved)	$Pd_{12}\mathbf{L}_{16}$	4.96×10^{-11}
RM1 + $TBANO_3$	RM1 + 2×1.5 equiv. (1+1 h heating)	$Pd_6\mathbf{L}_8$ + $Pd_{12}\mathbf{L}_{16}$	5.10×10^{-11}<br>5.58×10^{-11}
RM2	$Pd(NO_3)_2$:**L** = 3:4 in [D$_6$]-DMSO	$Pd_{12}\mathbf{L}_{16}$	4.98×10^{-11}
RM3	$Pd(NO_3)_2$:**L** = 3:4 in 5% (*v/v*) [D$_3$]-ACN in [D$_6$]-DMSO	$Pd_6\mathbf{L}_8$	6.24×10^{-11}
RM2 + $Pd(NO_3)_2$	RM2 + 3 equiv. $Pd(NO_3)_2$	$Pd_6\mathbf{L}_8$ + $Pd_{12}\mathbf{L}_{16}$	4.29×10^{-11}<br>5.62×10^{-11}
RM2 + [D$_3$]-ACN (postsynthetic add)	RM2 + 5% [D$_3$]-ACN	$Pd_6\mathbf{L}_8$	5.58×10^{-11}
RM2 (VT-NMR)	$Pd(NO_3)_2$:**L** = 3:4 in [D$_6$]-DMSO	$Pd_{12}\mathbf{L}_{16}$	4.96×10^{-11} (up to 125 °C)<br>5.10×10^{-11} (at 100 °C)
RM2 3:2	$Pd(NO_3)_2$:**L** = 3:2 in [D$_6$]-DMSO	$Pd_6\mathbf{L}_8$	5.98×10^{-11}
RM2 3:2 + **L**	RM2 3:2 + 2 equiv. **L**	$Pd_{12}\mathbf{L}_{16}$	4.29×10^{-11}
Neat sample of **L**	**L** in [D$_6$]-DMSO	**L**	1.57×10^{-10}

3 Computational Models

Table S4. Pd-Pd distances in Pd_6L_8 from the geometry optimized model.

Pd-Pd	Distance (Å)
Pd_{C3-C7}- Pd_{C3-C7}	15.74
Pd_{C3-C7}- Pd_{C3-C7}	15.58
Pd_{C3-C7}- Pd_{C3-C7}	15.66
Pd_{C3-C7}- Pd_{C3-C7}	15.60
Pd_{C3-C7}-Pd_{C24}	18.41
Pd_{C3-C7}-Pd_{C24}	18.66
Pd_{C3-C7}-Pd_{C24}	18.52
Pd_{C3-C7}-Pd_{C24}	18.24
Pd_{C3-C7}-Pd_{C24}	18.63
Pd_{C3-C7}-Pd_{C24}	18.31
Pd_{C3-C7}-Pd_{C24}	18.08
Pd_{C3-C7}-Pd_{C24}	18.26

Table S5. Angles of the triangular faces of Pd_6L_8 from the geometry optimized model.

Pd_6L_8 (triangular face)	∠Pd_{C7}-Pd_{C3}-Pd_{C24} (°)	∠Pd_{C3}-Pd_{C7}-Pd_{C24} (°)	∠Pd_{C3}-Pd_{C24}-Pd_{C7} (°)
1	65.40	64.68	49.92
2	65.12	64.01	50.87
3	63.61	66.38	50.02
4	65.54	64.61	49.85
5	63.99	65.16	50.85
6	65.27	63.74	50.99
7	66.11	64.00	49.89
8	63.52	65.97	50.51

Table S6. Pd-Pd distances in $Pd_{12}L_{16}$ from the geometry optimized model.

Pd-Pd	Distance (Å)	Pd-Pd	Distance (Å)
Pd_{C3-C7}- Pd_{C3-C7}	20.96	Pd_{C3-C7}-Pd_{C24}	19.69
Pd_{C3-C7}- Pd_{C3-C7}	17.27	Pd_{C3-C7}-Pd_{C24}	19.92
Pd_{C3-C7}- Pd_{C3-C7}	20.95	Pd_{C3-C7}-Pd_{C24}	19.07
Pd_{C3-C7}- Pd_{C3-C7}	19.98	Pd_{C3-C7}-Pd_{C24}	19.77
Pd_{C3-C7}- Pd_{C3-C7}	20.72	Pd_{C3-C7}-Pd_{C24}	18.94
Pd_{C3-C7}- Pd_{C3-C7}	19.37	Pd_{C3-C7}-Pd_{C24}	20.00
Pd_{C3-C7}- Pd_{C3-C7}	20.75	Pd_{C3-C7}-Pd_{C24}	18.34
Pd_{C3-C7}- Pd_{C3-C7}	20.10	Pd_{C3-C7}-Pd_{C24}	20.86
Pd_{C3-C7}- Pd_{C3-C7}	20.48	Pd_{C3-C7}-Pd_{C24}	19.17
Pd_{C3-C7}- Pd_{C3-C7}	17.96	Pd_{C3-C7}-Pd_{C24}	20.05
Pd_{C3-C7}- Pd_{C3-C7}	20.08	Pd_{C3-C7}-Pd_{C24}	20.41
Pd_{C3-C7}- Pd_{C3-C7}	20.39	Pd_{C3-C7}-Pd_{C24}	18.58
Pd_{C3-C7}- Pd_{C3-C7}	20.01	Pd_{C3-C7}-Pd_{C24}	19.96
Pd_{C3-C7}- Pd_{C3-C7}	19.52	Pd_{C3-C7}-Pd_{C24}	20.71
Pd_{C3-C7}-Pd_{C24}	18.43	Pd_{C3-C7}-Pd_{C24}	19.05

Table S7. Angles of triangular faces of $Pd_{12}L_{16}$ from the geometry optimized model.

$Pd_{12}L_{16}$ (Triangular face)	$\angle Pd_{C7}$-Pd_{C3}-Pd_{C24}	$\angle Pd_{C3}$-Pd_{C7}-Pd_{C24}	$\angle Pd_{C3}$-Pd_{C24}-Pd_{C7}
1	66.88	59.39	53.73
2	58.61	57.54	63.85
3	57.10	61.29	61.60
4	54.57	57.49	67.95
5	66.02	61.05	52.93
6	53.75	60.39	65.86
7	64.00	55.49	60.51
8	55.39	60.39	64.22
9	58.67	62.43	58.90
10	56.14	63.11	60.75
11	62.84	60.17	56.99
12	63.24	55.24	61.52
13	55.23	63.38	61.39
14	65.26	60.27	54.47
15	61.17	59.35	59.49
16	61.96	59.75	58.29

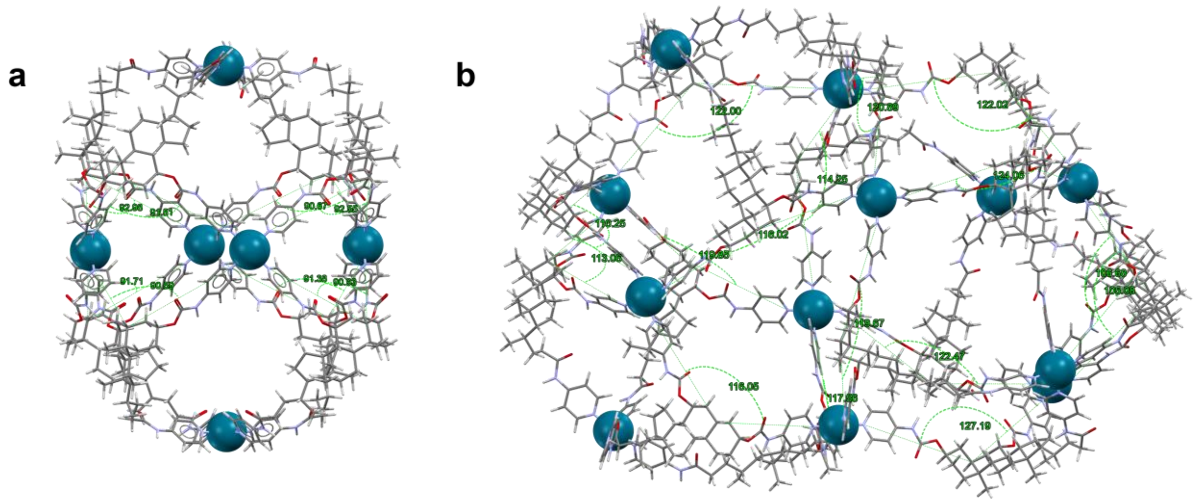

Figure S40. $\angle N_{Py(C3)}$—C5—$N_{Py(C7)}$ angle of a) $Pd_6\mathbf{L}_8$ and b) $Pd_{12}\mathbf{L}_{16}$.

Table S8. Comparison of intramolecular distances $N_{Py(C3)}$—$N_{Py(C7)}$, $N_{Py(C3)}$—$N_{Py(C24)}$, $N_{Py(C7)}$—$N_{Py(C24)}$ for models of **L**, Pd_6**L**$_8$, and Pd_{12}**L**$_{16}$.

	$N_{Py(C3)}$—$N_{Py(C7)}$ (Å)	$N_{Py(C3)}$—$N_{Py(C24)}$ (Å)	$N_{Py(C7)}$—$N_{Py(C24)}$ (Å)
**L (polar solvent)**	**14.29**	**21.74**	**15.94**
	13.56	16.80	15.85
	13.44	16.45	15.64
	13.51	16.27	15.78
**Pd_6L_8**	13.43	16.65	16.32
	13.42	16.85	16.20
	13.39	17.02	15.86
	13.50	16.67	16.04
	13.43	16.78	16.09
**Mean average:**	**13.46**	**16.69**	**15.97**
**Standard deviation:**	**0.06**	**0.24**	**0.23**
	15.16	16.22	16.57
	17.33	17.76	16.87
	17.01	17.75	16.56
	17.52	17.01	16.31
	15.23	17.05	16.66
	17.15	18.35	15.25
	16.38	16.55	16.86
	17.31	18.24	16.66
**$Pd_{12}L_{16}$**	17.00	18.42	17.88
	17.17	18.97	16.91
	15.76	17.17	16.92
	16.52	18.56	17.46
	16.83	17.56	17.59
	16.86	18.79	17.31
	16.40	17.86	16.54
	15.87	16.57	15.84
**Mean average:**	**16.59**	**17.68**	**16.76**
**Standard deviation:**	**0.74**	**0.85**	**0.65**

3.1 Helical Chirality

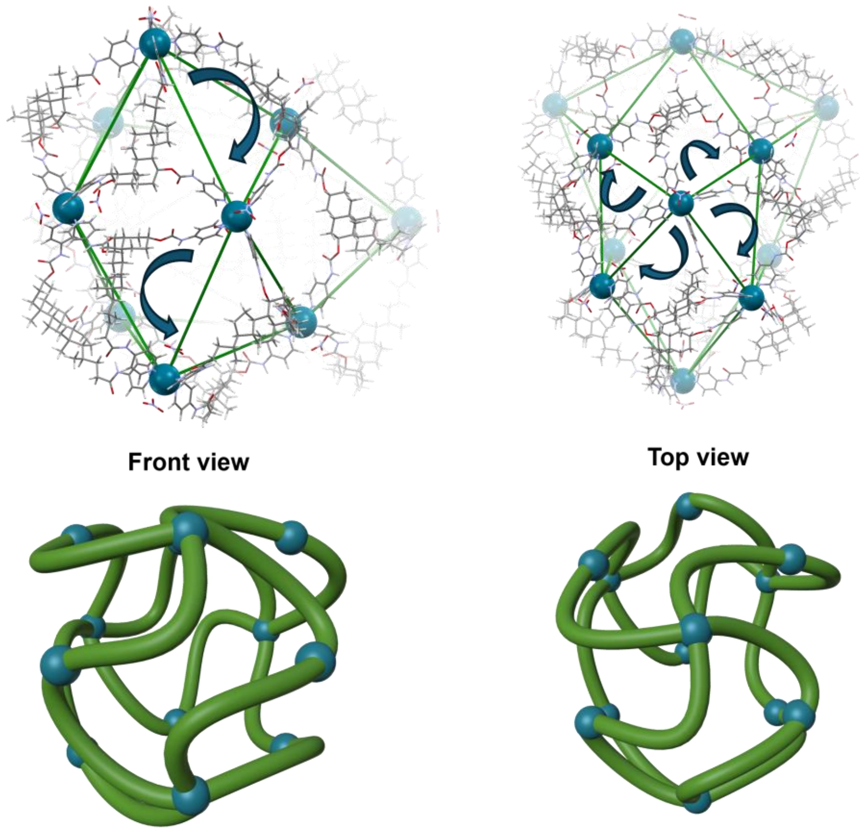

Figure S41. Right-handed octuple helix (ΔΔΔΔΔΔΔΔ) of Pd_{12}**L**$_{16}$.

Using the molecular orbital analysis we found that electronic transitions in range of 240-340 nm are localized only on a small fragment of **L**, mainly on the pyridyl residues. In fragment-based approach, the pyridyl moieties (*i.e.*, $(CH_3)_2$-CH-O-CO-NH-Py and CH_3-CH_2-CO-NH-Py) of the complex, Pd atoms, and NO_3^- molecules form an active part of the Pd_{12}**L**$_{16}$ complex in the TD CAM-B3LYP calculation leading to CD spectrum well-fitting to the experimental one (Fig. 4a). The small deviations are acceptable considering the size and complexity of the self-assembly as well as computational challenges in the accurate description of the metal-to-ligand charge transfer states of the complex. Spatial organization of these fragments connected into continuous helix by the rest of bile acid molecule within the Pd_6**L**$_8$ and Pd_{12}**L**$_{16}$ complexes thus result in the shown helicity. This spectroscopic feature thus represents reliable, swift, and effective tool for structural elucidation of helical SCCs.
A number of SCCs has been synthesized using helical ligands, *e.g.*, those derived from BINOL[9] or helicine.[10,11] The spectroscopic response following the CD spectra is usually less significant on the level of a single ligand, but it increases in the structure of SCC. In general, the signals follow a trend for right-handed helices (P or Δ) showing a positive Cotton effect, (+ -) couplet, and for left-handed helices (M or Λ) showing a negative Cotton effect, (- +) couplet, in the range of 220-400 nm for nitrogen-containing heterocyclic aromatic systems, depending on their substitution. In our case, the ligand has a weak single negative CD band at 247 nm in the spectral region studied. The

helical structure of **L** might not be obvious at first sight, but it becomes more profound when following on two consecutive ligands interconnected through Pd-coordination bond (Fig. 4), forming right-handed helix.

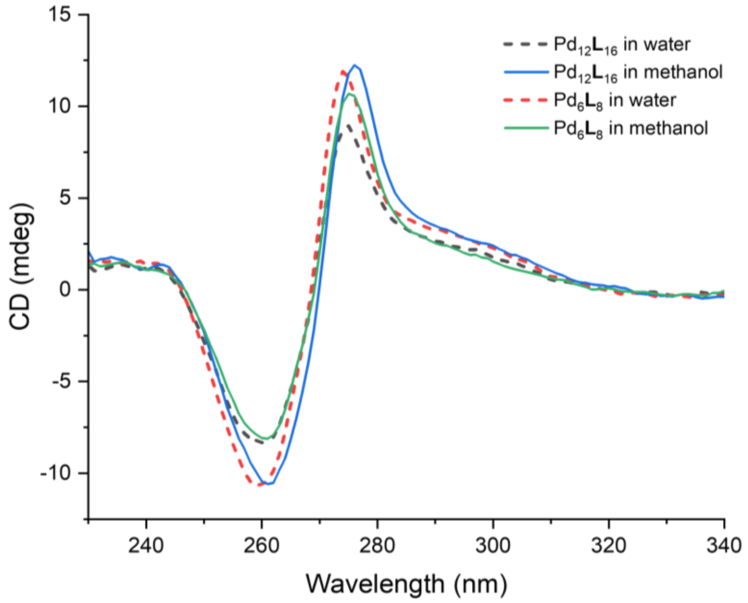

Figure S42. Comparison of CD spectra of Pd_{12}**L**$_{16}$ and Pd_6**L**$_8$ in 1000:1 MeOH:[D6]-DMSO and H_2O:[D6]-DMSO at 25 °C (dilution of 0.63 mM and 1.25 mM [D6]-DMSO solution of Pd_{12}**L**$_{16}$ and Pd_6**L**$_8$, respectively).

4 Monodentate Ligands and their Complexes with $Pd(NO_3)_2$

4.1 Synthesis of **L$_{M24}$**

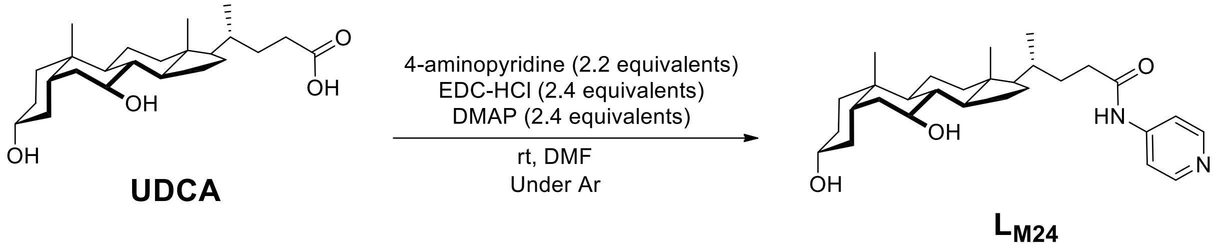

Scheme S2. Synthesis of **L$_{M24}$**.

L$_{M24}$ was synthesized from UDCA (100 mg, 3 mL DMF) in a single step following procedure in Section 2.1 of SI. The product was purified by column chromatography on silica gel using 10:1 dichloromethane:acetone as a mobile phase. The product was isolated as a white solid in 25 % yield (30 mg).

HRMS (*m/z*) for $[M+H]^+$ = $[C_{29}H_{45}N_2O_3]^+$: theoretical mass 469.3425, found experimental mass 469.3421, mass accuracy 0.4 ppm.

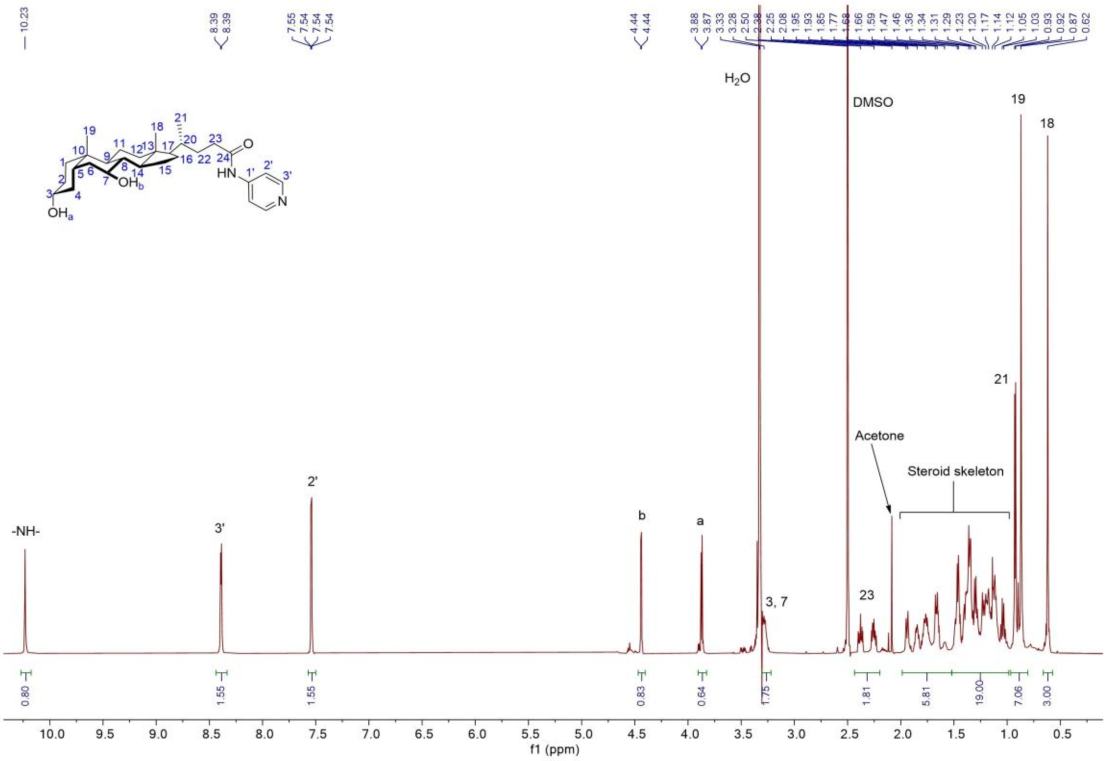

Figure S43. ^{1}H NMR spectrum of **L$_{M24}$** measured in [D$_6$]-DMSO at 700 MHz and 298.2 K.

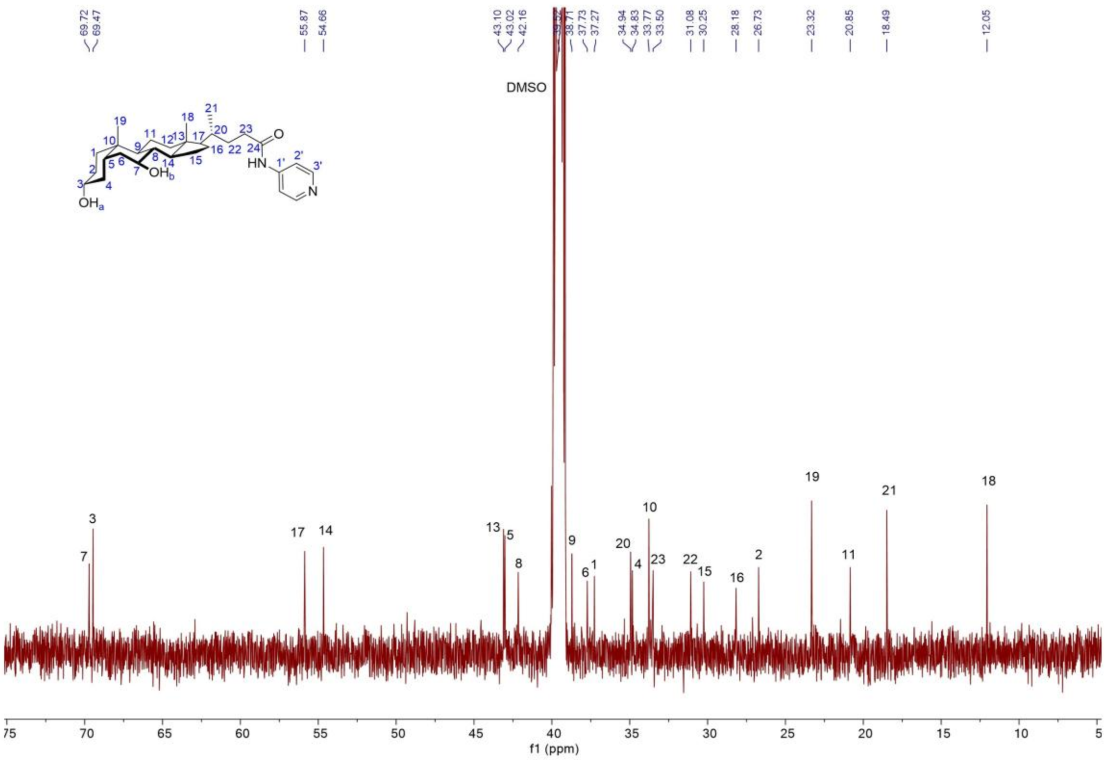

Figure S44. ^{13}C NMR spectrum of **L$_{M24}$** (aliphatic region) measured in [D$_6$]-DMSO at 175 MHz and 298.2 K.

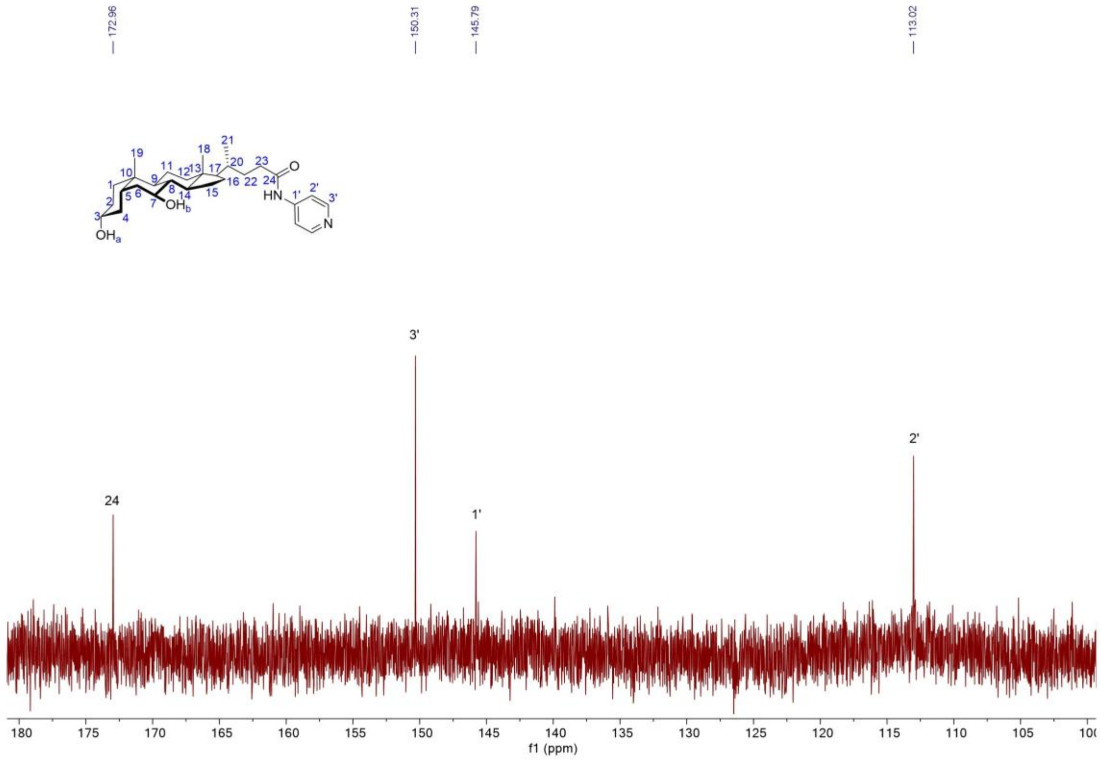

Figure S45. ^{13}C NMR spectrum of **L$_{M24}$** (aromatic and carbonyl region) measured in [D$_6$]-DMSO at 175 MHz and 298.2 K.

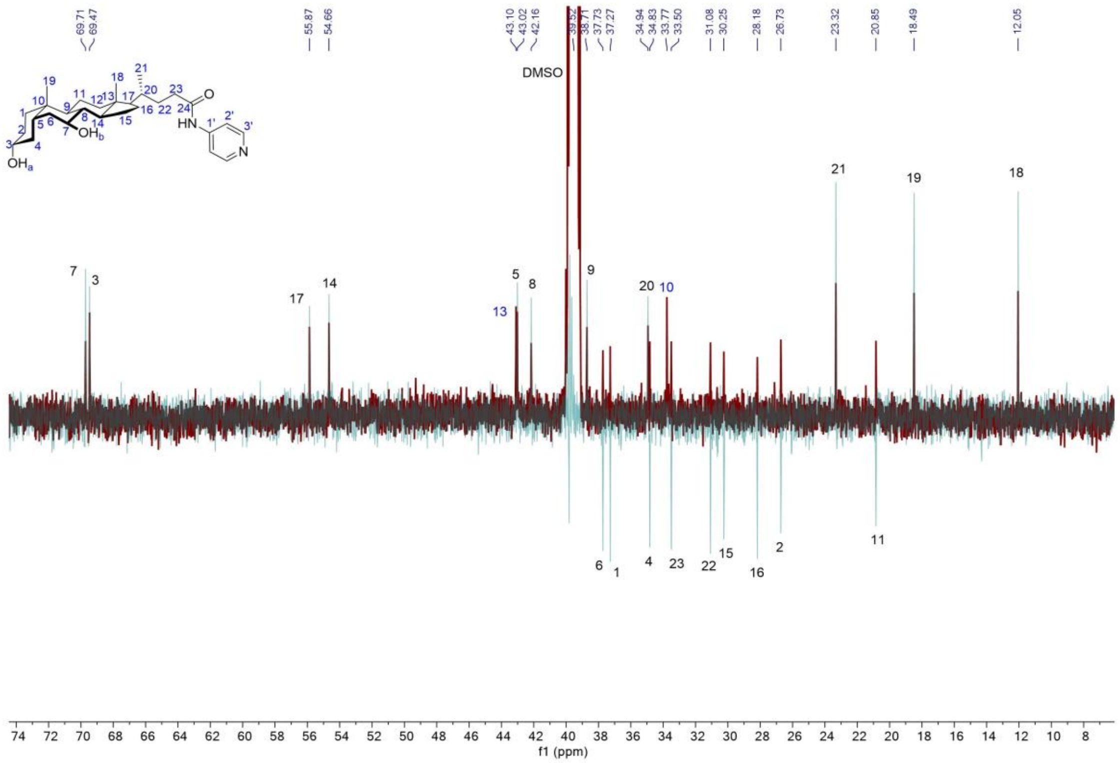

Figure S46. Comparison of ^{13}C and DEPT-135 NMR spectrum (aliphatic region) of **L$_{M24}$** measured in [D$_6$]-DMSO at 175 MHz at 298.2 K.

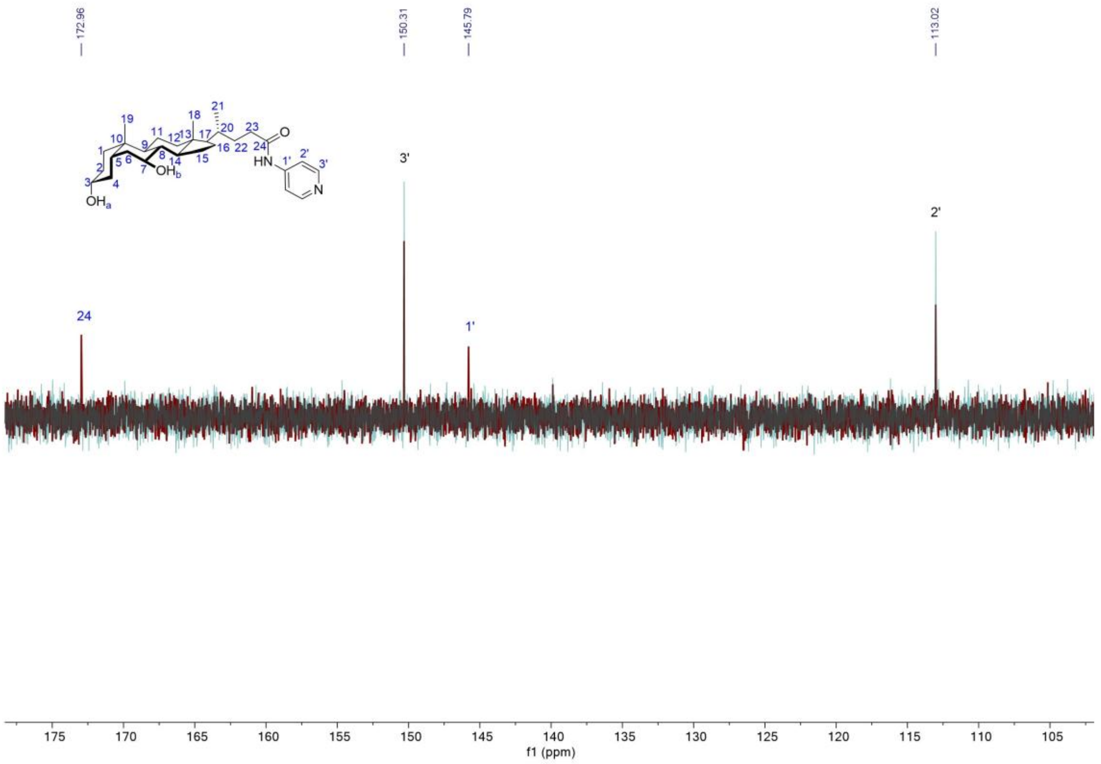

Figure S47. Comparison of ^{13}C and DEPT-135 NMR spectrum (aromatic and carbonyl region) of **L$_{M24}$** measured in [D$_6$]-DMSO at 175 MHz at 298.2 K.

4.2 Synthesis of **L$_{M3}$**

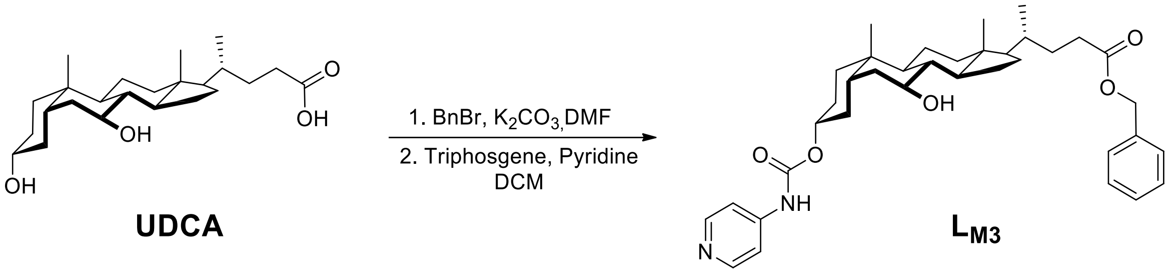

Scheme S3. Synthesis of **L$_{M3}$**.

L$_{M3}$ was synthesized in two steps from UDCA (392 mg) using a previously reported synthetic protocol.[6] The product was isolated as white solid in 47 % yield (260 mg).

HRMS (*m/z*) for $[M-H]^-$ = $[C_{37}H_{49}N_2O_5]^-$: theoretical mass 601.3647, found experimental mass 601.3650, mass accuracy 0.5 ppm.

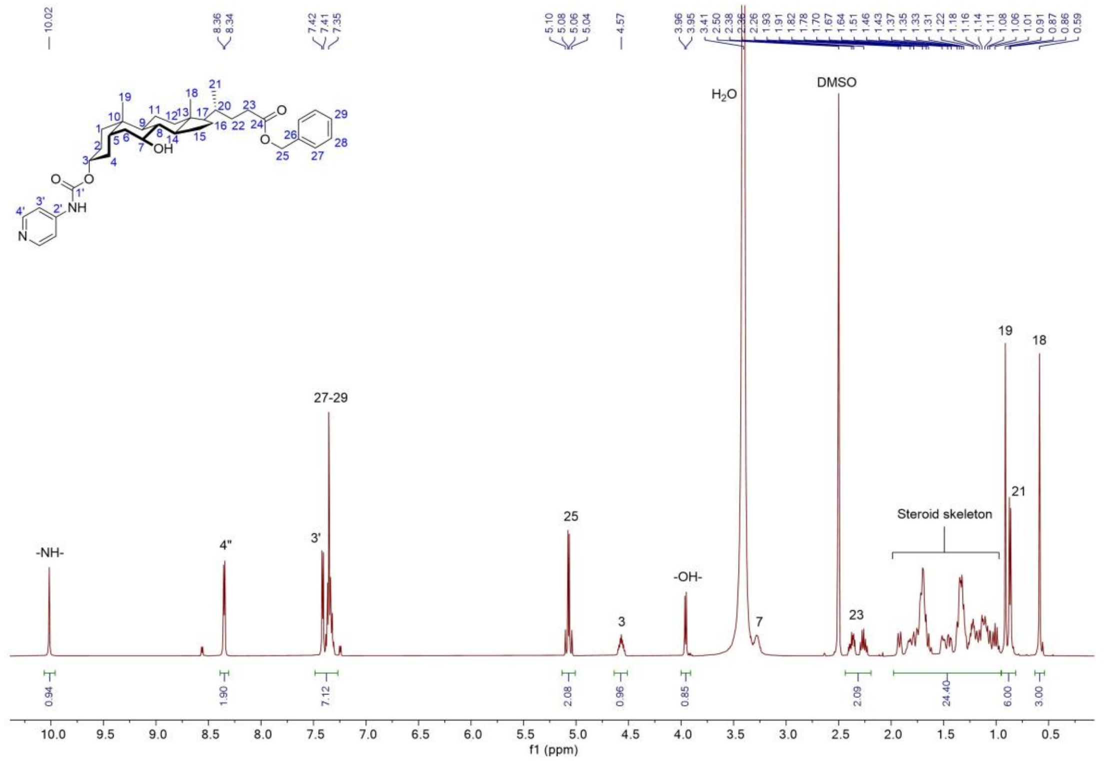

Figure S48. ^{1}H NMR spectrum of **L$_{M3}$** measured in [D$_6$]-DMSO at 700 MHz and 298.2 K.

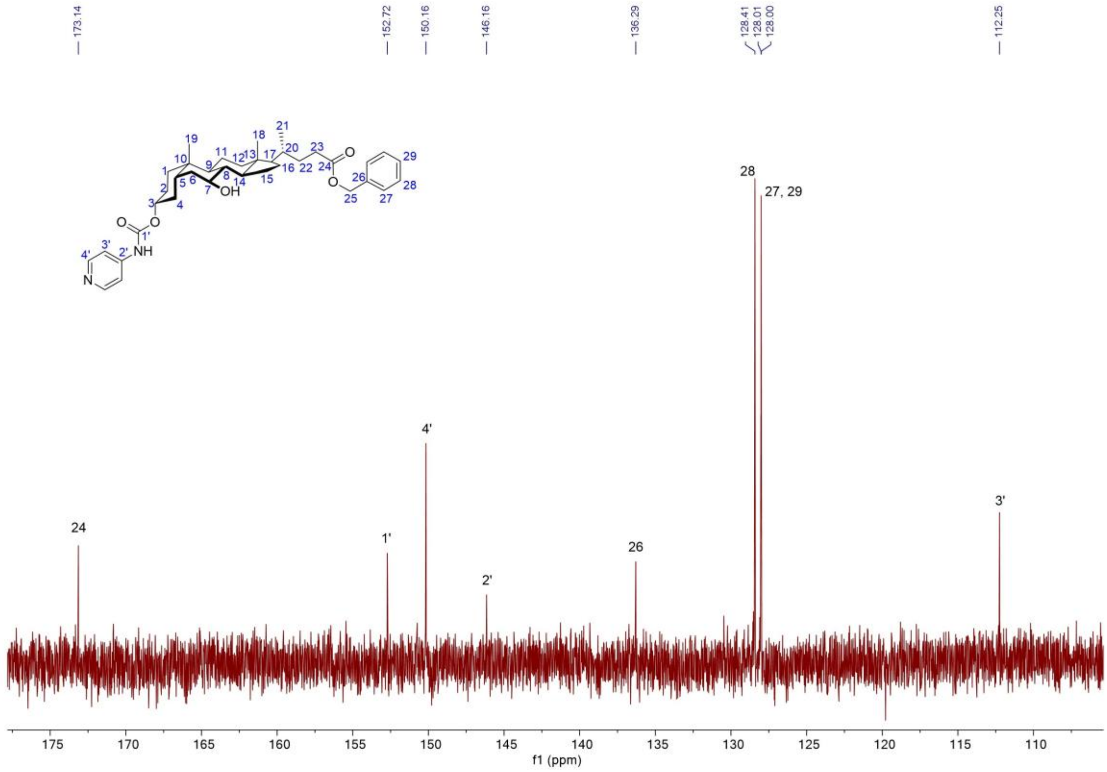

Figure S49. ^{13}C NMR spectrum of **L$_{M3}$** (aromatic and carbonyl region) measured in [D$_6$]-DMSO at 175 MHz and 298.2 K.

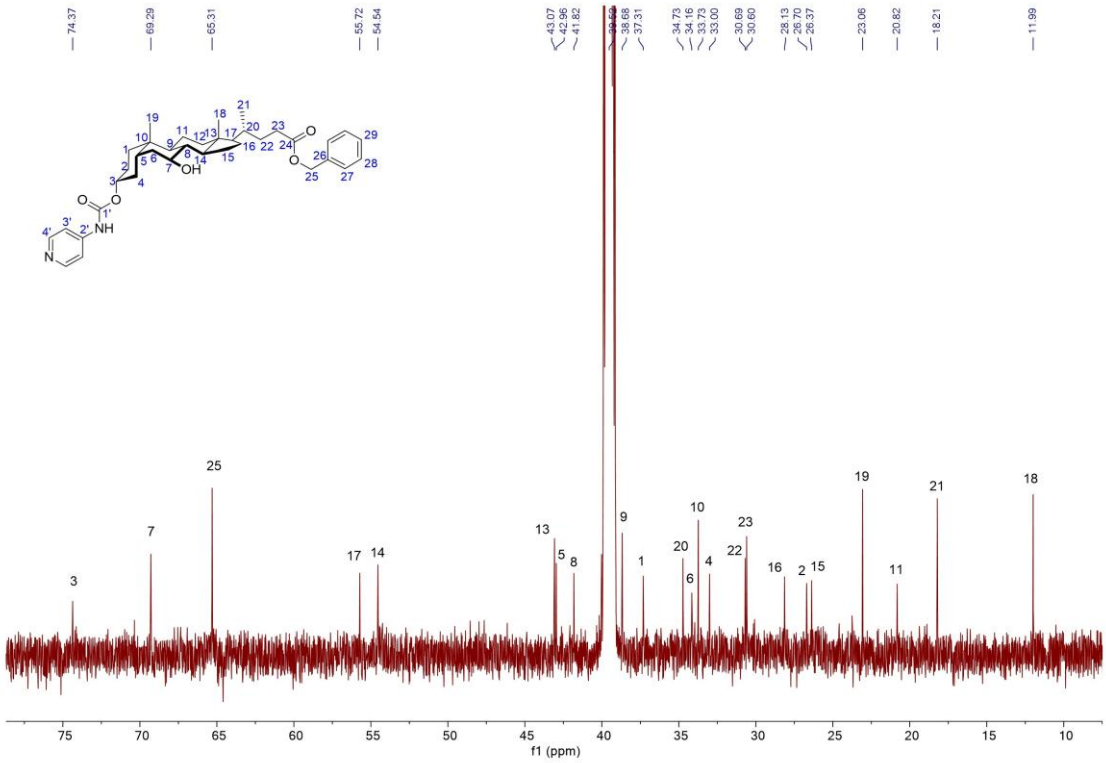

Figure S50. ^{13}C NMR spectrum of **L$_{M3}$** (aliphatic region) measured in [D$_6$]-DMSO at 175 MHz and 298.2 K.

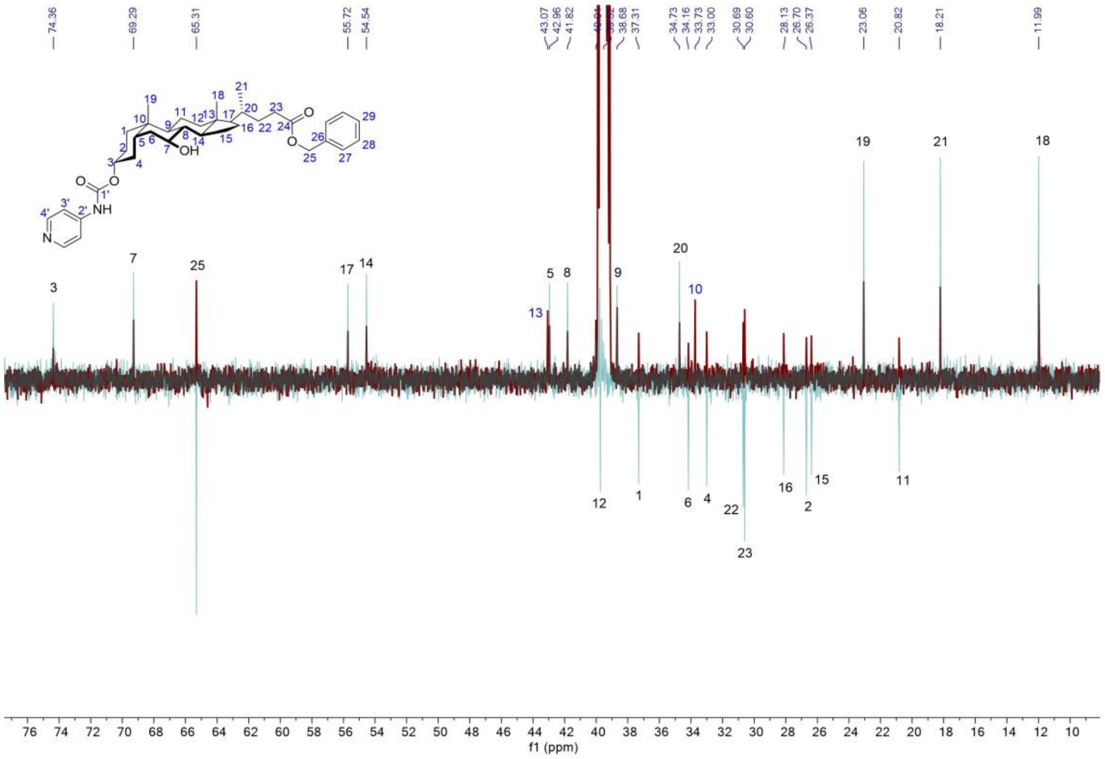

Figure S51. Comparison of ^{13}C and DEPT-135 NMR spectrum (aliphatic region) of **L$_{M3}$** measured in [D$_6$]-DMSO at 175 MHz at 298.2 K.

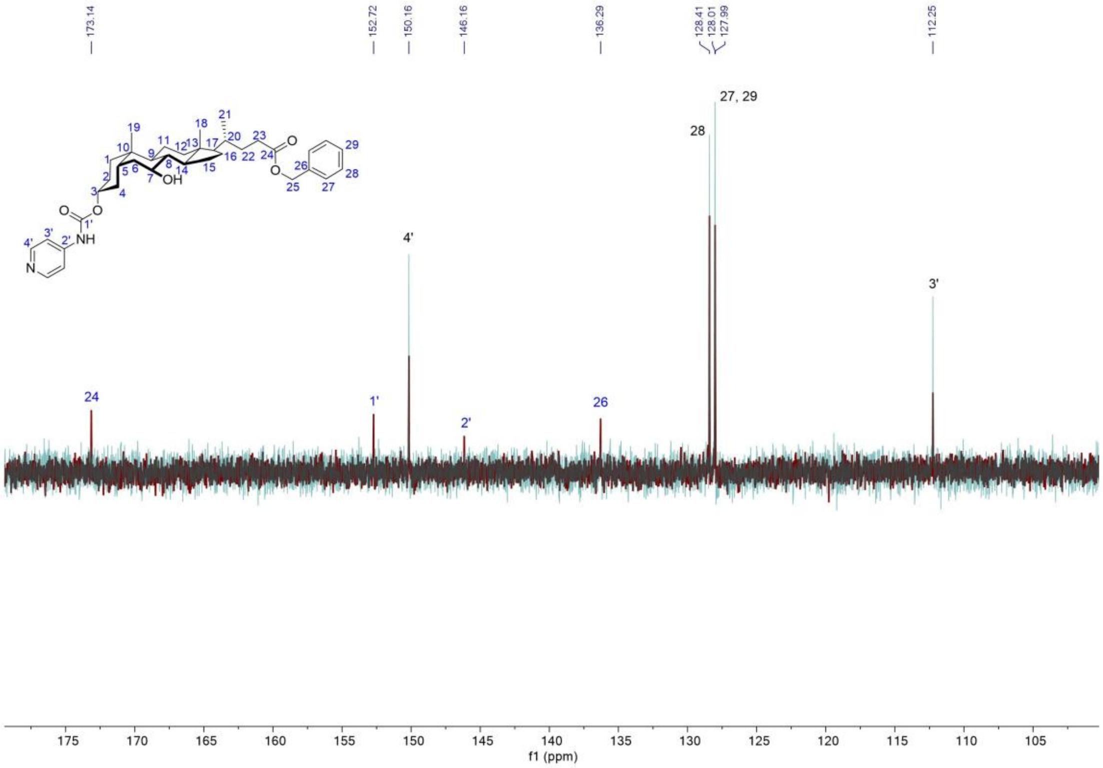

Figure S52. Comparison of ^{13}C and DEPT-135 NMR spectrum (aromatic and carbonyl region) of **L_{M3}** measured in [D_6]-DMSO at 175 MHz at 298.2 K.

4.3 Coordination of **L_{M3}** and **L_{M24}** with $Pd(NO_3)_2$

The coordination reaction of **L_{M3}** and **L_{M24}** with $Pd(NO_3)_2$ provided complexes as confirmed by ^{1}H NMR, ^{1}H DOSY NMR spectroscopy, and MS. We expect that these monodentate ligands bound to a single central Pd(II) will possess larger rotational freedom of axes leading to different conformational isomers in various representations than those locked inside the structures of cages. Both coordinated ligands provided similarly broad ^{1}H NMR pyridyl patterns to those of cages, but they were sharper in shape (being smaller complexes) and individual representation of signal sets appeared to be in different ratios (Fig. S53-S55) suggesting that: 1) the coordinated ligands vary in their conformation, or 2) there are various complexes, *i.e.*, PdL, PdL_2, PdL_3, and PdL_4. The ^{1}H DOSY NMR spectroscopy shows several coordination complexes (Fig. S56) which can again differ from each other either by their conformations, thus in their 3D organization and diffusion coefficients, or in their molecular size. The differences in diffusion coefficients of these species are small, suggesting that rather various conformers are present. Finally, the ESI-MS shows molecular peaks of [PdL_4], but also smaller complexes containing fewer ligands, which could be considered either as fragments of the large tetrameric complex (which is less stable because of the lack of structural stabilization by multiple coordination bonds as present in cages) or unique molecular peaks of variously substituted Pd(II). Based on these data and the previously obtained data on cages, we lean on the side of the presence of relatively stable conformational isomers.

MS (*m/z*) for $[(\mathbf{L_{M3}})_4Pd]^{2+}$: theoretical mass 1257.6951, experimental mass 1257.6997.
MS (*m/z*) for $[(\mathbf{L_{M24}})_4Pd]^{2+}$: theoretical mass 989.6216, experimental mass 989.6122.

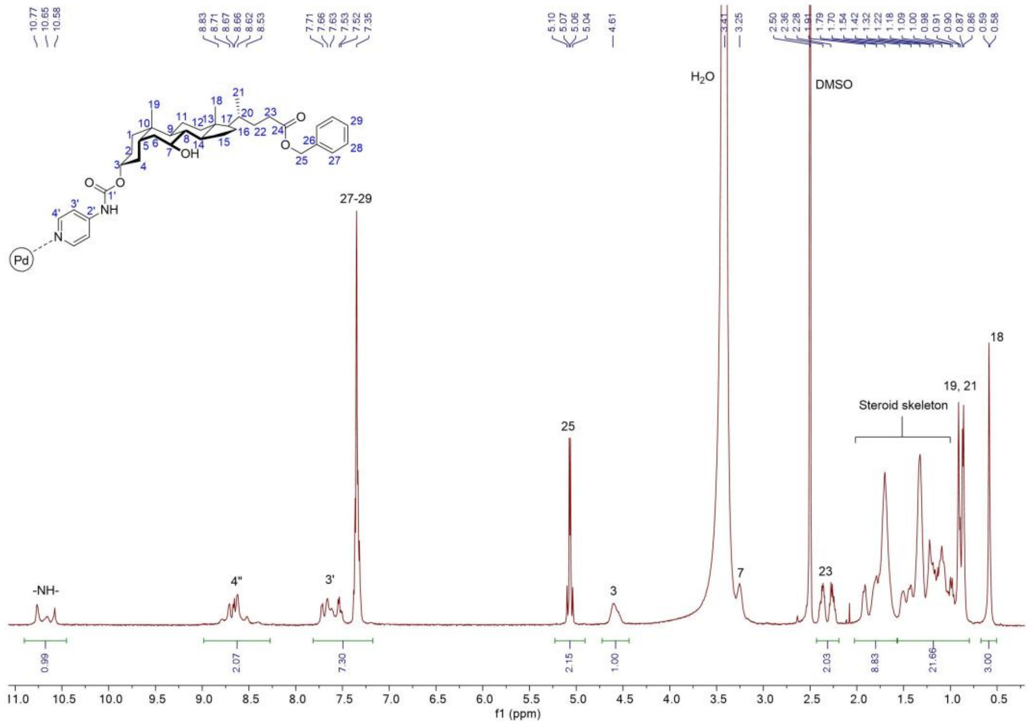

Figure S53. ^{1}H NMR spectrum of **L$_{M3}$** upon coordination with Pd(NO$_3$)$_2$ measured in [D$_6$]-DMSO at 700 MHz and 298.2 K.

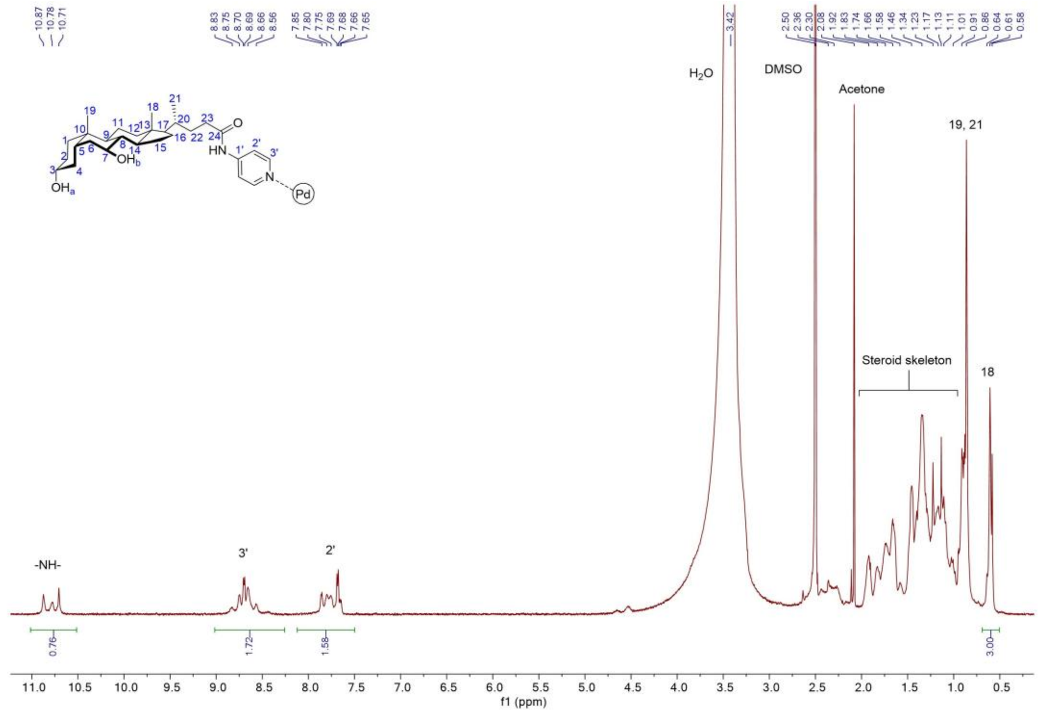

Figure S54. ^{1}H NMR spectrum of **L$_{M24}$** upon coordination with Pd(NO$_3$)$_2$ measured in [D$_6$]-DMSO at 700 MHz and 298.2 K.

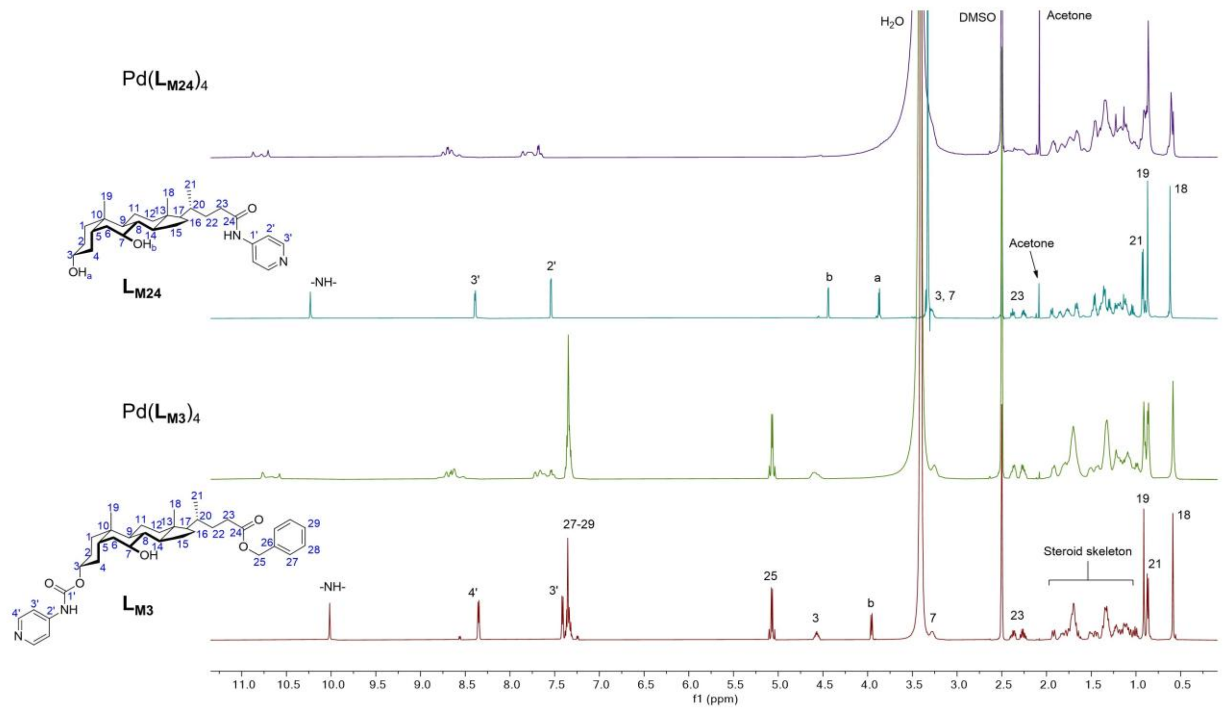

Figure S55. Comparison of ¹H NMR spectra of $\mathbf{L_{M3}}$ and $\mathbf{L_{M24}}$ and their coordination products with $Pd(NO_3)_2$ measured in [D6]-DMSO at 700 MHz and 298.2 K.

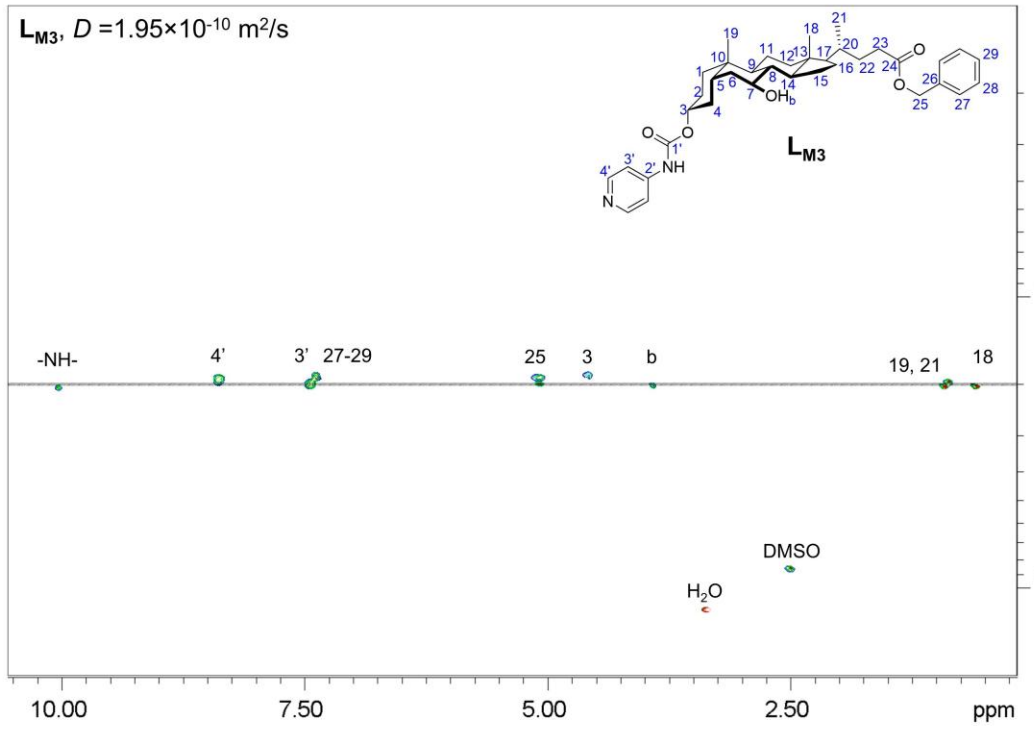

a)

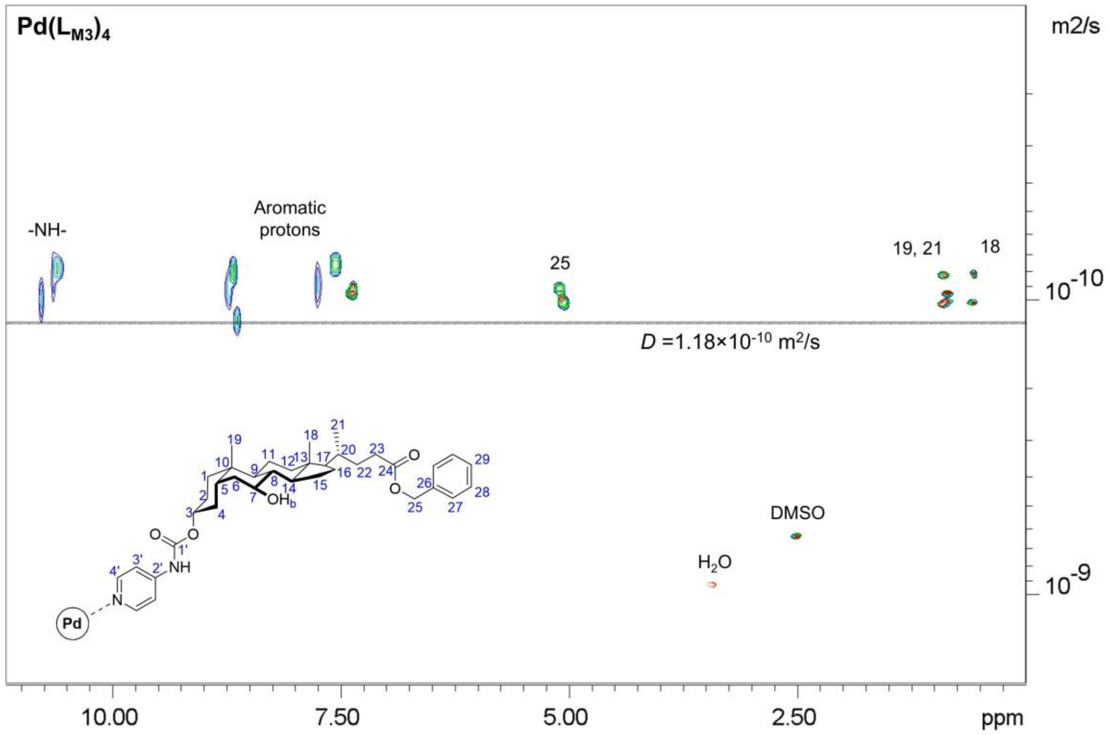

Pd(L$_{M3}$)$_4$
m2/s
-NH-
Aromatic protons
25
19, 21
18
10^{-10}
D =1.18×10^{-10} m^2/s
DMSO
H_2O
10^{-9}
Pd
10.00
7.50
5.00
2.50
ppm

b)

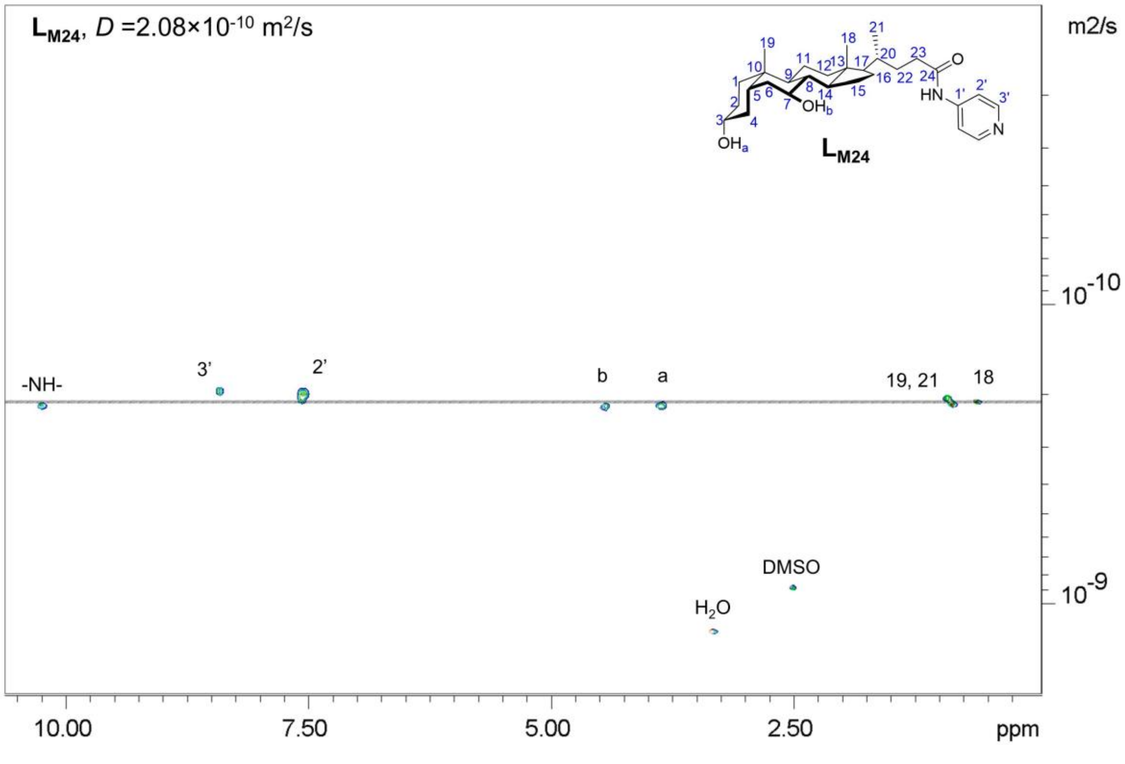

L$_{M24}$, D =2.08×10^{-10} m^2/s
m2/s
L$_{M24}$
10^{-10}
-NH-
3'
2'
b
a
19, 21
18
DMSO
H_2O
10^{-9}
10.00
7.50
5.00
2.50
ppm

c)

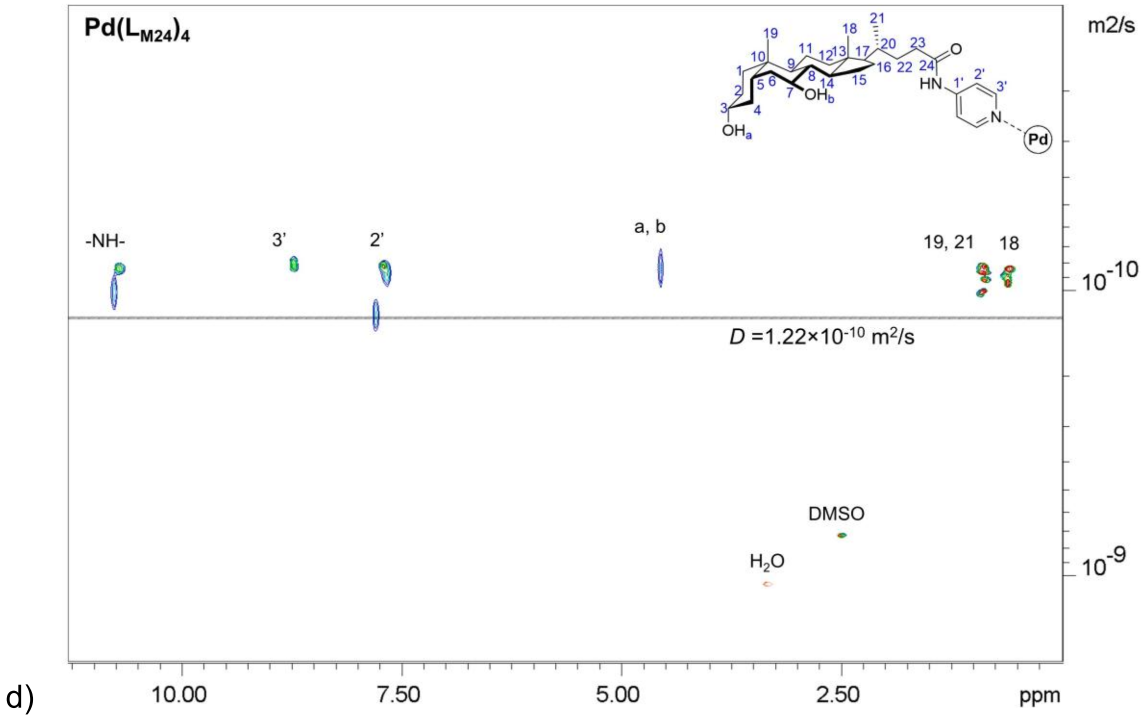

Figure S56. ^{1}H DOSY NMR: a) **LM3**, b) **LM3** coordination products with Pd(NO3)2, c) **LM24**, and d) its coordination products. The spectra were measured in [D6]-DMSO at 700 MHz and 298.2 K.

5 Hepatotoxicity and Activity Evaluation

	Nominal concentrations					Spheroid biological response (8-d exposure)						Pd content (ICP-MS analysis)				Pd bioavailability (%) [2]		Pd bio-concentration factor [3]	
	Compound concentration		Pd^{2+} concentration		Pd^{2+} dose (ng/well)	Spheroid volume (nL)		Spheroid volume (%control)		Viability (ATP, %control)		Pd content (ng/spheroid)		Intra-spheroid Pd concentration (ng/µL) [1]					
	Molar (µM)	Mass (ng/µL)	Molar (µM)	Mass (ng/µL)		Mean	SD	Mean	SD	Mean	SD	Mean	SD	Mean	SD	Mean	SD	Mean	SD
Negative control	-	-	-	**-**	**-**	**13.798**	4.275	**100%**	31%	**100%**	16%	***n.a.***	*n.a.*	***n.a.***	*n.a.*	***n.a.***	*n.a.*	***n.a.***	*n.a.*
Solvent control	-	-	-	**-**	**-**	**12.633**	1.623	**92%**	12%	**100%**	15%	**0.005**[4]	*n.a.*	**0.4**[4]	*n.a.*	***n.a.***	*n.a.*	***n.a.***	*n.a.*
Ligand	8	5.667	-	**-**	**-**	**13.149**	0.311	**95%**	2%	**102%**	12%	***n.a.***	*n.a.*	***n.a.***	*n.a.*	***n.a.***	*n.a.*	***n.a.***	*n.a.*
	32	22.669	-	**-**	**-**	**13.138**	4.566	**95%**	33%	**96%**	8%	***n.a.***	*n.a.*	***n.a.***	*n.a.*	***n.a.***	*n.a.*	***n.a.***	*n.a.*
$Pd(NO_3)_2 \cdot 2H_2O$	6	1.599	6	**0.639**	**160**	**13.054**	1.878	**95%**	14%	**100%**	10%	**0.017**	0.006	**1.3**	0.4	**0.010**	0.004	**2.0**	0.7
	24	6.395	24	**2.554**	**639**	**13.255**	4.410	**96%**	32%	**90%**	11%	**0.150**	0.075	**11.3**	5.7	**0.023**	0.012	**4.4**	2.2
Pd_6L_8	1	6.303	6	**0.639**	**160**	**13.972**	1.601	**101%**	12%	**97%**	8%	**0.013**	0.006	**1.0**	0.4	**0.008**	0.004	**1.5**	0.6
	4	25.210	24	**2.554**	**639**	**11.408**	2.205	**83%**	16%	**78%**	10%	**1.500**	0.127	**131.5**	11.2	**0.235**	0.020	**51.5**	4.4
$Pd_{12}L_{16}$	0.5	6.303	6	**0.639**	**160**	**13.606**	1.198	**99%**	9%	**88%**	4%	**0.070**	0.035	**5.1**	2.5	**0.044**	0.022	**8.1**	4.0
	2	25.210	24	**2.554**	**639**	**6.713**	3.294	**49%**	24%	**65%**	1%	**4.260**	2.362	**634.6**	351.8	**0.667**	0.370	**248.5**	137.8

1) The concentration of Pd^{2+} inside the spheroids (ng/µL) was calculated from the ICP-MS analysis of Pd^{2+} content per spheroid (ng/spheroid) and the spheroid volume (nL), which was determined from spheroid microphotographs

2) Percentage of measured Pd^{2+} content in each spheroid (ng/spheroid) relative to the initial nominal Pd^{2+} dose per well (ng/well).

3) The ratio of the calculated intra-spheroid concentration (spheroid Pd content per spheroid volume, IC, ng/µL) to the initial nominal extracellular Pd^{2+} concentration (EC, ng/µL).

4) Replaced with LOD/2 (limit of quantification, 10 pg/spheroid)

Table S9. Summary of treatments of HepG2 spheroids and their responses after 8-d exposure.

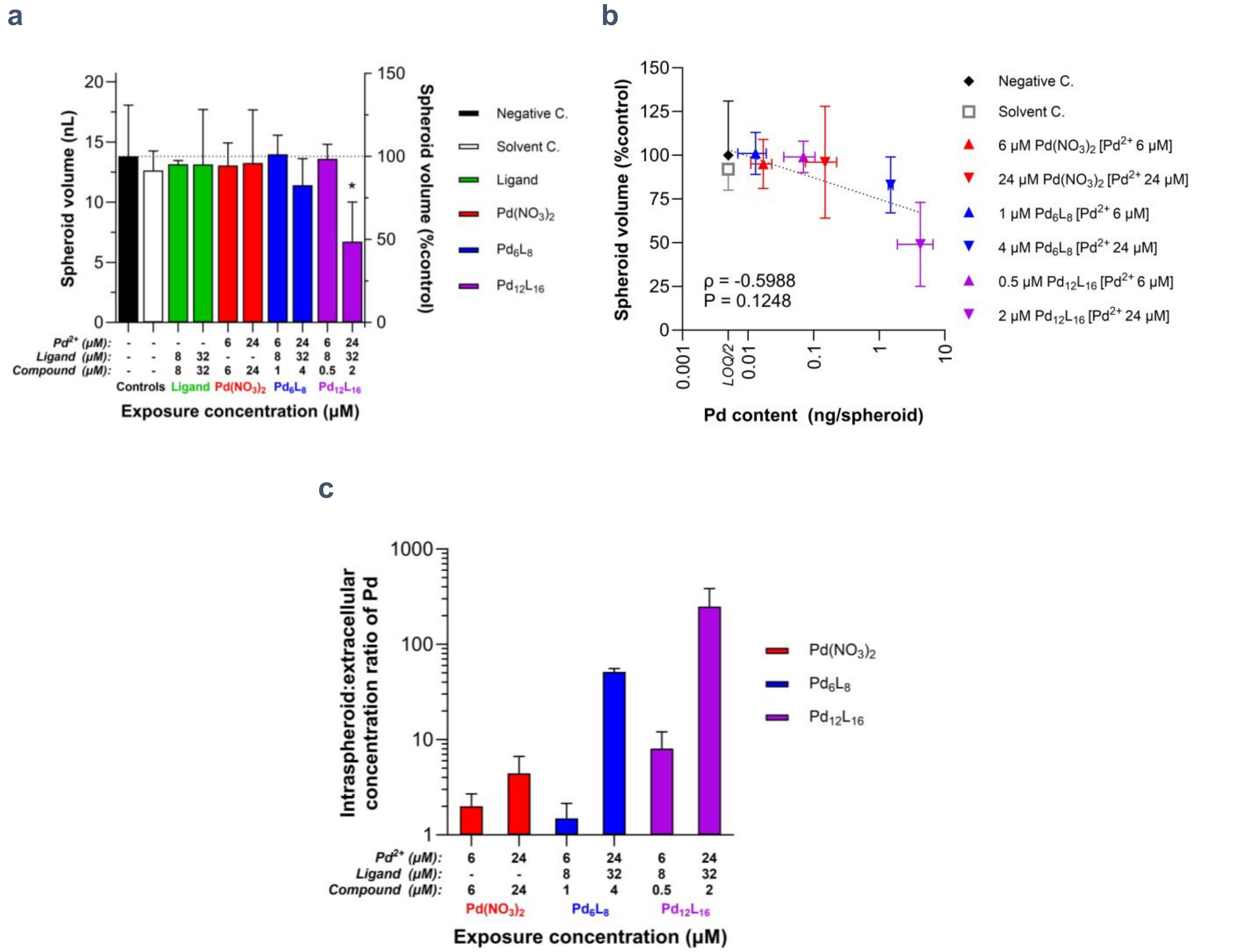

Figure S57. Toxicological studies of the SCCs. a, Concentration-response of HepG2 spheroid volume after 8 days exposure to $Pd(NO_3)_2$, **L**, Pd_6**L**$_8$, and Pd_{12}**L**$_{16}$ for 8 days. Asterisk (*) indicates statistically significant ($P < 0.05$) difference from the solvent control. b, Relation of relative spheroid volume to palladium amount measured in spheroids. ρ represents Spearman's rank correlation coefficient with a P value. c, Intraspheroid:extracellular concentration ratio of Pd (ratio of the intraspheroid Pd concentration after 8 days of exposure to the nominal initial extracellular Pd^{2+} concentration).

6 References

1. Stow, S. M.; Causon, T. J.; Zheng, X.; Kurulugama, R. T.; Mairinger, T.; May, J. C.; Rennie, E. E.; Baker, E. S.; Smith, R. D.; McLean, J. A.; Hann, S.; Fjeldsted, J. C. An Interlaboratory Evaluation of Drift Tube Ion Mobility–Mass Spectrometry Collision Cross Section Measurements *Anal. Chem.* **89**, 9048-9055 (2017).

2. Gabelica, V.; Shvartsburg, A. A.; Afonso, C.; Barran, P.; Benesch, J. L. P.; Bleiholder, C.; Bowers M. T.; Bilbao, A.; Bush, M. F.; Campbell, J. L.; Campuzano, I. D. G.; Causon, T.; Clowers, B. H.; Creaser, C. S.; Pauw, E. D.; Far, J.; Fernandez-Lima, F.; Fjeldsted, J. C.; Giles, K.; Groessl, M.; Hogan Jr., C. J.; Hann, S.; Kim, H. I.; Kurulugama, R. T.; May, J. C.; McLean, J. A.; Pagel, K.; Richardson, K.; Ridgeway, M. E.; Rosu, F.; Sobott, F.; Thalassinos, K.; Valentine,

S. J.; Wyttenbach, T. Recommendations for Reporting Ion Mobility Mass Spectrometry Measurements *Mass Spectrom. Rev*. **38**, 291–320 (2019).

3. Frisch, M.J.; Trucks, G.W.; Schlegel, H.B.; Scuseria, G.E.; Robb, M.A.; Cheeseman, J.R.; Scalmani, G.; Barone, V.; Mennucci, B.; Petersson, G.A.; *et al.* Gaussian 09, Revision D.01. http://www.rsc.org/suppdata/c5/sc/c5sc02423d/c5sc02423d1.pdf (2013).

4. Linnanto, J.; Korppi-Tommola, J. Structural and Spectroscopic Properties of Mg-Bacteriochlorin and Methyl Bacteriochlorophyllides a, b, g, and h Studied by Semiempirical, ab Initio, and Density Functional Molecular Orbitl Methods. *J. Phys. Chem. A* 108, 5872–5882 (2004).

5. Schneider, M., Grossi, M. F., Gadara, D., Spáčil, Z., Babica, P., Bláha, L. Treatment of cylindrospermopsin by hydroxyl and sulfate radicals: Does degradation equal detoxification? *J. Hazard. Mater.* **424**,127447 (2022).

6. Jurček, O. *et al.* Superchiral Pd_3L_6 Coordination Complex and Its Reversible Structural Conversion into $Pd_3L_3Cl_6$ Metallocycles. *Angew. Chem. Int. Ed.* **54**, 15462–15467 (2015).

7. Jurček, O. *et al.* Hexagonal Microparticles from Hierarchical Self-Organization of Chiral Trigonal Pd_3L_6 Macrotetracycles. *Cell Rep. Phys. Sci.* **2**, 100303 (2021).

8. Jurček, O. *et al.* Unsymmetric Chiral Ligands for Large Metallo-Macrocycles: Selectivity of Orientational Self-Sorting. *Angew. Chem. Int. Ed.* DOI:10.1002/ anie.202409134 (2024).

9. Christoph, G. *et al.* Enantiomerically Pure [M_6L_{12}] or [$M_{12}L_{24}$] Polyhedra from Flexible Bis(Pyridine) Ligands *Angew. Chem. Int. Ed.* **53**, 1693 –1698 (2014).

10. Schulte, T. R., Holstein, J. J., Clever, G. H. Chiral Self-Discrimination and Guest Recognition in Helicene-Based Coordination Cages *Angew. Chem. Int. Ed.* **58**, 5562–5566, (2019).

11. Wu, K., Tessarolo, J., Baksi, A., Clever, G. H. Guest-Modulated Circularly Polarized Luminescence by Ligand-to-Ligand Chirality Transfer in Heteroleptic Pd^{II} Coordination Cages *Angew. Chem. Int. Ed.* **61**, e202205725 (2022).
